# *N*-Edited Guanine Isosteres

**DOI:** 10.1021/acs.joc.3c00467

**Published:** 2023-07-11

**Authors:** Xin Li, Marina Diguele Romero, Sona Tcaturian, Katarzyna Kurpiewska, Alexander Dömling

**Affiliations:** † Department of Drug Design, University of Groningen, A. Deusinglaan 1, 9713 AV Groningen, The Netherlands; ‡ Department of Crystal Chemistry and Crystal Physics Faculty of Chemistry, Jagiellonian University, 30-387 Kraków, Poland

## Abstract

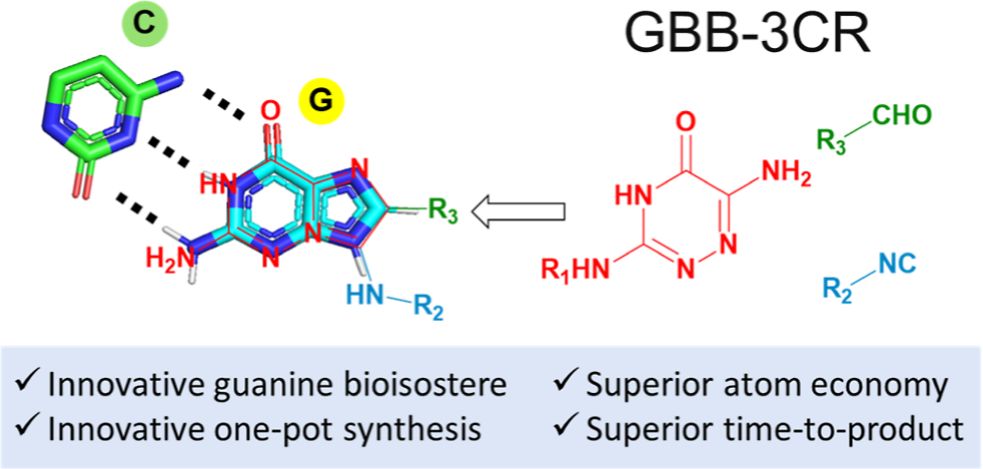

Guanine is one out of five endogenous nucleobases and of key interest in drug discovery and chemical biology. Hitherto, the synthesis of guanine derivatives involves lengthy multistep sequential synthesis of low overall diversity, resulting in the quest for innovation. Using a “single-atom skeletal editing” approach, we designed 2-aminoimidazo[2,1-*f*][1,2,4]triazin-4(3*H*)-one as a guanine isostere, conserving the biologically important HBA–HBD–HBD (HBA = hydrogen bond acceptor; HBD = hydrogen bond donor) substructure. We realized our design by a simple one-pot two-step method combining the Groebke-Blackburn-Bienaymé reaction (GBB-3CR) and a deprotection reaction to assemble the innovative guanine isosteres in moderate to good yields. Our innovative, diverse, short, and reliable multicomponent reaction synthesis will add to the toolbox of guanine isostere syntheses.

## Introduction

**Figure 1:**
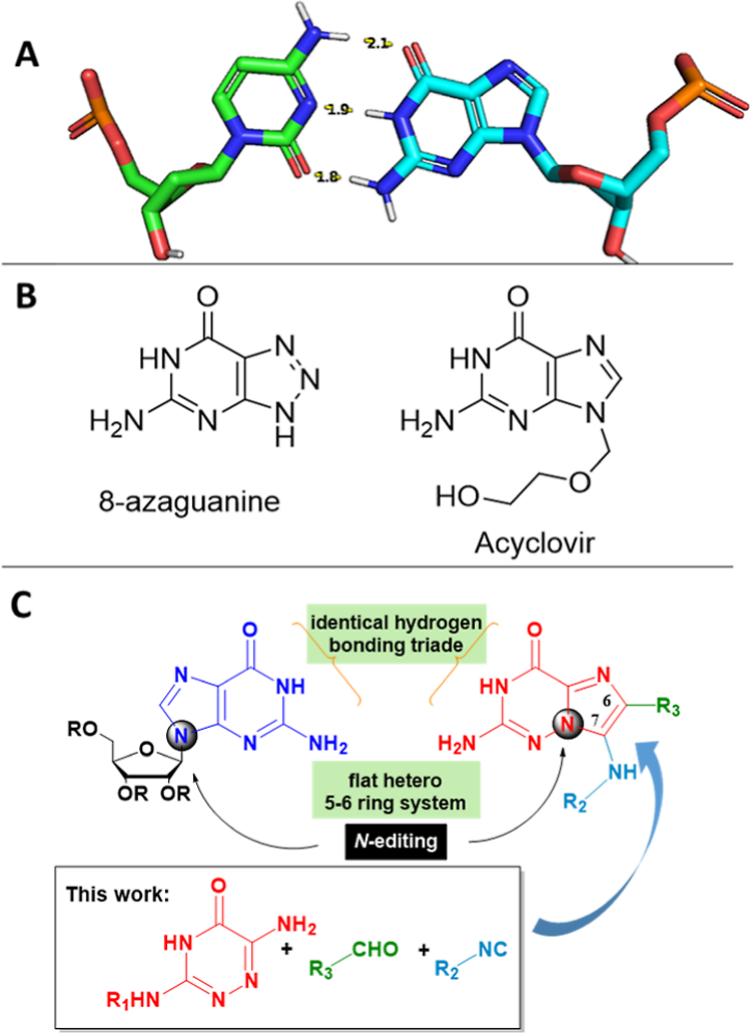
Nature of guanine. (A) Watson–Crick base pairing involving cytosine and guanine. (B) Guanine moiety-containing drugs. (C) Design of a guanine isosteric scaffold by N-editing and its multicomponent reaction synthesis.

Guanine (2-amino-1,9-dihydro-6*H*-purin-6-one) was first reported in 1844 by the German chemist Julius Bodo Unger and later structurally elucidated by Emil Fischer.^1^ Guanine is a purine derivative, consisting of a fused planar pyrimidine-imidazole ring system. As a substructure of guanosine, it plays an outstanding role in the propagation of genetic information in living organisms by RNA and DNA (Figure 1A).^2^ Moreover, guanine is a part of several cofactors, for example, cGMP or GDP. Numerous diseases, e.g., cancer and K-RAS, are associated with malfunctioning guanine-dependent proteins and/or guanine catabolism.^3^ Several approved drugs are based on guanine moieties such as the anti-herpes simplex acyclovir or the antineoplastic 8-azaguanine (Figure 1B).^4^ Guanine derivatives are typically synthetically accessed by a sequential multistep synthesis from heterocyclic guanine precursors.^5^ Thus, there is an urgent need for convergent short and diverse syntheses of novel guanine derivatives. Analysis of the guanine binding interaction in DNA, RNA, and proteins reveals that key pharmacophoric elements include a flat heterocyclic 5–6 ring system and a hydrogen-bonding triade HBA–HBD–HBD (HBA = hydrogen bond acceptor; HBD = hydrogen bond donor) of an acceptor carbonyl-O, an adjacent NH, and an exocyclic amino group (Figure 1C). Therefore, a guanine bioisostere should be composed of a flat heterocyclic ring system incorporating the essential HBA–HBD–HBD triade. Based on our interest in multicomponent reaction (MCR) chemistry, we reasoned that a generalized scaffold obeying the pharmacophore requirements of guanine would be accessible by an unprecedented atypical Groebke-Blackburn-Bienaymé reaction (GBB-3CR) of a heterocyclic amidine, an aldehyde, and an isocyanide (Figure 1C).^6^

In the spirit of the emerging research area ‘single-atom skeletal editing’, the imidazo-*N*-9 of the purine would shift into the next bridgehead 4-position. The resulting scaffold indeed would closely resemble guanine: the key hydrogen-bonding triade is identical, the scaffold consists of a flat hetero 5–6 ring system, and the chemistry would allow substitution at the 6 and 7 positions.

The 7-position corresponds to the (deoxy)ribose position of guanine and biologically relevant derivatives. Due to the logic of the herein used chemistry, a bridgehead N is shifted in the new scaffold, which corresponds to the neighboring 9-position in guanine. In principle, the new heterocyclic ring system could result in a differential distribution of tautomeric microspecies which is important for biological activity. Indeed, analyzing the tautomeric microspecies using the ChemAxon Tautomerizer in water at room temperature revealed that the major species is identical with the guanine major tautomer (Supporting Information).^7^ Interestingly, the major tautomer species is present over a broad pH range from 2.5 until 9. Taken together, our design and the predicted properties made us confident to investigate and optimize the GBB-3CR reaction to access a new class of guanine bioisosteres.

## Results and Discussion

**Scheme 1:**
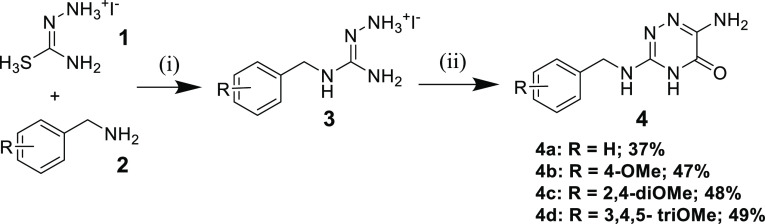
Synthesis of 1,2,4-Triazin-5(4*H*)-ones Reaction conditions: (i) 2-propanol, 40 °C, then r.t., 2 d; (ii) ethyl 2-amino-2-thioxoacetate, 75 °C, 2.5 h, then ice water, 16 h.

Here we reported the synthesis of novel 4-aza-9-deaza-guanine isosteres by a one-pot two-step protocol combining the GBB-3CR and an acid-assisted deprotection reaction, resulting in a library of diverse analogues bearing imidazo[2,1-*f*][1,2,4]triazin-4(3*H*)-one scaffold. First, we synthesized 1-amino-3-benzylguanidine hydroiodide (**3**) from hydrazinecarbothioamide hydroiodide (**1**) and benzylamine (**2**).^8^ The cyclization of **3** with ethyl 2-amino-2-thioxoacetate (**4**) provided the four building blocks 1,2,4-triazin-5-one **4a**–**4d** with different benzyl protecting groups in 37–49% yields (Scheme 1).

To optimize the reaction condition for the construction of the GBB intermediates, 6-amino-3-((3,4,5-trimethoxybenzyl)amino)-1,2,4-triazin-5-one (**4d**), benzaldehyde (**4e**), and cyclohexyl isocyanide (**4f**) were selected for a model reaction (Table 1). Initially, **4d**, **4e**, and **4f** were combined sequentially in methanol (0.5 M) at 100 °C at room temperature for 12 h in the presence of 0.2 equimolar Sc(OTf)_3_, which gave the GBB product **5l** in a moderate yield (45%, entry 1). Decreasing the amount of catalyst to 0.1 equimolar slightly improved the yield from 45 to 49%. Microwave irradiation promoted the reaction when heating the system at 100 °C for 2 h only with 0.1 equimolar Sc(OTf)_3_ (entry 4, 60%) rather than 0.2 equimolar Sc(OTf)_3_ (entry 3, 39%). Both shortening (entry 5, 51%) and prolonging (entry 6, 44%) the reaction time are detrimental to the reaction. Conventional heating (entry 7, 49%) offered no advantage in increasing the yield. Some other popular solvents for the GBB reactions were also screened in this work, but higher yields could not be obtained with polar solvents such as EtOH or nonpolar solvents such as toluene and acetonitrile. Catalyst variations such as La(OTf)_3_, Gd(OTf)_3_, HClO_4_, and AcOH failed to further improve the yield, only realizing lower yields from 20 to 38%.

**Table 1:**
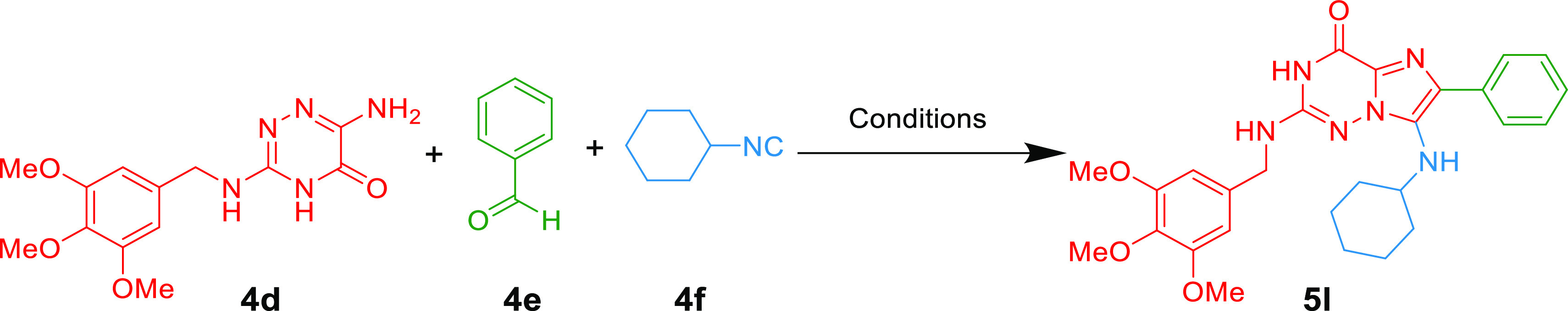
Optimization of the GBB-3CR Conditions^a^

entry	catalyst	solvent	temperature ( °C)	time	yields (%)
1	Sc(OTf)_3_(0.2)	MeOH	r.t.	12 h	45
2	Sc(OTf)_3_(0.1)	MeOH	r.t.	12 h	49
3	Sc(OTf)_3_(0.2)	MeOH	100	2 h^b^	39
4	Sc(OTf)_3_(0.1)	MeOH	100	2 h^b^	60
5	Sc(OTf)_3_(0.1)	MeOH	100	1 h^b^	51
6	Sc(OTf)_3_(0.1)	MeOH	100	4 h^b^	44
7	Sc(OTf)_3_(0.1)	MeOH	100	2 h^c^	49
8	Sc(OTf)_3_(0.1)	EtOH	100	2 h^b^	53
9	Sc(OTf)_3_(0.1)	toluene	100	2 h^b^	44
10	Sc(OTf)_3_(0.1)	MeCN	100	2 h^b^	28
11	La(OTf)_3_	MeOH	100	2 h^b^	38
12	Gd(OTf)_3_	MeOH	100	2 h^b^	35
13	HClO_4_(0.15)	MeOH	r.t.	12 h	37
14	HClO_4_(0.15)	MeOH	100	2 h^b^	34
15	AcOH(2)	MeOH	r.t.	12 h	20

a Reaction conditions: **4d** (0.5 mmol), **4e** (0.6 mmol), **4f** (0.6 mmol), catalyst (10 or 20 mmol %), and solvent (2 mL).

b Microwave condition.

c Conventional heating using aluminum heating blocks.

**Scheme 2:**
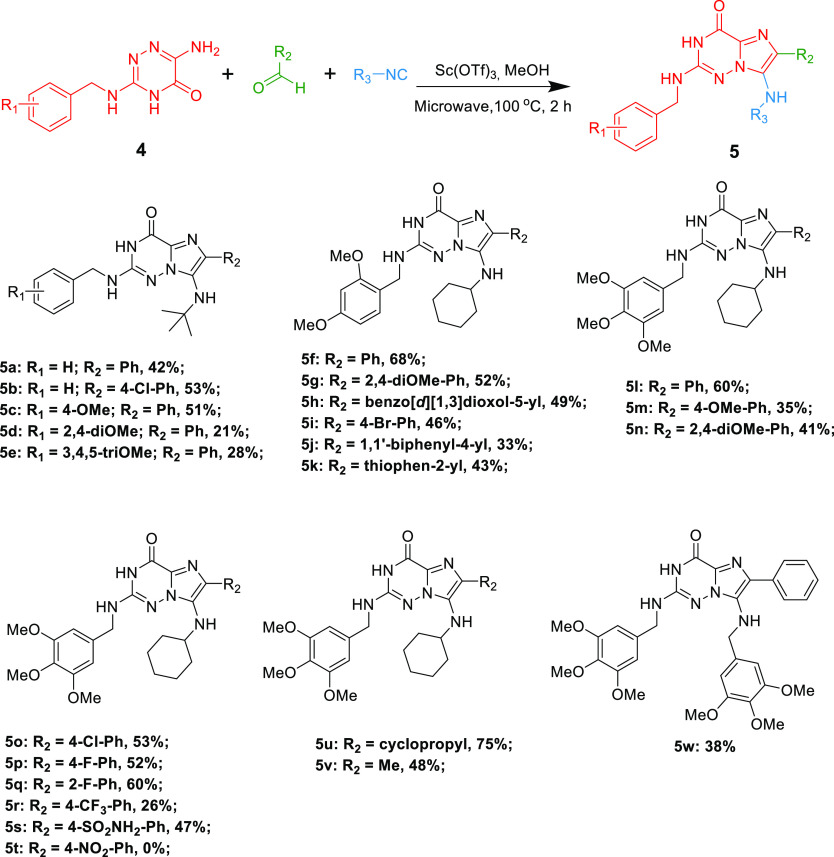
Variation of the 2-Amidines, Aldehydes, and Isocyanides

With optimal reaction conditions in hand, the scope of the 6-amino-1,2,4-triazin-5-ones, aldehydes, and isocyanides was explored (Scheme 2). *Tert*-butyl isocyanide and cyclohexyl isocyanide were first introduced in the reaction to exploit cleavability of R_3_ in the next step. With *tert*-butyl isocyanide, the relevant GBB products (**5a**–**5e**) could be achieved in 21–53% yields.

Employing a benzyl-protecting group (R_1_ = H) in the amidine **4**, the GBB compounds **5a** and **5b** could be obtained smoothly in moderate 42 and 50% yields, respectively. 4-chlorobenzaldehyde and benzaldehyde performed similarly (**5b** vs **5c**, 53 vs 51%). Introducing *para*-methoxy group in R_1_ yielded a moderate 51%. On replacing R_1_ with 2,4-dimethoxy (**5d**, 21%) or 3,4,5-trimethoxy (**5e**, 28%) group, the yields decreased. Running the reaction with cyclohexyl isocyanide, almost all GBB products could be realized successfully, except with 4-NO_2_-benzaldehyde (**5t**, 0%). With the 2,4-dimethoxy group in the R_1_ part, both electron-donating group and electron-withdrawing group in R_2_ are tolerated, achieving final products in medium yields, from 33 to 68%. Generally, electron-donating groups (**5f**–**5h**, **5j**) in R_2_ are more active than electron-withdrawing groups (**5i**, **5k**). When replacing 2,4-dimethoxy group with 3,4,5-trimethoxy group in R_1_, the GBB cyclization compounds could also be readily obtained with 26–75% yields. Overall, benzaldehydes with electron-withdrawing groups (**5o**–**5q** and **5s**, 47–60% yields) could achieve higher yields than electron-donating-group-substituted benzaldehydes (**5l**–**5n**, 35–60% yields), with two exceptions, 4-CF_3_-benzaldehyde (**5r**, 26%) and the aforementioned 4-NO_2_-benzaldehyde (**5s**, 0%). Interestingly, aliphatic aldehydes like cyclopropane carbaldehyde (**5u**, 75%) and acetaldehyde (**5v**, 48%) resulted also in the desired products in a medium to good yield. Moreover, the aromatic 3,4,5-trimethoxybenzyl isocyanide was well tolerated, providing the GBB intermediate **5w** in a 38% yield.

**Scheme 3:**
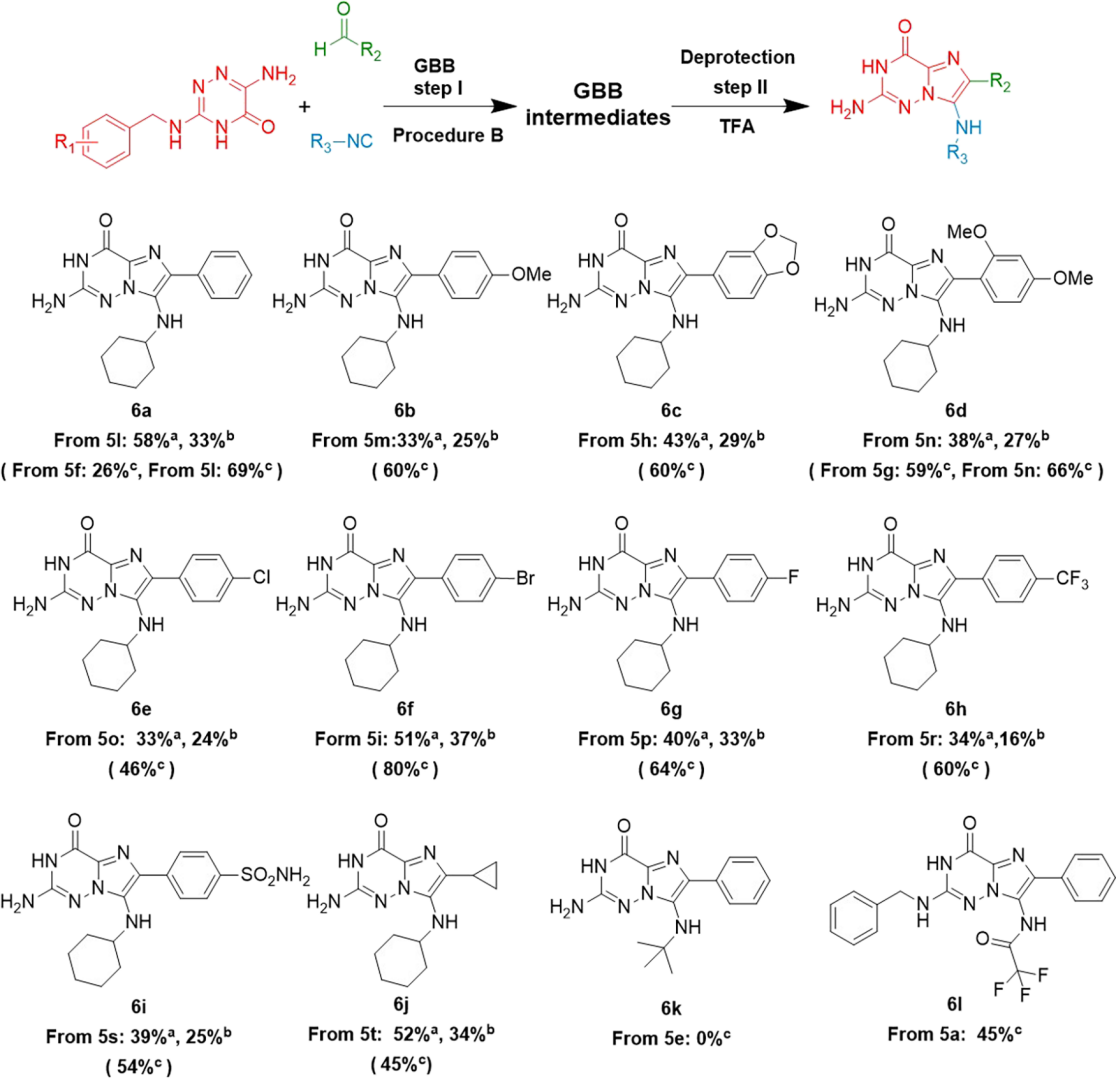
One-Pot Benzyl Deprotection with TFA Reaction conditions of Step II: Ugi reaction crude (0.2–0.5 mmol), 0.1 M TFA, 80 °C, 12 h, conventional heating; ^a^yields from the one-pot procedure without isolation of GBB intermediates; ^b^total yields calculated over the two-step procedure with isolated GBB intermediates; ^c^yields from only the deprotection step II with purified GBB intermediates.

**Scheme 4:**
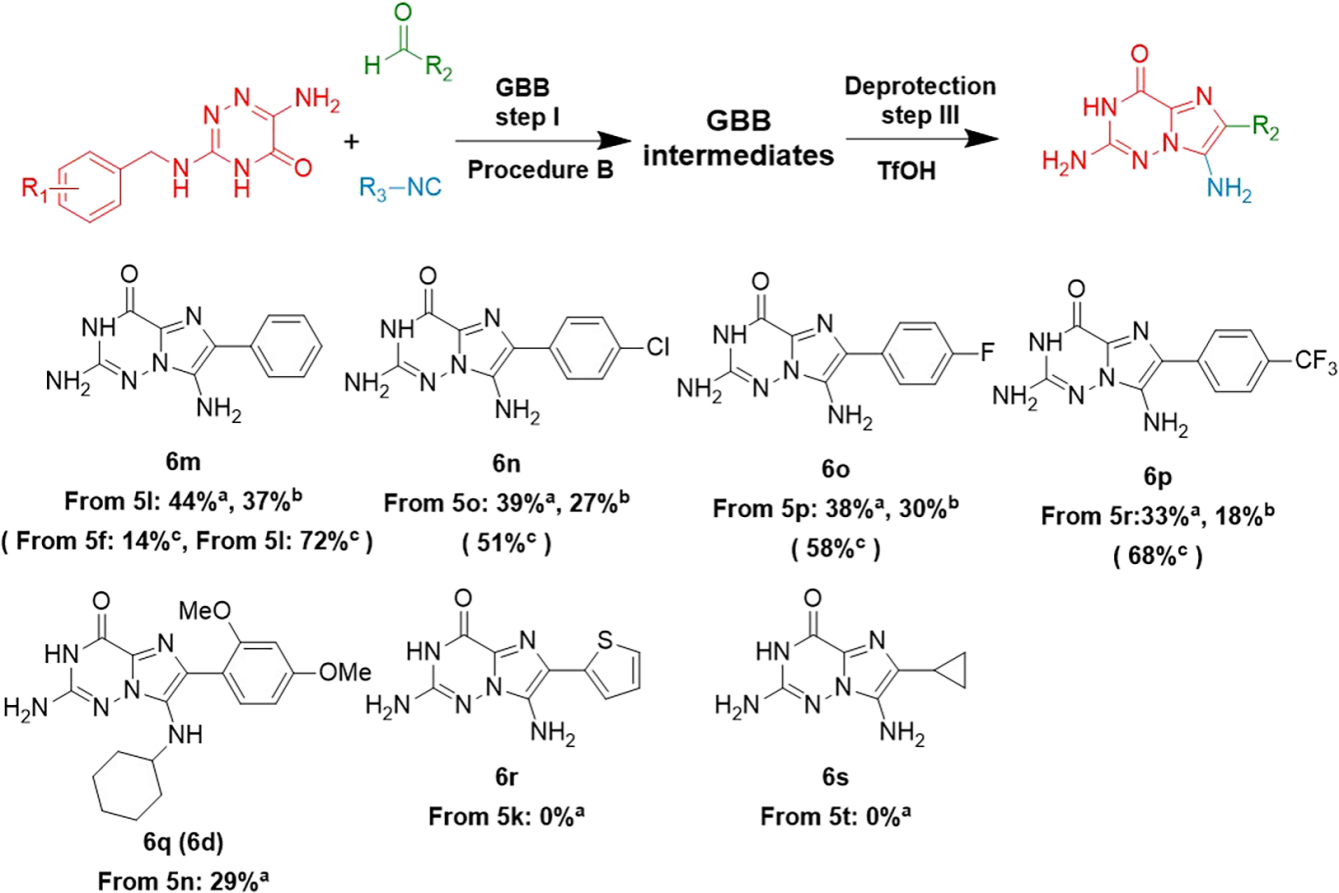
One-Pot Double Deprotection with TfOH Reaction conditions of Step III: Ugi reaction crude (0.1–0.5 mmol), 0.1 M TfOH, 55 °C, 4 h, conventional heating. ^a^yields from the one-pot procedure without isolation of GBB intermediates; ^b^total yields calculated over the two-step procedure with isolated GBB intermediates; ^c^yields from only the deprotection step III with purified GBB intermediates.

Next, in order to produce the target 4-aza-9-deaza-guanine isosteres, we needed to deprotect the benzyl groups in the previously obtained GBB-3CR intermediates in the following step. For this, we screened 20 deprotection conditions (Table S1, Supporting Information) and found that both trifluoroacetic acid (TFA)^9^ and trifluoromethansulfonic acid (TfOH)^10^ could cleave the benzyl groups, but the final deprotected products induced by those two acids are slightly different. While TFA can only deprotect the benzyl group (Scheme 3), the superacid TfOH cleaves simultaneously the benzyl group and the R_3_ group (Scheme 4).

In the TFA-assisted one-pot deprotection, we found that the deprotected products **6a**–**6j** formed well when R_3_ was a cyclohexyl group, achieving yields from 33 to 58%. GBB-3CR intermediates derived from electron-donating-groups- (**6a**–**6d**) and electron-withdrawing-groups- (**6e**–**6i**) substituted benzaldehydes both proceeded successfully in the one-pot benzyl deprotection step. It is noteworthy that the one-pot yields of all deprotected products **6a**–**6j** are on average 14% higher than those generated by the two separate steps procedure. Moreover, we also ran the deprotection reactions with all purified GBB-3CR intermediates and summarized the yields in Scheme 3. It is noteworthy that the yield of **6a** is much higher when generated from **5l** (69%) than **5f** (26%), and **5n** (66%) can obtain **6d** in a higher yield than **5g** (59%) as well. Those two examples suggest that the 3,4,5-trimethoxy group in R_1_ is easier to cleave than the 2,4-dimethoxy substituent. In addition, on replacing R_3_ with the *tert*-butyl group, the GBB-3CR intermediate **5e** failed to provide the target compound **6k**, while **5a** gave the unexpected trifluoroacetylation product **6l** in a 45% yield.

The TfOH-promoted one-pot double deprotection reactions worked well with the benzaldehydes-derived GBB intermediates, affording products **6m**–**6p** in 33–44% yields. The yields of **6m**–**6p** achieved by the one-pot method were found to be superior to those from the two separate steps procedure by an average of 11%. Surprisingly, the GBB product **5n** generated from 2,4-dimethoxy benzaldehyde provided the mono-deprotected compound **6q** (29%) rather than the double-deprotected product. The distinct yields difference from single-step deprotection in the synthesis of **6m** from **5f** (14%) or **5l** (72%) demonstrated that the 3,4,5-trimethoxy group in R_1_ is easier to deprotect TfOH-assisted, in accordance with its higher ring electron density. However, GBB-3CR intermediates constructed with thiophene-2-carbaldehyde (**5k**) and cyclopropane carbaldehyde (**5t**) did not afford the desired products **6r** or **6s**. This may be due to instability of the thiophene and cyclopropane rings in TfOH.

**Scheme 5:**
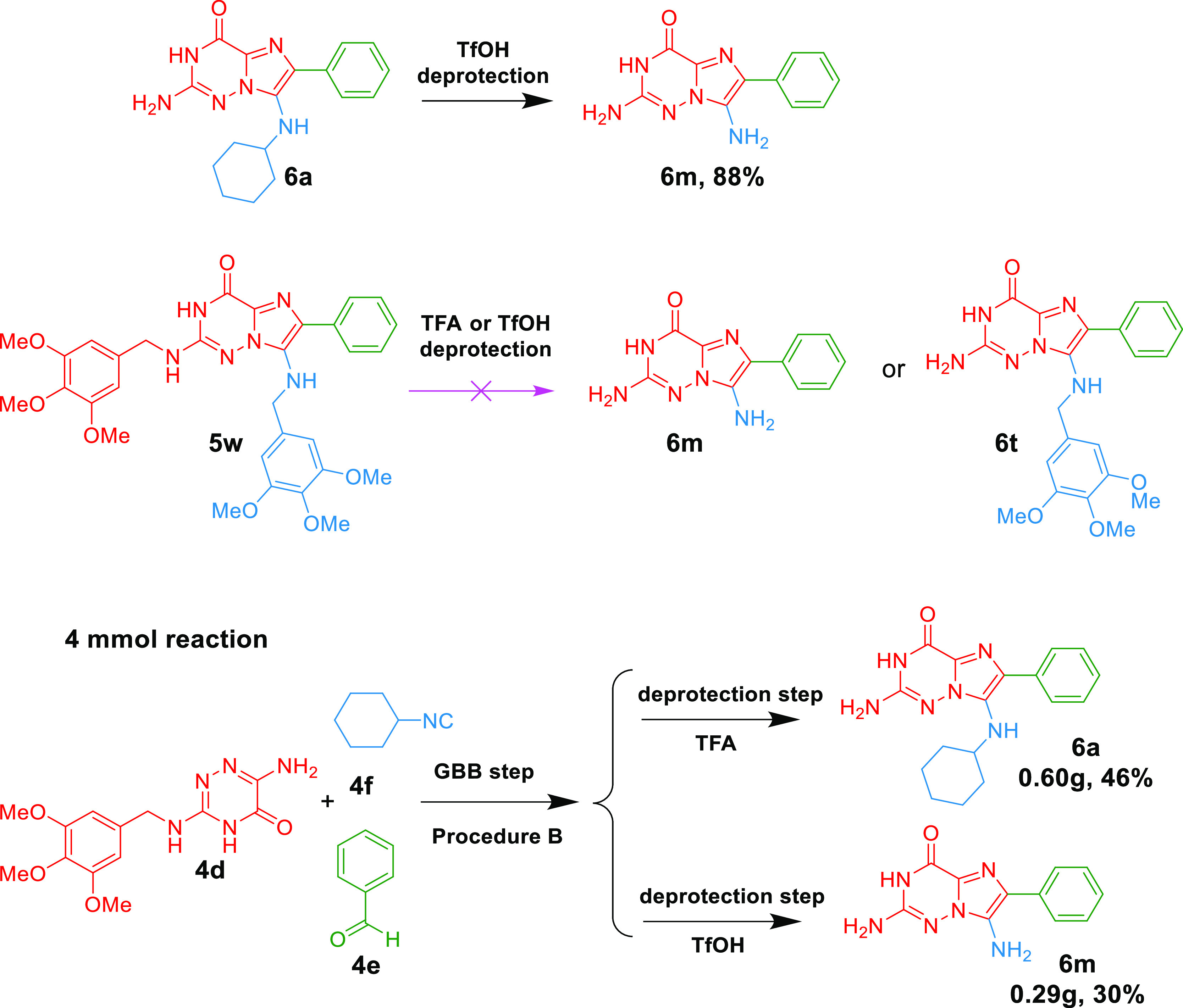
Control Experiment and Scale-Up Reaction

To further fortify the usefulness of our new synthesis, we carried out the control experiment and scale–up reaction (Scheme 5). It turned out that the mono-deprotected compound **6a** could be further deprotected in the presence of TfOH to provide **6m** in an 88% yield, which indicated that TfOH could not only achieve double deprotection of benzyl and cyclohexyl groups but also cleave the cyclohexyl alone. Our attempt to figure out whether TFA or TfOH could simultaneously cleave two benzyl groups failed; compound **5w** could not yield either double-deprotected **6m** or single-deprotected **6t**. The scale-up reactions of our one-pot procedures were performed on a 4 mmol scale, providing **6a** and **6m** in 46 and 30% yields, respectively. The D_2_O exchange NMR experiments of **6a** and **6m** were done to prove the mono- or double-deprotection (Figures S3 and S4, Supporting Information).

**Figure 2:**
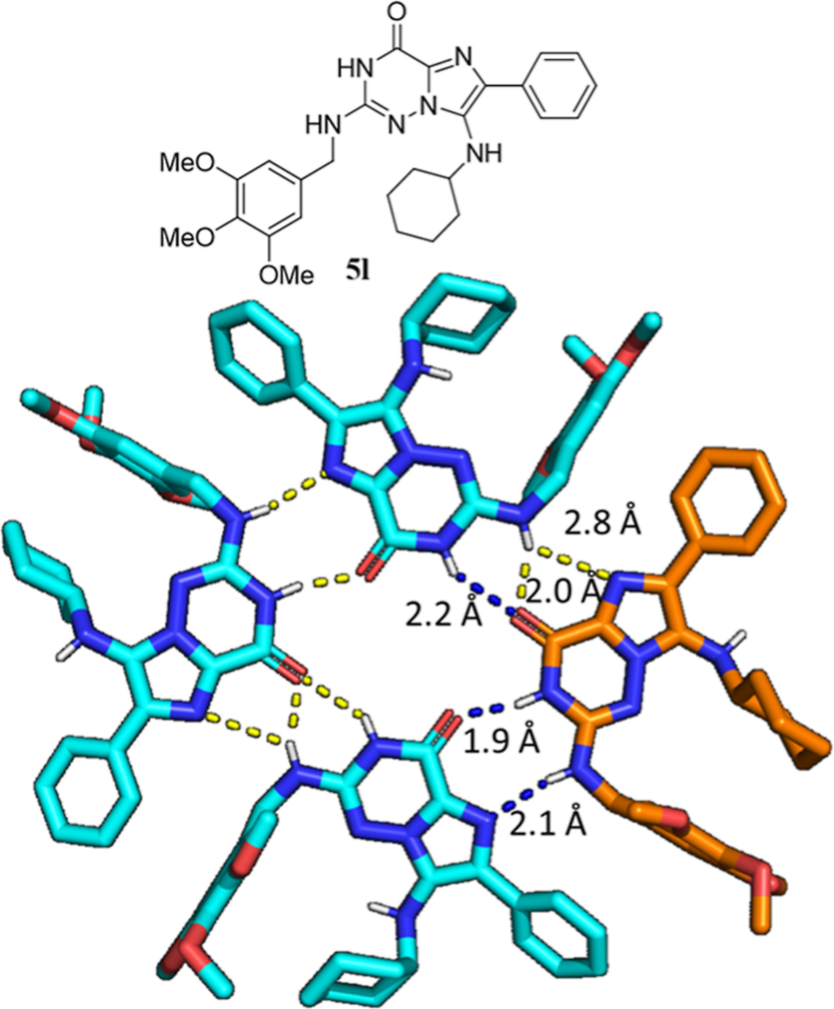
X-ray structure of G-analogue **5l** (CCDC 2190420) in solid state. 2D structure and 3D structure of the tetrameric macrocyclic assembly exhibiting a dense hydrogen-bonding network (dotted lines). For clarity, one molecule is shown in golden sticks, including the important trifurcated hydrogen-bonding pattern (blue dotted lines).

The X-ray crystal structures of **4d** (Supporting Information) and **5l** were obtained, demonstrating the solid-state structures of the scaffold. Interestingly, the G-analogue **5l** exhibits a trifurcated hydrogen-bonding pattern in a circular tetrameric macrocyclic conformation (Figure 2). This closely mimics the G-binding pattern found in all GTP/GDP-protein structures, suggesting that our heterocyclic G-mimic indeed could act as a bioisosteric G-mimic.

In summary, an innovative GBB-3CR-based one-pot two-step synthesis of novel 4-aza-9-deaza-guanine isosteres has been developed using a ‘single-atom skeletal editing’ strategy. Generally, most of our two-step syntheses are not high yielding, still they are superior to previous multistep synthesis protocols and will help to enrich the toolbox of guanine isosteres by an unprecedented new member scaffold. Combining GBB-3CR reaction and subsequent TFA- or TfOH-assisted deprotection reaction, mono-deprotected guanine isosteres and double deprotected guanine isosteres can be achieved, separately. Having the same hydrogen-binding pattern as guanine, our 9-deaza-guanine isosteres may also form similar interactions with biological receptors. Currently, biological evaluation of our G-analogues is ongoing and will be reported in due course.

## Experimental Section

### General Information

Reagents were available from commercial suppliers and used without any purification unless otherwise noted. All isocyanides were made in-house via the Ugi procedure.^11^ Other reagents were purchased from Sigma-Aldrich, ABCR, Acros, Fluorochem, AK Scientific, Combiblocks, or A2B and were used without further purification. Nuclear magnetic resonance spectra were recorded on a Bruker Avance 500 spectrometer. Chemical shifts for ^1^H NMR were reported relative to TMS (δ 0 ppm) or internal solvent peak (CDCl_3_ δ 7.26 ppm, CD_3_OD δ 3.31 ppm, or D_2_O δ 4.79 ppm), and coupling constants were in hertz (Hz). The following abbreviations were used for spin multiplicity: s = singlet, d = doublet, t = triplet, dt = double triplet, ddd = doublet of double doublet, m = multiplet, and br = broad. Chemical shifts for ^13^C NMR were reported in ppm relative to the solvent peak (CDCl_3_ δ 77.23 ppm, DMSO δ 39.52 ppm, and CD_3_OD δ 49.00 ppm). Flash chromatography was performed on a Grace Reveleris X2 system using Grace Reveleris Silica columns (12 g), and a gradient of petroleum ether/ethyl acetate (0–100%) or dichloromethane/methanol (0–20%) was applied. Thin layer chromatography was performed on Fluka precoated silica gel plates (0.20 mm thick, particle size 25 μm). Mass spectra were measured on a Waters Investigator Supercritical Fluid Chromatograph with a 3100 MS Detector (ESI) using a solvent system of methanol and CO_2_ on a Viridis silica gel column (4.6 × 250 mm, 5 μm particle size) and reported as (*m*/*z*). High-resolution mass spectra (HRMS) were recorded using an LTQ-Orbitrap-XL (Thermo Fisher Scientific; ESI pos. mode) at a resolution of 60,000@*m*/*z* 400. All microwave irradiation reactions were carried out in a Biotage Initiator microwave synthesizer. Melting points were obtained on a melting point apparatus and were uncorrected. The yields given refer to chromatographically purified compounds unless otherwise stated.

### General Experimental Procedure and Characterization

#### General Procedure A: Synthesis of 1,2,4-Triazin-5(4*H*)-ones (**4a–4d**)

Hydrazinecarbothioamide hydroiodide (20 mmol, 1.0 equiv) was suspended in 20 mL of 2-propanol (20 mL, 1.0 M), then benzylamine (21 mmol, 1.05 equiv) was added. The reaction was heated at 40 °C for 10 h, and then the reaction mixture was kept stirring at room temperature for 2 more days. The solid was filtered and the solvents were evaporated in vacuum. The remaining crude was recrystallized with DCM and diethyl ether to give white solid amino-3-benzylguanidine hydroiodide. To a mixture of 1-amino-3-benzylguanidine hydroiodide (5.0 mmol, 1.0 equiv) and K_2_CO_3_ (5.1 mmol, 1.02 equiv) in DMSO (10 mL, 0.5 M), ethyl-2-amino-2-thioxo-acetate (5.5 mmol, 1.1 equiv) was added and kept for 2.5 h at 75 °C in an oil bath. After heating, the gray mixture was poured under vigorous stirring into 70 mL of ice water and stirred for another 18 h to yield a yellow crystalline precipitate. The precipitate was filtration and washed with water (3 × 20 mL) and then ethylacetate/ether (1:1, 3 × 20 mL). The solid was recrystallized from methanol or purified by silica chromatography to give **4a**–**4d**.

#### General Procedure B

Corresponding 6-amino-3-(benzylamino)-1,2,4-triazin-5(4*H*)-one (0.2–1 mmol, 1.0 equiv) and aldehyde (0.24–1.4 mmol, 1.2 equiv) were dissolved in MeOH (0.8–4 mL, 0.25 M) in a microwave tube, the mixture was stirred at room temperature for 10 min, then isocyanide (0.24–1.4 mmol, 1.2 equiv) and Sc(OTf)_3_ (10 mmol %, 0.1 equiv) were added, and the tube was sealed. Then the mixture was heated at 100 °C under microwave in a sealed tube for 2 h. During the reaction, the temperature was monitored by the temperature–time profile on the screen of the microwave machine. After the reaction, the mixture was purified by silica gel column chromatography (MeOH/DCM = 1–5%) to give compounds **5a–5u**.

#### General Procedure C: Benzyl Deprotection with TFA

GBB intermediate (0.2–0.5 mmol) was dissolved in TFA (2–5 mL, 0.1 M). The reaction mixture was stirred at 80 °C overnight in a sealed tube. After reaction, the reaction mixture was diluted with 20 mL of DCM, and then the solvents were removed under reduced pressure. Then, the residue was diluted with EA (50 mL) and washed with sat·NaHCO_3_ (50 mL × 3). Then the organic layer was dried over MgSO_4_, filtered, and the solvent was removed under reduced pressure. Then the crude compound was purified by silica gel column chromatography (MeOH/DCM = 2–10%) to get the deprotected products **6a–6l**.

#### General Procedure D: Deprotection with TfOH

GBB intermediate (0.1–0.5 mmol) was treated with triflic acid (1–5 mL, 0.1 M), then heated at 55 °C for 4 h. After the reaction, the mixture was quenched with water and neutralized with sat.NaHCO_3_. The aqueous layer was extracted with EA and the combined organic layer was washed with brine, dried, and concentrated under vacuum. Then the crude compounds were purified by silica gel column chromatography (MeOH/DCM = 2–10%) to get the deprotected products **6m–6q**.

#### General Procedure E: One-Pot Synthesis

First, GBB reactions were carried out according to procedure B; after the reaction, the solvent was removed directly and the reaction mixture underwent in situ deprotection reaction following procedure C or D. Then the crude compounds were purified by silica gel column chromatography (MeOH/DCM = 2–10%) to get the deprotected products **6m–6q**.

##### 6-Amino-3-(benzylamino)-1,2,4-triazin-5(4*H*)-one (**4a**)

It was synthesized according to general procedure A on a 5 mmol scale and isolated using 1–3% MeOH/dichloromethane (v/v) to afford **4a** (406 mg, 37%) as a white solid. mp 219–221 °C. *R*_*f*_ = 0.46 (5% MeOH/dichloromethane). ^1^H NMR (500 MHz, DMSO): δ 11.05 (s, 1H), 7.28 (ddd, *J* = 26.5, 14.6, 7.4 Hz, 5H), 7.07–6.95 (m, 1H), 5.79 (s, 2H), and 4.38 (d, *J* = 6.3 Hz, 2H). ^13^C{^1^H} NMR (126 MHz, DMSO-*d*_6_): δ 159.5, 154.0, 146.7, 139.5, 128.3, 127.0, 126.8, and 43.0. HRMS (ESI) *m/z*: [M + H]^+^ calcd for C_10_H_12_ON_5_, 218.1036; found, 218.1036.

##### 6-Amino-3-((4-methoxybenzyl)amino)-1,2,4-triazin-5(4*H*)-one (**4b**)

It was synthesized according to general procedure A on a 5 mmol scale and isolated using 1–3% MeOH/dichloromethane (v/v) to afford **4b** (442 mg, 47%) as a light brown solid. mp 224–226 °C. *R*_*f*_ = 0.40 (5% MeOH/dichloromethane). ^1^H NMR (500 MHz, DMSO-*d*_6_): δ 11.01 (s, 1H), 7.22 (d, *J* = 8.2 Hz, 2H), 6.97 (s, 1H), 6.88 (d, *J* = 8.3 Hz, 2H), 5.78 (s, 2H), 4.30 (d, *J* = 5.9 Hz, 2H), and 3.72 (s, 3H). ^13^C{^1^H} NMR (126 MHz, DMSO-*d*_6_): δ 159.5, 158.3, 153.9, 146.6, 131.2, 129.0, 128.5, 113.7 (d, *J* = 15.8 Hz), 55.0, and 42.5. HRMS (ESI) *m/z*: [M + H]^+^ calcd for C_11_H_14_O_2_N_5_, 248.1142; found, 248.1142.

##### 6-Amino-3-((2,4-dimethoxybenzyl)amino)-1,2,4-triazin-5(4*H*)-one (**4c**)

It was synthesized according to general procedure A on a 5 mmol scale and isolated using 1–3% MeOH/dichloromethane (v/v) to afford **4c** (402 mg, 48%) as a yellow solid. mp 218–220 °C. *R*_*f*_ = 0.36 (5% MeOH/dichloromethane). ^1^H NMR (500 MHz, DMSO-*d*_6_): δ 10.90 (s, 1H), 7.07 (d, *J* = 8.4 Hz, 1H), 6.63 (s, 1H), 6.56 (s, 1H), 6.48 (d, *J* = 8.2 Hz, 1H), 5.77 (s, 2H), 4.25 (d, *J* = 6.0 Hz, 2H), 3.80 (s, 3H), and 3.73 (s, 3H). ^13^C{^1^H} NMR (126 MHz, DMSO-*d*_6_): δ 159.9, 159.5, 157.8, 154.1, 146.6, 128.6 (d, *J* = 11.3 Hz), 118.8, 104.2 (d, *J* = 19.9 Hz), 98.3 (d, *J* = 21.5 Hz), 55.4 (d, *J* = 16.6 Hz), 55.2 (d, *J* = 13.4 Hz), and 38.5. HRMS (ESI) *m/z*: [M + H]^+^ calcd for C_12_H_16_O_3_N_5_, 278.1248; found, 278.1247.

##### 6-Amino-3-((3,4,5-trimethoxybenzyl)amino)-1,2,4-triazin-5(4*H*)-one (**4d**)

It was synthesized according to general procedure A on a 3 mmol scale and isolated using 1–3% MeOH/dichloromethane (v/v) to afford (753 mg, 49%) as a yellow solid. mp 210–212 °C. *R*_*f*_ = 0.23 (5% MeOH/dichloromethane). ^1^H NMR (500 MHz, DMSO-*d*_6_): δ 11.03 (s, 1H), 6.99 (s, 1H), 6.64 (s, 2H), 5.80 (s, 2H), 4.30 (d, *J* = 6.3 Hz, 2H), 3.74 (s, 6H), and 3.62 (s, 3H). ^13^C{^1^H} NMR (126 MHz, DMSO-*d*_6_): δ 159.9, 154.4, 153.3, 147.2, 136.8, 135.4, 104.6 (d, *J* = 22.2 Hz), 60.5, 56.3 (d, *J* = 16.0 Hz), and 43.8. HRMS (ESI) *m/z*: [M + H]^+^ calcd C_13_H_18_O_4_N_5_, 308.1353; found, 308.1353.

##### 2-(Benzylamino)-7-(*tert*-butylamino)-6-phenylimidazo[2,1-*f*][1,2,4]triazin-4(3*H*)-one (**5a**)

It was synthesized according to general procedure B on a 0.375 mmol scale and isolated using 3–5% MeOH/dichloromethane (v/v) to afford **5a** (61 mg, 42%) as a white solid. mp 246–248 °C. *R*_*f*_ = 0.23 (3% MeOH/dichloromethane). ^1^H NMR (500 MHz, DMSO-*d*_6_): δ 11.18 (s, 1H), 8.09 (d, *J* = 7.7 Hz, 2H), 7.36 (dt, *J* = 24.4, 7.6 Hz, 6H), 7.24 (dt, *J* = 15.0, 7.4 Hz, 2H), 6.56 (t, *J* = 5.8 Hz, 1H), 4.47 (d, *J* = 6.1 Hz, 2H), 3.89 (s, 1H), and 0.96 (s, 9H). ^13^C{^1^H} NMR (126 MHz, DMSO-*d*_6_): δ 152.2, 148.2, 139.0, 135.0, 134.7, 129.8, 128.3, 127.9, 127.3, 126.9, 126.7, 126.6, 126.5, 55.9, and 30.1. HRMS (ESI) *m/z*: [M + H]^+^ calcd for C_22_H_25_ON_6_, 389.2084; found, 389.2083.

##### 2-(Benzylamino)-7-(*tert*-butylamino)-6-(4-chlorophenyl)imidazo[2,1-*f*][1,2,4]triazin-4(3*H*)-one (**5b**)

It was synthesized according to general procedure B on a 0.25 mmol scale and isolated using 3–5% MeOH/dichloromethane (v/v) to afford **5b** (55 mg, 53%) as a white solid. mp 258–260 °C. *R*_*f*_ = 0.32 (3% MeOH/dichloromethane). ^1^H NMR (500 MHz, DMSO-*d*_6_): δ 11.27 (s, 1H), 8.11 (d, *J* = 8.2 Hz, 2H), 7.48–7.17 (m, 7H), 6.61 (s, 1H), 4.47 (s, 2H), 3.94 (s, 1H), and 0.96 (s, 9H). ^13^C{^1^H} NMR (126 MHz, DMSO-*d*_6_): δ 152.8, 148.8, 139.5, 134.4, 134.0, 131.5, 130.4, 128.8, 128.5, 128.4, 127.8, 127.4, 127.3, 56.5, 44.7, and 30.5 (d, *J* = 8.3 Hz). HRMS (ESI) *m/z*: [M + H]^+^ calcd for C_22_H_24_ON_6_Cl, 423.1695; found, 423.1692.

##### 7-(*tert*-Butylamino)-2-((4-methoxybenzyl)amino)-6-phenylimidazo[2,1-*f*][1,2,4]triazin-4(3*H*)-one (**5c**)

It was synthesized according to general procedure B on a 1 mmol scale and isolated using 3–5% MeOH/dichloromethane (v/v) to afford **5c** (213 mg, 51%) as a white solid. mp 246–248 °C. *R*_*f*_ = 0.82 (5% MeOH/dichloromethane). ^1^H NMR (500 MHz, DMSO-*d*_6_): δ 11.24 (s, 1H), 8.11 (d, *J* = 7.2 Hz, 2H), 7.42–7.30 (m, 4H), 7.23 (t, *J* = 7.4 Hz, 1H), 6.91 (d, *J* = 8.6 Hz, 2H), 6.58 (s, 1H), 4.40 (d, *J* = 5.7 Hz, 2H), 3.93 (s, 1H), 3.74 (s, 3H), and 1.01 (s, 9H). ^13^C{^1^H} NMR (126 MHz, DMSO-*d*_6_): δ 158.4, 152.6, 148.4, 135.1, 134.7, 130.9, 129.7, 128.8, 127.9, 126.7, 126.5, 126.4, 113.7, 56.0, 55.1 (d, *J* = 12.4 Hz), 43.8, and 30.2. HRMS (ESI) *m/z*: [M + H]^+^ calcd for C_23_H_27_O_2_N_6_, 419.2190; found, 419.2187.

##### 7-(*tert*-Butylamino)-6-phenyl-2-((2,4-dimethoxybenzyl)amino)imidazo[2,1-*f*][1,2,4]triazin-4(3*H*)-one (**5d**)

It was synthesized according to general procedure B on a 1 mmol scale and isolated using 3–5% MeOH/dichloromethane (v/v) to afford **5d** (94 mg, 21%) as a yellow solid. mp 236–238 °C. *R*_*f*_ = 0.50 (5% MeOH/dichloromethane). ^1^H NMR (500 MHz, DMSO-*d*_6_): δ 1H NMR (500 MHz, DMSO-*d*_6_): δ 11.00 (s, 1H), 8.13–8.08 (m, 2H), 7.36 (t, *J* = 7.8 Hz, 2H), 7.26–7.19 (m, 2H), 6.59 (d, *J* = 2.4 Hz, 1H), 6.48 (dd, *J* = 8.3, 2.4 Hz, 1H), 6.25 (t, *J* = 5.7 Hz, 1H), 4.36 (s, 2H), 3.98 (s, 1H), 3.82 (s, 3H), 3.74 (s, 3H), and 1.03 (s, 9H). ^13^C{^1^H} NMR (126 MHz, DMSO-*d*_6_): δ 160.1, 158.1, 152.2, 148.2, 135.1, 134.8, 129.9, 129.7, 127.9, 126.7, 126.6, 126.5 (d, *J* = 4.3 Hz), 118.3, 104.3 (d, *J* = 20.2 Hz), 98.4 (d, *J* = 21.1 Hz), 56.1, 56.0, 55.5 (d, *J* = 17.4 Hz), 55.2 (d, *J* = 13.7 Hz), and 30.2. HRMS (ESI) *m/z*: [M + H]^+^ calcd for C_24_H_29_O_3_N_6_, 449.2296; found, 449.2294.

##### 7-(*tert*-Butylamino)-6-phenyl-2-((3,4,5-trimethoxybenzyl)amino)imidazo[2,1-*f*][1,2,4]triazin-4(3*H*)-one (**5e**)

It was synthesized according to general procedure B on a 1 mmol scale and isolated using 3–5% MeOH/dichloromethane (v/v) to afford **5e** (133 mg, 28%) as a light yellow solid. mp 231–233 °C. *R*_*f*_ = 0.37 (5% MeOH/dichloromethane). ^1^H NMR (500 MHz, DMSO-*d*_6_): δ 11.15 (s, 1H), 8.09 (dd, *J* = 8.2, 1.1 Hz, 2H), 7.36 (t, *J* = 7.8 Hz, 2H), 7.22 (t, *J* = 7.4 Hz, 1H), 6.70 (s, 2H), 6.52 (t, *J* = 5.6 Hz, 1H), 4.39 (d, *J* = 5.7 Hz, 2H), 3.92 (s, 1H), 3.76 (s, 6H), 3.62 (s, 3H), and 0.97 (s, 9H). ^13^C{^1^H} NMR (126 MHz, DMSO-*d*_6_): δ 152.9, 152.2, 148.1, 136.4, 135.0, 134.8, 134.7, 129.8, 127.9, 126.7, 126.5 (d, *J* = 4.8 Hz), 104.6, 104.5, 60.0, 56.0, 55.9 (d, *J* = 18.1 Hz), 44.6, and 30.1. HRMS (ESI) *m/z*: [M + H]^+^ calcd for C_25_H_31_O_4_N_6_, 479.2401; found, 479.2399.

##### 7-(Cyclohexylamino)-2-((2,4-dimethoxybenzyl)amino)-6-phenylimidazo[2,1-*f*][1,2,4]triazin-4(3*H*)-one (**5f**)

It was synthesized according to general procedure B on a 1 mmol scale and isolated using 3–5% MeOH/dichloromethane (v/v) to afford **5f** (322 mg, 68%) as a light yellow solid. mp 190–192 °C. *R*_*f*_ = 0.35 (5% MeOH/dichloromethane). ^1^H NMR (500 MHz, DMSO-*d*_6_): δ 10.91 (s, 1H), 7.99–7.91 (m, 2H), 7.39 (t, *J* = 7.8 Hz, 2H), 7.26 (d, *J* = 8.4 Hz, 1H), 7.23 (d, *J* = 7.4 Hz, 1H), 6.59 (d, *J* = 2.4 Hz, 1H), 6.47 (dd, *J* = 2.4, 8.4 Hz, 1H), 6.26 (s, 1H), 4.38–4.35 (m, 2H), 4.34 (s, 1H), 3.84 (s, 3H), 3.74 (s, 3H), 3.13–3.02 (m, 1H), 1.71 (d, *J* = 10.4 Hz, 2H), 1.64–1.58 (m, 2H), 1.46 (d, *J* = 9.5 Hz, 1H), 1.21 (d, *J* = 11.7 Hz, 2H), and 1.12–0.99 (m, 3H). ^13^C{^1^H} NMR (126 MHz, DMSO-*d*_6_): δ 170.4, 160.0, 158.1, 151.9, 148.2, 134.6, 131.9, 129.6, 129.3, 128.4, 126.4, 125.6, 125.4, 118.3, 98.4 (d, *J* = 21.5 Hz), 59.8, 55.6, 55.2 (d, *J* = 13.3 Hz), 33.5, 24.4, 20.8 (d, *J* = 11.9 Hz), and 14.1 (d, *J* = 13.3 Hz). HRMS (ESI) *m/z*: [M + H]^+^ calcd for C_26_H_31_O_3_N_6_, 475.2452; found, 475.2451.

##### 7-(Cyclohexylamino)-2-((2,4-dimethoxybenzyl)amino)-6-(2,4-dimethoxyphenyl)imidazo[2,1-*f*][1,2,4]triazin-4(3H)-one (**5g**)

It was synthesized according to general procedure B on a 1 mmol scale and isolated using 3–5% MeOH/dichloromethane (v/v) to afford **5g** (277 mg, 52%) as a yellow solid. mp 176–178 °C. *R*_*f*_ = 0.38 (5% MeOH/dichloromethane). ^1^H NMR (500 MHz, DMSO-*d*_6_): δ 10.70 (s, 1H), 7.36 (d, *J* = 8.4 Hz, 1H), 7.21 (d, *J* = 8.4 Hz, 1H), 6.64–6.58 (m, 3H), 6.48 (dd, *J* = 2.4, 8.4 Hz, 1H), 6.15 (t, *J* = 5.9 Hz, 1H), 4.31 (d, *J* = 5.8 Hz, 2H), 4.26 (d, *J* = 9.9 Hz, 1H), 3.83 (s, 3H), 3.80 (s, 3H), 3.79 (s, 3H), 3.74 (s, 3H), 3.28–3.21 (m, 1H), 1.64 (q, *J* = 4.6 Hz, 2H), 1.51 (s, 2H), 1.41 (d, *J* = 11.7 Hz, 1H), and 1.00 (t, *J* = 9.8 Hz, 5H). ^13^C {^1^H} NMR (126 MHz, DMSO-*d*_6_): δ 160.1, 160.0, 158.0, 156.9, 151.6, 147.8, 132.6, 131.5, 129.2, 125.3, 124.4, 118.4, 116.4, 105.5, 105.3, 98.5, 98.3, 55.5, 55.3, 55.2, 55.1, 52.2, 33.4, 25.3, 24.3, and 24.1. HRMS (ESI) *m/z*: [M + H]^+^ calcd for C_28_H_35_O_5_N_6_, 535.2663; found, 535.2661.

##### 6-(Benzo[d][1,3]dioxol-5-yl)-7-(cyclohexylamino)-2-((2,4-dimethoxybenzyl)amino)imidazo[2,1-*f*][1,2,4]triazin-4(3*H*)-one (**5h**)

It was synthesized according to general procedure B on a 1 mmol scale and isolated using 3–5% MeOH/dichloromethane (v/v) to afford **5h** (253 mg, 49%) as a yellow solid. mp 183–185 °C. *R*_*f*_ = 0.44 (5% MeOH/dichloromethane). ^1^H NMR (500 MHz, DMSO-*d*_6_): δ 10.88 (s, 1H), 7.50–7.45 (m, 2H), 7.25 (d, *J* = 8.4 Hz, 1H), 6.95 (d, *J* = 8.0 Hz, 1H), 6.59 (d, *J* = 2.4 Hz, 1H), 6.46 (dd, *J* = 2.4, 8.3 Hz, 1H), 6.24 (t, *J* = 5.8 Hz, 1H), 6.02 (s, 2H), 4.33 (d, *J* = 6.0 Hz, 2H), 4.30 (d, *J* = 8.8 Hz, 1H), 3.83 (s, 3H), 3.74 (s, 3H), 3.05 (d, *J* = 9.0 Hz, 1H), 1.70 (d, *J* = 11.5 Hz, 2H), 1.62 (d, *J* = 12.0 Hz, 2H), 1.46 (s, 1H), 1.21 (d, *J* = 22.5 Hz, 3H), and 1.09 (d, *J* = 7.7 Hz, 2H). ^13^C{^1^H} NMR (126 MHz, DMSO-*d*_6_): δ 160.0, 158.1, 151.8, 148.2, 147.3, 145.8, 131.1, 129.6 (d, *J* = 14.2 Hz), 128.8, 125.1, 119.1 (d, *J* = 19.2 Hz), 118.3, 108.3 (d, *J* = 12.8 Hz), 105.9 (d, *J* = 19.7 Hz), 104.2 (d, *J* = 24.7 Hz), 100.8, 98.3, 80.5, 55.5 (d, *J* = 20.2 Hz), 55.3, 55.1, 33.5, 28.3, 25.3, and 24.3 (d, *J* = 23.4 Hz). HRMS (ESI) *m/z*: [M + H]^+^ calcd for C_27_H_31_O_5_N_6_, 519.235; found, 519.2347.

##### 6-(4-Bromophenyl)-7-(cyclohexylamino)-2-((2,4-dimethoxybenzyl)amino)imidazo[2,1-*f*][1,2,4]triazin-4(3*H*)-one (**5i**)

It was synthesized according to general procedure B on a 0.5 mmol scale and isolated using 3–5% MeOH/dichloromethane (v/v) to afford **5i** (127 mg, 46%) as a yellow solid. mp 180–183 °C. *R*_*f*_ = 0.46 (5% MeOH/dichloromethane). ^1^H NMR (500 MHz, DMSO-*d*_6_): δ 10.97 (s, 1H), 7.91 (d, *J* = 8.7 Hz, 2H), 7.58 (d, *J* = 8.7 Hz, 2H), 7.26 (d, *J* = 8.4 Hz, 1H), 6.59 (d, *J* = 2.4 Hz, 1H), 6.46 (dd, *J* = 2.5, 8.4 Hz, 1H), 6.28 (t, *J* = 6.1 Hz, 1H), 4.43 (d, *J* = 8.8 Hz, 1H), 4.34 (d, *J* = 5.8 Hz, 2H), 3.83 (s, 3H), 3.73 (s, 3H), 3.03 (d, *J* = 9.0 Hz, 1H), 1.70 (d, *J* = 10.1 Hz, 2H), 1.62 (s, 2H), 1.46 (s, 1H), 1.21 (d, *J* = 12.8 Hz, 3H), and 1.10–1.06 (m, 2H). ^13^C{^1^H} NMR (126 MHz, DMSO-*d*_6_): δ 160.0, 158.1, 151.9, 148.2, 133.8, 132.1, 131.4, 131.2, 129.6, 128.5, 127.4, 127.3, 125.7, 119.2, 118.3, 98.3, 68.5, 55.8, 55.6, 55.3, 33.5, 29.6, and 24.5. HRMS (ESI) *m/z*: [M + H]^+^ calcd for C_26_H_30_O_3_N_6_Br, 553.1557; found, 553.1555.

##### 6-([1,1′-Biphenyl]-4-yl)-7-(cyclohexylamino)-2-((2,4-dimethoxybenzyl)amino)imidazo[2,1-*f*][1,2,4]triazin-4(3*H*)-one (**5j**)

It was synthesized according to general procedure B on a 0.5 mmol scale and isolated using 3–5% MeOH/dichloromethane (v/v) to afford **5j** (91 mg, 33%) as a yellow solid. mp 169–171 °C. *R*_*f*_ = 0.48 (5% MeOH/dichloromethane). ^1^H NMR (500 MHz, DMSO-*d*_6_): δ 11.00 (s, 1H), 8.06 (d, *J* = 8.5 Hz, 2H), 7.75–7.70 (m, 4H), 7.46 (t, *J* = 7.8 Hz, 2H), 7.35 (t, *J* = 7.3 Hz, 1H), 7.27 (d, *J* = 8.4 Hz, 1H), 6.60 (d, *J* = 2.4 Hz, 1H), 6.47 (dd, *J* = 2.4, 8.4 Hz, 1H), 6.31 (t, *J* = 6.0 Hz, 1H), 4.44 (d, *J* = 9.0 Hz, 1H), 4.35 (d, *J* = 5.8 Hz, 2H), 3.84 (s, 3H), 3.74 (s, 3H), 3.16–3.06 (m, 1H), 1.74 (d, *J* = 11.5 Hz, 2H), 1.65–1.59 (m, 2H), 1.47 (s, 1H), 1.24 (d, *J* = 14.8 Hz, 3H), and 1.09 (d, *J* = 4.1 Hz, 2H). ^13^C{^1^H} NMR (126 MHz, DMSO-*d*_6_): δ 160.0, 158.1, 151.9, 148.2, 139.8, 137.8, 133.7, 132.1, 129.6, 129.1, 126.6, 126.5, 126.4, 126.3, 125.9, 125.6, 118.3, 104.1, 98.4 (d, *J* = 21.1 Hz), 68.5, 65.0, 55.8, 55.3, 33.6, 29.6, and 24.4. HRMS (ESI) *m/z*: [M + H]^+^ calcd for C_32_H_35_O_3_N_6_, 551.2765; found, 551.2762.

##### 7-(Cyclohexylamino)-2-((2,4-dimethoxybenzyl)amino)-6-(thiophen-2-yl)imidazo[2,1-*f*][1,2,4]triazin-4(3*H*)-one (**5k**)

It was synthesized according to general procedure B on a 0.5 mmol scale and isolated using 3–5% MeOH/dichloromethane (v/v) to afford **5k** (103 mg, 43%) as a white solid. mp 186–189 °C. *R*_*f*_ = 0.41 (5% MeOH/dichloromethane). ^1^H NMR (500 MHz, DMSO-*d*_6_): δ 10.97 (s, 1H), 7.41 (ddd, *J* = 1.3, 4.4, 9.3 Hz, 2H), 7.24 (d, *J* = 8.4 Hz, 1H), 7.08 (dd, *J* = 3.6, 5.0 Hz, 1H), 6.59 (d, *J* = 2.4 Hz, 1H), 6.46 (dd, *J* = 2.4, 8.3 Hz, 1H), 6.29 (t, *J* = 5.4 Hz, 1H), 4.42 (d, *J* = 8.7 Hz, 1H), 4.33 (d, *J* = 5.8 Hz, 2H), 3.83 (s, 3H), 3.73 (s, 3H), 3.25–3.17 (m, 1H), 1.75 (d, *J* = 12.1 Hz, 2H), 1.64 (s, 2H), 1.49 (s, 1H), 1.29–1.20 (m, 3H), and 1.12–1.09 (m, 2H). ^13^C{^1^H} NMR (126 MHz, DMSO-*d*_6_): δ 160.0, 158.1, 151.7, 148.2, 137.8, 130.6, 129.5, 127.7 (d, *J* = 5.5 Hz), 126.6, 124.0 (d, *J* = 14.7 Hz), 122.2 (d, *J* = 15.6 Hz), 118.3, 104.2 (d, *J* = 28.9 Hz), 98.4 (d, *J* = 25.2 Hz), 68.5, 55.8, 55.2 (d, *J* = 15.6 Hz), 54.8, 33.7, 29.6 (d, *J* = 9.6 Hz), and 24.5. HRMS (ESI) *m/z*: [M + H]^+^ calcd for C_24_H_29_O_3_N_6_S, 481.2016; found, 481.2015.

##### 7-(Cyclohexylamino)-6-phenyl-2-((3,4,5-trimethoxybenzyl)amino)imidazo[2,1-*f*][1,2,4]triazin-4(3*H*)-one (**5l**)

It was synthesized according to general procedure B on a 1 mmol scale and isolated using 3–5% MeOH/dichloromethane (v/v) to afford **5l** (302 mg, 60%) as a white solid. mp 224–226 °C. *R*_*f*_ = 0.43 (5% MeOH/dichloromethane). ^1^H NMR (500 MHz, DMSO-*d*_6_): δ 11.09 (s, 1H), 7.93 (dd, *J* = 8.3, 1.3 Hz, 2H), 7.39 (t, *J* = 7.9 Hz, 2H), 7.32–7.16 (m, 1H), 6.72 (s, 2H), 6.55 (s, 1H), 4.39 (d, *J* = 5.8 Hz, 2H), 4.33 (d, *J* = 9.3 Hz, 1H), 3.77 (s, 6H), 3.62 (s, 3H), 3.07–2.99 (m, 1H), 1.68 (d, *J* = 10.2 Hz, 2H), 1.56 (d, *J* = 13.1 Hz, 2H), 1.41 (d, *J* = 9.3 Hz, 1H), 1.15 (q, *J* = 11.3, 10.4 Hz, 2H), and 1.09–0.94 (m, 3H). ^13^C{^1^H} NMR (126 MHz, DMSO-*d*_6_): δ 152.9, 152.1, 148.2, 136.4, 134.8, 134.5, 131.9, 129.1, 128.3, 126.4, 125.5, 125.4, 104.5, 59.9, 56.4, 55.9, 55.8, 55.3, 55.1, 44.5, 33.4, 25.2, and 24.5. HRMS (ESI) *m/z*: [M + H]^+^ calcd for C_27_H_33_O_4_N_6_, 505.2558; found, 505.2557.

##### 7-(Cyclohexylamino)-6-(4-methoxyphenyl)-2-((3,4,5-trimethoxybenzyl)amino)imidazo[2,1-*f*][1,2,4]triazin-4(3*H*)-one (**5m**)

It was synthesized according to general procedure B on a 1 mmol scale and isolated using 3–5% MeOH/dichloromethane (v/v) to afford **5m** (187 mg, 35%) as white solid. mp 239–241 °C. *R*_*f*_ = 0.76 (5% MeOH/dichloromethane). ^1^H NMR (500 MHz, DMSO-*d*_6_): δ 11.03 (s, 1H), 7.87 (d, *J* = 8.8 Hz, 2H), 6.97 (d, *J* = 8.8 Hz, 2H), 6.72 (s, 2H), 6.53 (s, 1H), 4.38 (d, *J* = 6.0 Hz, 2H), 4.23 (d, *J* = 9.1 Hz, 1H), 3.77 (s, 9H), 3.62 (s, 3H), 3.01 (d, *J* = 9.3 Hz, 1H), 1.73–1.62 (m, 2H), 1.56 (d, *J* = 8.5 Hz, 2H), 1.42 (d, *J* = 8.7 Hz, 1H), 1.14 (q, *J* = 8.4, 9.9 Hz, 2H), and 1.07–0.96 (m, 3H). ^13^C{^1^H} NMR (126 MHz, DMSO-*d*_6_): δ 157.9, 152.9, 151.9, 148.2, 136.4, 134.8, 131.0, 129.6, 127.1, 126.7, 125.1, 113.8, 104.5, 60.0, 56.4, 55.8 (d, *J* = 18.3 Hz), 55.4 (d, *J* = 31.2 Hz), 55.0 (d, *J* = 16.5 Hz), 44.5, 33.6, and 24.2. HRMS (ESI) *m/z*: [M + H]^+^ calcd for C_28_H_35_O_5_N_6_, 535.2663; found, 535.2658.

##### 7-(Cyclohexylamino)-6-(2,4-dimethoxyphenyl)-2-((3,4,5-trimethoxybenzyl)amino)imidazo[2,1-*f*][1,2,4]triazin-4(3*H*)-one (**5n**)

It was synthesized according to general procedure B on a 0.5 mmol scale and isolated using 3–5% MeOH/dichloromethane (v/v) to afford **5n** (116 mg, 41%) as a white solid. mp 202–204 °C. *R*_*f*_ = 0.30 (5% MeOH/dichloromethane). ^1^H NMR (500 MHz, DMSO-*d*_6_): δ 7.39 (d, *J* = 8.4 Hz, 1H), 6.67 (s, 2H), 6.65–6.60 (m, 2H), 6.58 (s, 2H), 5.12 (s, 2H), 4.20 (d, *J* = 10.1 Hz, 1H), 3.80 (d, *J* = 1.6 Hz, 6H), 3.72 (s, 6H), 3.63 (s, 3H), 3.29–3.19 (m, 1H), 1.65 (d, *J* = 10.7 Hz, 2H), 1.50 (d, *J* = 5.2 Hz, 2H), 1.42 (d, *J* = 10.9 Hz, 1H), and 1.09–0.93 (m, 5H). ^13^C {^1^H} NMR (126 MHz, DMSO-*d*_6_): δ 160.2, 157.1, 152.9, 152.1, 149.4, 136.8, 132.2, 131.7, 126.6, 124.2, 116.4, 105.5, 105.3, 104.8 (d, *J* = 15.1 Hz), 98.3, 60.0, 56.4, 55.9 (d, *J* = 12.8 Hz), 55.4 (t, *J* = 22.5 Hz), 52.1, 43.6, 33.3, 25.2, and 24.3. HRMS (ESI) *m/z*: [M + H]^+^ calcd for C_29_H_37_O_6_N_6_, 565.2769; found, 565.2765.

##### 6-(4-Chlorophenyl)-7-(cyclohexylamino)-2-((3,4,5-trimethoxybenzyl)amino)imidazo[2,1-*f*][1,2,4]triazin-4(3*H*)-one (**5o**)

It was synthesized according to general procedure B on a 0.5 mmol scale and isolated using 3–5% MeOH/dichloromethane (v/v) to afford **5o** (143 mg, 53%) as a light yellow solid. mp 248–250 °C. *R*_*f*_ = 0.33 (5% MeOH/dichloromethane). ^1^H NMR (500 MHz, DMSO-*d*_6_) δ 11.25 (s, 1H), 7.96 (d, *J* = 8.2 Hz, 2H), 7.45 (d, *J* = 8.2 Hz, 2H), 6.72 (s, 2H), 6.68 (d, *J* = 7.3 Hz, 1H), 4.39 (d, *J* = 6.1 Hz, 2H), 4.36 (s, 1H), 3.76 (s, 6H), 3.62 (s, 3H), 3.00 (q, *J* = 9.2, 8.6 Hz, 1H), 1.67 (d, *J* = 12.4 Hz, 2H), 1.59–1.53 (m, 2H), 1.46–1.39 (m, 1H), 1.15 (q, *J* = 11.3 Hz, 2H), and 1.07–0.98 (m, 3H). ^13^C{^1^H} NMR (126 MHz, DMSO-*d*_6_): δ 152.9, 152.5, 148.6, 136.4, 134.8, 133.5, 132.0, 130.6, 128.4, 128.2, 127.0, 125.8, 104.5, 59.9, 55.9, 55.3, 44.5, 33.42, 25.22, and 24.35. HRMS (ESI) *m/z*: [M + H]^+^ calcd for C_27_H_32_O_4_N_6_Cl, 539.2168; found, 539.2165.

##### 7-(Cyclohexylamino)-6-(4-fluorophenyl)-2-((3,4,5-trimethoxybenzyl)amino)imidazo[2,1-f][1,2,4]triazin-4(3*H*)-one (**5p**)

It was synthesized according to general procedure B on a 1 mmol scale and isolated using 3–5% MeOH/dichloromethane (v/v) to afford **5p** (271 mg, 52%) as a yellow solid. mp 242–244 °C. *R*_*f*_ = 0.34 (5% MeOH/dichloromethane). ^1^H NMR (500 MHz, DMSO-*d*_6_): δ 11.20 (s, 1H), 7.97 (dd, *J* = 5.5, 9.0 Hz, 2H), 7.23 (t, *J* = 8.9 Hz, 2H), 6.72 (s, 2H), 6.71–6.63 (m, 1H), 4.39 (d, *J* = 5.7 Hz, 2H), 4.33 (d, *J* = 8.8 Hz, 1H), 3.76 (s, 6H), 3.62 (s, 3H), 3.05–2.90 (m, 1H), 1.67 (d, *J* = 9.8 Hz, 2H), 1.56 (d, *J* = 13.1 Hz, 2H), 1.41 (s, 1H), 1.20 (d, *J* = 23.3 Hz, 3H), and 1.03 (d, *J* = 8.4 Hz, 2H). ^13^C{^1^H} NMR (126 MHz, DMSO-*d*_6_): δ 160.9 (d, *J* = 243.3 Hz), 152.9, 152.3, 148.5, 136.4, 134.8, 131.5, 131.1 (d, *J* = 2.8 Hz), 128.6, 127.3 (d, *J* = 10.5 Hz), 125.6, 115.2 (d, *J* = 21.1 Hz), 104.5 (d, *J* = 13.3 Hz), 68.5, 59.9 (d, *J* = 6.0 Hz), 55.9, 55.8 (d, *J* = 10.1 Hz), 44.5, 29.6, and 24.4 (d, *J* = 29.4 Hz). HRMS (ESI) *m/z*: [M + H]^+^ calcd for C_27_H_32_O_4_N_6_F, 523.2464; found, 523.246.

##### 7-(Cyclohexylamino)-6-(2-fluorophenyl)-2-((3,4,5-trimethoxybenzyl)amino)imidazo[2,1-*f*][1,2,4]triazin-4(3*H*)-one (**5q**)

It was synthesized according to general procedure B on a 0.5 mmol scale and isolated using 3–5% MeOH/dichloromethane (v/v) to afford **5q** (156 mg, 60%) as a yellow solid. mp 239–241 °C. *R*_*f*_ = 0.28 (5% MeOH/dichloromethane). ^1^H NMR (500 MHz, DMSO-*d*_6_): δ 11.03 (s, 1H), 7.58 (d, *J* = 1.9 Hz, 1H), 7.45–7.33 (m, 1H), 7.31–7.19 (m, 2H), 6.73 (s, 2H), 6.53 (t, *J* = 5.8 Hz, 1H), 4.47–4.42 (m, 1H), 4.38 (d, *J* = 5.8 Hz, 2H), 3.76 (s, 6H), 3.62 (s, 3H), 3.18–3.06 (m, 1H), 1.63 (d, *J* = 8.8 Hz, 2H), 1.50 (d, *J* = 13.4 Hz, 2H), 1.37 (d, *J* = 3.8 Hz, 1H), 1.12–0.97 (m, 3H), and 0.91 (t, *J* = 12.5 Hz, 2H). ^13^C{^1^H} NMR (126 MHz, DMSO-*d*_6_): δ 159.3 (d, *J* = 246.1 Hz), 152.9, 151.9, 148.1, 136.4, 134.7, 133.1, 131.1 (d, *J* = 15.5 Hz), 129.2 (d, *J* = 8.3 Hz), 125.1, 124.3, 122.6 (d, *J* = 14.7 Hz), 121.6, 115.5, 104.6 (d, *J* = 13.3 Hz), 59.9 (d, *J* = 3.7 Hz), 55.9, 55.8, 52.9, 44.5, 33.2, 25.2, and 24.3. HRMS (ESI) *m/z*: [M + H]^+^ calcd for C_27_H_32_O_4_N_6_F, 523.2452; found, 523.2454.

##### 7-(Cyclohexylamino)-6-(4-(trifluoromethyl)phenyl)-2-((3,4,5-trimethoxybenzyl)amino)imidazo[2,1-*f*][1,2,4]triazin-4(3*H*)-one (**5r**)

It was synthesized according to general procedure B on a 0.5 mmol scale and isolated using 3–5% MeOH/dichloromethane (v/v) to afford **5r** (74 mg, 26%) as yellow solid. mp 253–255 °C. *R*_*f*_ = 0.68 (5% MeOH/dichloromethane). ^1^H NMR (500 MHz, DMSO-*d*_6_): δ 11.21 (s, 1H), 8.15 (d, *J* = 8.5 Hz, 2H), 7.74 (d, *J* = 8.7 Hz, 2H), 6.72 (s, 2H), 6.61 (t, *J* = 6.0 Hz, 1H), 4.51 (d, *J* = 9.0 Hz, 1H), 4.40 (d, *J* = 5.7 Hz, 2H), 3.77 (s, 6H), 3.61 (s, 3H), 3.08–2.97 (m, 1H), 1.76–1.64 (m, 2H), 1.60–1.51 (m, 2H), 1.41 (s, 1H), 1.17 (q, *J* = 11.3, 12.1 Hz, 2H), and 1.03 (t, *J* = 11.8 Hz, 3H). ^13^C{^1^H} NMR (126 MHz, DMSO-*d*_6_): δ 152.9, 152.2, 148.3, 138.6, 136.4, 134.8, 133.0, 127.7, 126.4, 126.2, 126.1, 125.6, 125.4, 125.2, 123.5, 104.5, 104.4, 60.5, 59.9, 59.4, 55.9, 55.8, 55.5, 55.3, 44.5, 33.5, 25.3, and 24.3. HRMS (ESI) *m/z*: [M + H]^+^ calcd for C_28_H_32_O_4_N_6_F_3_, 573.2432; found, 573.2428.

##### 4-(7-(Cyclohexylamino)-4-oxo-2-((3,4,5-trimethoxybenzyl)amino)-3,4-dihydroimidazo[2,1-*f*][1,2,4]triazin-6-yl)benzenesulfonamide (**5s**)

It was synthesized according to general procedure B on a 0.5 mmol scale and isolated using 3–5% MeOH/dichloromethane (v/v) to afford **5s** (138 mg, 47%) as yellow solid. mp 268–270 °C. *R*_*f*_ = 0.38 (5% MeOH/dichloromethane). ^1^H NMR (500 MHz, DMSO-*d*_6_): δ 11.22 (s, 1H), 8.12 (d, *J* = 8.2 Hz, 2H), 7.85 (d, *J* = 8.3 Hz, 2H), 7.34 (s, 2H), 6.73 (s, 2H), 6.66–6.61 (m, 1H), 4.50 (d, *J* = 9.1 Hz, 1H), 4.41 (d, *J* = 5.7 Hz, 2H), 3.78 (s, 6H), 3.63 (s, 3H), 3.04 (tt, *J* = 9.8, 6.1 Hz, 1H), 1.71 (d, *J* = 12.8 Hz, 2H), 1.58 (d, *J* = 11.3 Hz, 2H), 1.47–1.40 (m, 1H), 1.19 (q, *J* = 11.2, 10.7 Hz, 2H), and 1.06 (dt, *J* = 20.6, 12.2 Hz, 3H). ^13^C{^1^H} NMR (126 MHz, DMSO-*d*_6_): δ 152.9, 152.2, 148.3, 141.4, 137.8, 136.4, 134.7, 133.0, 127.8, 126.2, 125.3, 104.6, 104.5, 79.2, 79.2, 60.0, 55.9, 55.8, 55.4, 44.5, 33.5, 25.2, and 24.4. HRMS (ESI) *m*/*z*: [M + H]^+^ calcd for C_27_H_34_N_7_O_6_S, 584.2216; found, 584.2213.

##### 7-(Cyclohexylamino)-6-cyclopropyl-2-((3,4,5-trimethoxybenzyl)amino)imidazo[2,1-*f*][1,2,4]triazin-4(3*H*)-one (**5u**)

It was synthesized according to general procedure B on a 0.5 mmol scale and isolated using 3–5% MeOH/dichloromethane (v/v) to afford **5u** (176 mg, 75%) as a yellow solid. mp 253–255 °C. *R*_*f*_ = 0.25 (3% MeOH/dichloromethane). ^1^H NMR (500 MHz, DMSO-*d*_6_): δ 10.81 (s, 1H), 6.70 (s, 2H), 6.43 (t, *J* = 5.8 Hz, 1H), 4.35 (d, *J* = 9.0 Hz, 1H), 4.32 (d, *J* = 5.8 Hz, 2H), 3.75 (s, 6H), 3.61 (s, 3H), 3.32 (s, 1H), 1.92 (ddd, *J* = 13.2, 8.3, 5.0 Hz, 1H), 1.76 (d, *J* = 9.1 Hz, 2H), 1.62 (d, *J* = 4.7 Hz, 2H), 1.47 (d, *J* = 7.9 Hz, 1H), 1.13 (td, *J* = 17.0, 14.9, 7.5 Hz, 5H), 0.78 (ddd, *J* = 8.0, 5.9, 3.5 Hz, 2H), and 0.73 (dt, *J* = 5.0, 2.7 Hz, 2H). ^13^C{^1^H} NMR (126 MHz, DMSO-*d*_6_): δ 152.8, 151.4, 147.9, 136.4, 134.8, 132.1, 131.7, 123.3, 104.6, 60.0, 59.9, 56.4, 55.9, 55.8, 55.3, 54.2, 44.5, 33.6, 24.4, and 7.2. HRMS (ESI) *m/z*: [M + H]^+^ calcd for C_24_H_33_N_6_O_4_, 469.2547; found, 469.2548.

##### 7-(Cyclohexylamino)-6-methyl-2-((3,4,5-trimethoxybenzyl)amino)imidazo[2,1-*f*][1,2,4]triazin-4(3*H*)-one (**5v**)

It was synthesized according to general procedure B on a 0.5 mmol scale and isolated using 3–5% MeOH/dichloromethane (v/v) to afford **5v** (103 mg, 48%) as a gray solid. mp 167–169 °C. *R*_*f*_ = 0.52 (5% MeOH/dichloromethane). ^1^H NMR (500 MHz, DMSO-*d*_6_): δ 10.78 (s, 1H), 6.70 (s, 2H), 6.38 (s, 1H), 4.33 (d, *J* = 5.8 Hz, 2H), 4.25 (d, *J* = 8.5 Hz, 1H), 3.76 (s, 6H), 3.62 (s, 3H), 3.21 (s, 1H), 2.15 (s, 3H), 1.72 (s, 2H), 1.61 (s, 2H), 1.49 (s, 1H), and 1.12 (d, *J* = 8.2 Hz, 5H). ^13^C{^1^H} NMR (126 MHz, DMSO-*d*_6_): δ 152.8, 151.4, 147.9, 136.5, 134.7, 131.8, 126.3, 123.4, 104.6, 60.4, 56.5, 56.1 (d, *J* = 56.4 Hz), 55.5 (d, *J* = 56.8 Hz), 53.8 (d, *J* = 16.5 Hz), 44.4, 33.8, and 24.3 (d, *J* = 32.5 Hz). HRMS (ESI) *m/z*: [M + H]^+^ calcd for C_21_H_31_O_4_N_6_, 431.2401; found, 431.2405.

##### 6-Phenyl-2,7-bis((3,4,5-trimethoxybenzyl)amino)imidazo[2,1-f][1,2,4]triazin-4(3*H*)-one (**5w**)

It was synthesized according to general procedure B on a 0.5 mmol scale and isolated using 3–5% MeOH/dichloromethane (v/v) to afford **5w** (114 mg, 38%) as a yellow solid. mp 267–269 °C. *R*_*f*_ = 0.44 (5% MeOH/dichloromethane). ^1^H NMR (500 MHz, DMSO-*d*_6_): δ 11.06 (s, 1H), 7.82–7.76 (m, 2H), 7.39 (t, *J* = 7.6 Hz, 2H), 7.25 (t, *J* = 7.4 Hz, 1H), 6.76 (s, 2H), 6.56 (t, *J* = 5.8 Hz, 1H), 6.38 (s, 2H), 5.58 (t, *J* = 7.0 Hz, 1H), 4.40 (d, *J* = 5.8 Hz, 2H), 4.29 (d, *J* = 6.9 Hz, 2H), 3.75 (s, 6H), 3.60 (s, 3H), 3.53 (s, 3H), and 3.50 (s, 6H). ^13^C{^1^H} NMR (126 MHz, DMSO-*d*_6_): δ 152.8, 152.5, 151.9, 148.1, 136.5, 136.1, 135.9, 134.6, 134.4, 132.1, 129.0, 128.3, 126.3 (d, *J* = 28.6 Hz), 126.0, 125.2, 104.9, 104.3 (d, *J* = 23.9 Hz), 59.9, 55.9, 55.7, 55.4 (d, *J* = 12.9 Hz), 49.0, and 44.6. HRMS (ESI) *m/z*: [M + H]^+^ calcd for C_31_H_35_O_6_N_7_, 603.2491; found, 603.2489.

##### 2-Amino-7-(cyclohexylamino)-6-phenylimidazo[2,1-*f*][1,2,4]triazin-4(3*H*)-one (**6a**)

It was synthesized according to general procedure C on a 0.5 mmol scale and isolated using 3–7% MeOH/dichloromethane (v/v) to afford **6a** (from **5f**, 42 mg, 26%; from **5l**, 112 mg, 69%) as a white solid. mp 274–276 °C. *R*_*f*_ = 0.14 (5% MeOH/dichloromethane). ^1^H NMR (500 MHz, DMSO-*d*_6_): δ 11.06 (s, 1H), 7.96 (d, *J* = 8.0 Hz, 2H), 7.40 (t, *J* = 7.7 Hz, 2H), 7.23 (t, *J* = 7.5 Hz, 1H), 6.14 (s, 2H), 4.21 (d, *J* = 8.8 Hz, 1H), 3.11 (t, *J* = 9.8 Hz, 1H), 1.79–1.68 (m, 2H), 1.68–1.56 (m, 2H), 1.47 (s, 1H), 1.20 (q, *J* = 7.1, 8.8 Hz, 2H), and 1.08 (dd, *J* = 7.7, 20.7 Hz, 3H). ^13^C{^1^H} NMR (126 MHz, DMSO-*d*_6_): δ 152.2, 149.6, 134.6, 131.3, 129.8, 128.3, 126.4, 125.7, 125.6, 54.8, 33.44 (t, *J* = 54.4 Hz), 25.3, and 24.41 (d, *J* = 26.6 Hz). HRMS (ESI) *m/z*: [M + H]^+^ calcd for C_17_H_21_ON_6_, 325.1771; found, 325.1769.

##### 2-Amino-7-(cyclohexylamino)-6-(4-methoxyphenyl)imidazo[2,1-*f*][1,2,4]triazin-4(3H)-one (**6b**)

It was synthesized according to general procedure C on a 0.2 mmol scale and isolated using 3–7% MeOH/dichloromethane (v/v) to afford **6b** (42 mg, 60%) as a white solid. mp 282–284 °C. *R*_*f*_ = 0.25 (5% MeOH/dichloromethane). ^1^H NMR (500 MHz, DMSO-*d*_6_): δ 10.98 (s, 1H), 7.90 (d, *J* = 9.0 Hz, 2H), 6.97 (d, *J* = 9.0 Hz, 2H), 6.11 (s, 2H), 4.13 (d, *J* = 8.5 Hz, 1H), 3.78 (s, 3H), 3.12–3.01 (m, 1H), 1.73 (d, *J* = 12.8 Hz, 2H), 1.64 (s, 2H), 1.48 (s, 1H), and 1.20–1.09 (m, 5H). ^13^C{^1^H} NMR (126 MHz, DMSO-*d*_6_): δ 158.0, 152.0, 149.4, 130.4, 130.3, 127.1, 126.9 (d, *J* = 17.2 Hz), 125.3, 113.7, 55.6, 55.0 (t, *J* = 21.9, 20.0 Hz), 33.4, 25.4, and 24.5. HRMS (ESI) *m/z*: [M + H]^+^ calcd for C_18_H_23_O_2_N_6_, 355.1877; found, 355.1877.

##### 2-Amino-6-(benzo[d][1,3]dioxol-5-yl)-7-(cyclohexylamino)imidazo[2,1-f][1,2,4]triazin-4(3H)-one (**6c**)

It was synthesized according to general procedure C on a 0.2 mmol scale and isolated using 3–7% MeOH/dichloromethane (v/v) to afford **6c** (44 mg, 60%) as a brown solid. mp 250–252 °C. *R*_*f*_ = 0.17 (5% MeOH/dichloromethane). ^1^H NMR (500 MHz, DMSO-*d*_6_): δ 10.90 (s, 1H), 7.50 (d, *J* = 12.6 Hz, 2H), 6.95 (d, *J* = 8.2 Hz, 1H), 6.15 (s, 2H), 6.03 (s, 2H), 4.16 (d, *J* = 8.4 Hz, 1H), 3.08 (d, *J* = 8.5 Hz, 1H), 1.73 (d, *J* = 13.7 Hz, 2H), 1.67–1.60 (m, 2H), 1.49 (s, 1H), and 1.23–1.09 (m, 5H). ^13^C{^1^H} NMR (126 MHz, DMSO-*d*_6_): δ 152.1, 149.5, 147.3, 145.9, 130.5, 130.2, 128.8, 125.4, 119.2 (d, *J* = 19.2 Hz), 108.3, 106.0 (d, *J* = 21.5 Hz), 100.9, 54.9, 33.4, 25.5, and 24.3. HRMS (ESI) *m/z*: [M + H]^+^ calcd for C_18_H_21_O_3_N_6_, 369.167; found, 369.1669.

##### 2-Amino-7-(cyclohexylamino)-6-(2,4-dimethoxyphenyl)imidazo[2,1-*f*][1,2,4]triazin-4(3H)-one (**6d**)

It was synthesized according to general procedure C on a 0.2 mmol scale and isolated using 3–7% MeOH/dichloromethane (v/v) to afford **6d** (from **5g**, 45 mg, 59%; from **5n**, 51 mg, 66%) as a brown solid. mp 298–300 °C. *R*_*f*_ = 0.12 (5% MeOH/dichloromethane). ^1^H NMR (500 MHz, DMSO-*d*_6_): δ 10.90 (s, 1H), 7.34 (d, *J* = 8.4 Hz, 1H), 6.72–6.53 (m, 2H), 6.08 (s, 2H), 4.16 (d, *J* = 10.1 Hz, 1H), 3.80 (s, 3H), 3.79 (s, 3H), 3.16 (s, 1H), 1.69–1.59 (m, 2H), 1.49 (s, 2H), 1.40 (s, 1H), and 0.99 (dt, *J* = 9.1, 21.9 Hz, 5H). ^13^C{^1^H} NMR (126 MHz, DMSO-*d*_6_): δ 160.2, 157.1, 151.9, 149.2, 132.2, 131.6, 125.8, 124.7, 116.5, 105.3 (d, *J* = 22.9 Hz), 98.4 (d, *J* = 19.2 Hz), 55.5, 55.2, 52.0, 33.3 (d, *J* = 21.5 Hz), 25.3 (d, *J* = 17.1 Hz), and 24.2 (d, *J* = 25.2 Hz). HRMS (ESI) *m/z*: [M + H]^+^ calcd for C_19_H_25_O_3_N_6_, 385.1983; found, 385.1982.

##### 2-Amino-6-(4-chlorophenyl)-7-(cyclohexylamino)imidazo[2,1-*f*][1,2,4]triazin-4(3H)-one (**6e**)

It was synthesized according to general procedure C on a 0.3 mmol scale and isolated using 3–7% MeOH/dichloromethane (v/v) to afford **6e** (49 mg, 46%) as a white solid. mp 296–298 °C. *R*_*f*_ = 0.26 (5% MeOH/dichloromethane). ^1^H NMR (500 MHz, DMSO-*d*_6_): δ 11.13 (s, 1H), 7.99 (d, *J* = 8.7 Hz, 2H), 7.45 (d, *J* = 8.7 Hz, 2H), 6.17 (s, 2H), 4.26 (d, *J* = 8.4 Hz, 1H), 3.06 (d, *J* = 8.5 Hz, 1H), 1.74 (d, *J* = 12.5 Hz, 2H), 1.63 (s, 2H), 1.48 (s, 1H), 1.25–1.16 (m, 2H), and 1.10 (s, 3H). ^13^C{^1^H} NMR (126 MHz, DMSO-*d*_6_): δ 152.1, 149.5, 133.5, 131.4, 130.8, 129.0, 128.3 (d, *J* = 8.9 Hz, 1H), 127.5–126.9 (m), 126.0, 55.1, 33.4, 25.4, and 244 (d, *J* = 20.7 Hz, 1H),. HRMS (ESI) *m/z*: [M + H]^+^ calcd for C_17_H_20_ON_6_Cl, 359.1382; found, 359.1382.

##### 2-Amino-6-(4-bromophenyl)-7-(cyclohexylamino)imidazo[2,1-*f*][1,2,4]triazin-4(3H)-one (**6f**)

It was synthesized according to general procedure C on a 0.2 mmol scale and isolated using 3–7% MeOH/dichloromethane (v/v) to afford **6f** (64 mg, 80%) as a light yellow solid. mp 295–297 °C. *R*_*f*_ = 0.24 (5% MeOH/dichloromethane). ^1^H NMR (500 MHz, DMSO-*d*_6_): δ 11.11 (s, 1H), 7.95 (d, *J* = 8.7 Hz, 2H), 7.60 (d, *J* = 8.5 Hz, 2H), 6.17 (s, 2H), 4.27 (d, *J* = 8.4 Hz, 1H), 1.75 (d, *J* = 12.3 Hz, 2H), 1.64 (s, 2H), 1.50 (s, 1H), and 1.24–1.11 (m, 5H). ^13^C{^1^H} NMR (126 MHz, DMSO-*d*_6_): δ 152.1, 149.5, 133.8, 131.5, 131.2 (d, *J* = 23.7 Hz), 129.1, 127.5 (d, *J* = 19.2 Hz), 126.0, 119.3, 55.1, 33.4, 25.4, and 24.4. HRMS (ESI) *m/z*: [M + H]^+^ calcd for C_17_H_20_ON_6_Br, 403.0876; found, 403.0874.

##### 2-Amino-7-(cyclohexylamino)-6-(4-fluorophenyl)imidazo[2,1-*f*][1,2,4]triazin-4(3H)-one (**6g**)

It was synthesized according to general procedure C on a 0.3 mmol scale and isolated using 3–7% MeOH/dichloromethane (v/v) to afford **6g** (66 mg, 64%) as a white solid. mp > 300 °C. *R*_*f*_ = 0.28 (5% MeOH/dichloromethane). ^1^H NMR (500 MHz, DMSO-*d*_6_): δ 11.06 (s, 1H), 8.00 (dd, *J* = 5.7, 9.0 Hz, 2H), 7.23 (t, *J* = 8.9 Hz, 2H), 6.14 (s, 2H), 4.22 (d, *J* = 8.4 Hz, 1H), 3.16–2.99 (m, 1H), 1.74 (d, *J* = 13.9 Hz, 2H), 1.63 (t, *J* = 4.9 Hz, 2H), 1.48 (s, 1H), 1.23–1.16 (m, 2H), and 1.14–1.03 (m, 3H). ^13^C{^1^H} NMR (126 MHz, DMSO-*d*_6_): δ 160.9 (d, *J* = 243.3 Hz), 152.2, 149.5, 131.1 (d, *J* = 2.9 Hz), 130.9, 129.4, 127.5 (d, *J* = 5.8 Hz), 125.7, 115.1 (d, *J* = 21.5 Hz), 54.9, 33.3, 25.9, and 24.2. HRMS (ESI) *m/z*: [M + H]^+^ calcd for C_17_H_20_ON_6_F, 343.1677; found, 343.1676.

##### 2-Amino-7-(cyclohexylamino)-6-(4-(trifluoromethyl)phenyl)imidazo[2,1-*f*][1,2,4]triazin-4(3H)-one (**6h**)

It was synthesized according to general procedure C on a 0.2 mmol scale and isolated using 3–7% MeOH/dichloromethane (v/v) to afford **6h** (47 mg, 60%) as a white solid. mp > 300 °C. *R*_*f*_ = 0.57 (20% Acetone/Dichloromethane). ^1^H NMR (500 MHz, DMSO-*d*_6_): δ 11.16 (s, 1H), 8.19 (d, *J* = 8.4 Hz, 2H), 7.75 (d, *J* = 8.7 Hz, 2H), 6.22 (s, 2H), 4.37 (d, *J* = 8.5 Hz, 1H), 3.11 (t, *J* = 9.2 Hz, 1H), 1.79–1.73 (m, 2H), 1.67–1.60 (m, 2H), 1.49 (s, 1H), and 1.17 (d, *J* = 52.8 Hz, 5H). ^13^C{^1^H} NMR (126 MHz, DMSO-*d*_6_): δ 152.3, 149.7, 138.6, 132.3, 128.5, 126.4, 126.2, 125.7 (dd, *J* = 16.4, 23.6 Hz), 125.3 (d, *J* = 28.0 Hz), 123.5, 54.9, 33.4, 25.4, and 24.3. HRMS (ESI) *m/z*: [M + H]^+^ calcd for C_18_H_20_ON_6_F_3_, 393.1645; found, 393.1645.

##### 4-(2-Amino-7-(cyclohexylamino)-4-oxo-3,4-dihydroimidazo[2,1-*f*][1,2,4]triazin-6-yl)benzenesulfonamide (**6i**)

It was synthesized according to general procedure C on a 0.2 mmol scale and isolated using 3–7% MeOH/dichloromethane (v/v) to afford **6i** (44 mg, 54%) as a white solid. mp 177–180 °C. *R*_*f*_ = 0.18 (7% MeOH/dichloromethane). ^1^H NMR (500 MHz, DMSO-*d*_6_): δ 11.13 (s, 1H), 8.14 (d, *J* = 8.1 Hz, 2H), 7.86–7.81 (m, 2H), 7.33 (s, 2H), 6.19 (s, 2H), 4.37 (d, *J* = 8.5 Hz, 1H), 3.13–3.04 (m, 1H), 1.76 (d, *J* = 12.6 Hz, 2H), 1.67–1.60 (m, 2H), 1.48 (s, 1H), 1.21 (dd, *J* = 16.0, 7.3 Hz, 2H), and 1.12 (d, *J* = 8.4 Hz, 3H). ^13^C{^1^H} NMR (126 MHz, DMSO-*d*_6_): δ 152.3, 149.6, 141.4, 137.9, 132.4, 128.6, 126.4, 125.9, 125.8, 125.4, 55.3, 33.4, 25.4, 24.5, and 24.3. HRMS (ESI) *m/z*: [M + H]^+^ calcd for C_17_H_22_O_3_N_7_S, 404.1430; found, 404.1427.

##### 2-Amino-7-(cyclohexylamino)-6-cyclopropylimidazo[2,1-*f*][1,2,4]triazin-4(3*H*)-one (**6j**)

It was synthesized according to general procedure C on a 0.5 mmol scale and isolated using 3–7% MeOH/dichloromethane (v/v) to afford **6j** (65 mg, 45%) as a white solid. mp 127–130 °C. *R*_*f*_ = 0.11 (5% MeOH/dichloromethane). ^1^H NMR (500 MHz, DMSO-*d*_6_): δ 10.77 (s, 1H), 6.00 (s, 2H), 4.18 (d, *J* = 8.8 Hz, 1H), 3.31 (s, 1H), 1.93 (ddd, *J* = 5.1, 8.2, 13.2 Hz, 1H), 1.82 (d, *J* = 9.8 Hz, 2H), 1.72–1.63 (m, 2H), 1.54 (d, *J* = 12.5 Hz, 1H), 1.24–1.13 (m, 5H), 0.79 (dt, *J* = 2.7, 8.2 Hz, 2H), and 0.75 (dt, *J* = 2.5, 4.9 Hz, 2H). ^13^C{^1^H} NMR (126 MHz, DMSO-*d*_6_): δ 151.5, 149.2, 132.5, 131.4, 123.6, 55.8, 55.0, 54.2, 33.6, 24.9 (d, *J* = 121.0 Hz), 8.0 (d, *J* = 17.9 Hz), and 7.2. HRMS (ESI) *m/z*: [M + H]^+^ calcd for C_14_H_21_ON_6_, 289.1771; found, 289.1770.

##### *N*-(2-(Benzylamino)-4-oxo-6-phenyl-3,4-dihydroimidazo[2,1-*f*][1,2,4]triazin-7-yl)-2,2,2-trifluoroacetamide (**6l**)

It was synthesized according to general procedure C on a 0.5 mmol scale and isolated using 3–7% MeOH/dichloromethane (v/v) to afford **6l** (96 mg, 45%) as a white solid. mp 248–250 °C. *R*_*f*_ = 0.16 (5% MeOH/dichloromethane). ^1^H NMR (500 MHz, DMSO-*d*_6_): δ 11.77 (s, 1H), 11.60 (s, 1H), 7.81–7.76 (m, 2H), 7.46 (t, *J* = 7.7 Hz, 2H), 7.38–7.34 (m, 3H), 7.31 (dd, *J* = 6.5, 8.4 Hz, 2H), 7.28–7.22 (m, 1H), 6.81 (t, *J* = 5.9 Hz, 1H), and 4.35 (d, *J* = 5.8 Hz, 2H). ^13^C{^1^H} NMR (126 MHz, DMSO-*d*_6_): δ 156.5 (q, *J* = 37.3 Hz), 152.2, 149.1, 138.7, 135.9, 132.5, 128.9, 128.8 (d, *J* = 7.7 Hz),128.3, 127.9, 127.2, 125.9, 125.7, 117.0, 114.7, and 44.3. HRMS (ESI) *m/z*: [M + H]^+^ calcd for C_20_H_16_O_2_N_6_F_3_, 429.1281; found, 429.1279.

##### 2,7-Diamino-6-phenylimidazo[2,1-*f*][1,2,4]triazin-4(3*H*)-one (**6m**)

It was synthesized according to general procedure D on a 0.2 mmol scale and isolated using 3–10% MeOH/dichloromethane (v/v) to afford **6m** (from **5f**, 17 mg, 14%; from **5l**, 87 mg, 72%) as a gray solid. mp 260–262 °C. *R*_*f*_ = 0.13 (5% MeOH/dichloromethane). ^1^H NMR (500 MHz, DMSO-*d*_6_): δ 10.90 (s, 1H), 7.83 (d, *J* = 7.2 Hz, 2H), 7.57–7.28 (m, 2H), 7.25–7.08 (m, 1H), 6.10 (s, 2H), and 5.29 (s, 2H). ^13^C{^1^H} NMR (126 MHz, DMSO-*d*_6_): δ 152.0, 149.5, 134.7, 132.2, 128.4, 125.6, 124.9 (d, *J* = 16.0 Hz)., 123.9, and 123.7. HRMS (ESI) *m/z*: [M + H]^+^ calcd for C_11_H_11_ON_6_, 243.0989; found, 243.0988.

##### 2,7-Diamino-6-(4-chlorophenyl)imidazo[2,1-*f*][1,2,4]triazin-4(3*H*)-one (**6n**)

It was synthesized according to general procedure D on a 0.2 mmol scale and isolated using 3–10% MeOH/dichloromethane (v/v) to afford **6n** (28 mg, 51%) as a dark green solid. mp 312–314 °C. *R*_*f*_ = 0.25 (5% MeOH/dichloromethane). ^1^H NMR (500 MHz, DMSO-*d*_6_): δ 10.90 (s, 1H), 7.91–7.75 (m, 2H), 7.51–7.31 (m, 2H), 6.09 (s, 2H), and 5.36 (s, 2H). ^13^C{^1^H} NMR (126 MHz, DMSO-*d*_6_): δ 151.9, 149.4, 133.6, 132.4, 129.8, 128.3, 126.44 (d, *J* = 12.8 Hz), 124.1, and 122.5. HRMS (ESI) *m/z*: [M + H]^+^ calcd for C_11_H_10_ ON_6_Cl, 277.0599; found, 277.0597.

##### 2,7-Diamino-6-(4-fluorophenyl)imidazo[2,1-*f*][1,2,4]triazin-4(3*H*)-one (**6o**)

It was synthesized according to general procedure D on a 0.1 mmol scale and isolated using 3–10% MeOH/dichloromethane (v/v) to afford **6o** (15 mg, 58%) as a brown solid. mp 273–275 °C. *R*_*f*_ = 0.11 (5% MeOH/dichloromethane). ^1^H NMR (500 MHz, DMSO-*d*_6_): δ 10.92 (s, 1H), 7.88–7.81 (m, 2H), 7.21 (t, *J* = 8.9 Hz, 2H), 6.10 (s, 2H), and 5.28 (s, 2H). ^13^C{^1^H} NMR (126 MHz, DMSO-*d*_6_): δ 160.5 (d, *J* = 242.4 Hz), 152.0, 149.5, 131.9, 131.2 (d, *J* = 3.2 Hz), 126.7 (d, *J* = 7.0 Hz), 123.9, 123.0, and 115.2 (d, *J* = 21.1 Hz). HRMS (ESI) *m/z*: [M + H]^+^ calcd for C_11_H_10_ON_6_F, 261.0895; found, 261.0892.

##### 2,7-Diamino-6-(4-(trifluoromethyl)phenyl)imidazo[2,1-*f*][1,2,4]triazin-4(3*H*)-one (**6p**)

It was synthesized according to general procedure D on a 0.2 mmol scale and isolated using 3–10% MeOH/dichloromethane (v/v) to afford **6p** (42 mg, 68%) as a dark green solid. mp >300 °C. *R*_*f*_ = 0.62 (10% MeOH/dichloromethane). ^1^H NMR (500 MHz, DMSO-*d*_6_): δ 10.92 (s, 1H), 8.03 (d, *J* = 8.5 Hz, 2H), 7.70 (d, *J* = 8.5 Hz, 2H), 6.13 (s, 2H), and 5.50 (s, 2H). ^13^C{^1^H} NMR (126 MHz, DMSO-*d*_6_): δ 152.1, 149.5, 138.7, 133.4, 125.6 (d, *J* = 27.0 Hz), 125.2 (dd, *J* = 7.2, 13.9 Hz), 124.9 (d, *J* = 8.2 Hz), 124.6, 123.5, and 121.8. HRMS (ESI) *m/z*: [M + H]^+^ calcd for C_12_H_10_ON_6_F_3_, 311.0863; found, 311.0862.

## Data Availability

The data underlying this study are available in the published article and its Supporting Information.

## Supporting Information Available

Experimental procedure and ^1^H and ^13^C{^1^H} NMR spectra for all compounds along with the X-ray crystallographic data for **4d** and **5l** (PDF)
