# Supplementary material for: N-Edited Guanine Isosteres
Source: J Org Chem. 2023 Jul 11;88(14):9823–34. doi: 10.1021/acs.joc.3c00467 (PMC10367071; doi:10.1021/acs.joc.3c00467)

Supplementary Information

N-Edited Guanine Isosteres

Xin Li[a], Marina Diguele Romero[a], Sona Tcaturian[a], Katarzyna Kurpiewska[b] and Alexander Dömling[a]*

[a]University of Groningen, Department of Drug Design, A. Deusinglaan 1, 9713 AV Groningen, The Netherlands

[b]Department of Crystal Chemistry and Crystal Physics Faculty of Chemistry, Jagiellonian University, 30-387 Kraków, Poland

*Corresponding e-mail: alexander.domling@upol.cz.

Table of contents

1.Table S1: Optimization of the Benzyl-deprotection Conditions -------------------S2-S3

2. Crystal sample preparation procedure and X-ray Crystallographic Data --------S3-S6

3. The pK predication by ChemAxon software--S6

4. The comparison of ^{2}D-NMR spectrum of **6a-D** and ^{1}H-NMR spectrum of **6a**------S7

5. The comparison of ^{2}D-NMR spectrum of **6m-D** and ^{1}H-NMR spectrum of **6m**----S8

6. Proposed Mechanism --S8

7. ^{1}H and ^{13}C{^{1}H} NMR spectrums of final compounds-----------------------------S9-S49

Table S1. Optimization of the Benzyl-deprotection Conditions

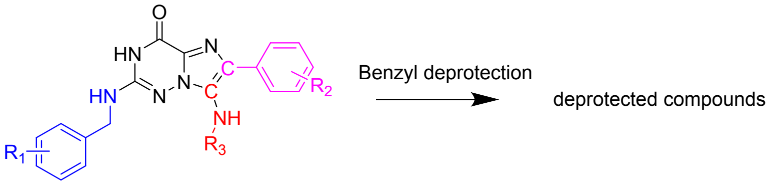

Entry	R1	R2	R3	Condition	Deprotected compounds	Yield
1	H	H	t-butyl	10% Pd/C, H_2 (balloon), MeOH, r.t., overnight	ND	
2	H	H	t-butyl	10% Pd/C, H_2 (balloon), MeOH, 55 °C, 4 h,	ND	
3	H	H	t-butyl	10%TFA/DCE, 100 °C, microwave 10 min; EA, 1N NaOH	ND	
4	H	H	t-butyl	TfOH, 55 °C, 4 h	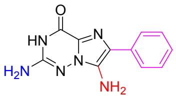	41%
5	H	H	t-butyl	0.1 M TFA, r,t, 10 min; 80 °C reflux overnight	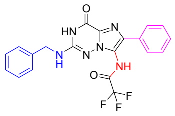	45%
6	H	Cl	t-butyl	10% Pd/C, H_2(balloon), MeOH, r.t., overnight	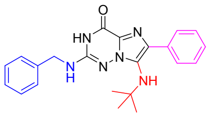	52%
7	H	Cl	t-butyl	10%TFA/DCE, 100 °C, microwave 10 min; EA, 1N NaOH	ND	
8	H	Cl	t-butyl	10% Pd/C, H_2(balloon), MeOH, 55 °C, 4 h,	ND	
9	4-OMe	H	t-butyl	10% Pd/C, H_2(balloon), MeOH, r.t., overnight	ND	
10	4-OMe	H	t-butyl	10%TFA/DCE, 100 °C, microwave 10 min; EA, 1N NaOH	ND	
11	4-OMe	H	t-butyl	10% Pd/C, H_2(balloon), MeOH, 55 °C, 4 h,	ND	
12	2,4-dimethoxy	H	t-butyl	0.1 M TFA, rt, 10 min; 80 °C reflux overnight; EA, aq. $NaHCO_3$	ND	
13	3,4,5-trimethoxy	H	t-butyl	0.1 M TFA, rt, 10 min; 80 °C reflux overnight;EA, aq. $NaHCO_3$	ND	
14	2,4-dimethoxy	H	Cyclohexyl	0.1 M TFA, rt, 10 min; 80 °C reflux overnight; EA, aq. $NaHCO_3$	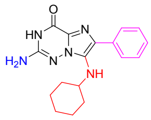	42%
15	2,4-dimethoxy	H	Cyclohexyl	0.1 M TFA, r,t, overnight	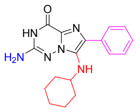	22%
16	3,4,5-trimethoxy	H	Cyclohexyl	0.1 M TFA, rt, 10 min; 80 °C reflux overnight; EA, aq. $NaHCO_3$	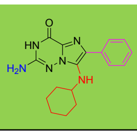	69%

17	3,4,5-trimethoxy	H	Cyclohexyl	0.1 M TFA, r,t, overnight	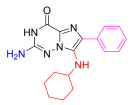	54%
18	3,4,5-trimethoxy	H	Cyclohexyl	20% TFA/DCM, rt, 24 h	ND	
19	3,4,5-trimethoxy	H	Cyclohexyl	50% TFA/DCM, rt, 24 h	ND	
20	3,4,5-trimethoxy	H	Cyclohexyl	TfOH, 55 °C, 4 h	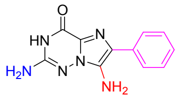	72%

ND: not detected.

Crystal sample preparation:

recrystallization in EtOH

20-30 mg sample powder was added into the 3 mL vial with around 1 mL EtOH, sealed the vial and heated at 70 °C for several minutes, filtered the solution and removed undissolved material, then transfer the filtrate to a new 3 mL glass vial , cap the vial, and kept still for 2-4 weeks in a dark environment.

Crystal structure determination

X-ray diffraction data for single crystals of compounds 4d and 5l were collected using XtalLAB Synergy-S four-circle diffractometer with a mirror monochromator and a microfocus CuKα radiation source (λ = 1.5418 Å). The CryoStream cryostat system was used to allow low-temperature experiments, performed at 100(2) K. The obtained data sets were processed with CrysAlisPro software.[1] The phase problem was solved with direct methods using SIR2014.[2] Parameters of the obtained model were refined by full-matrix least-squares on F2 using SHELXL-2014/6.[3] Calculations were performed using WinGX integrated system (ver. 2014.1).[4] Figures were prepared with Mercury 4.0 software.[5]

All non-hydrogen atoms were refined anisotropically. All hydrogen atoms attached to carbon atoms were positioned with the idealized geometry and refined using the riding model with the isotropic displacement parameter Uiso[H] = 1.2 Ueq[C] for all but the methyl group, for which Uiso[H] = 1.5 Ueq[C] was applied. Hydrogen atoms bound to nitrogen atoms were located on the Fourier difference map and refined with no restraints on Uiso parameter. The disordered fragments were modeled based on the Fourier difference map inspection and the site occupancies were determined during the refinement procedure. Crystal data and refinement results are shown in Table 1.

Crystallographic data have been deposited with the Cambridge Crystallographic Data Centre (CCDC) as supplementary publication no. CCDC 2192632 (**4d**) and no. CCDC 2190420 (**5l**). Copies of the data can be obtained, free of charge, on application to CCDC , 12 Union Road, Cambridge CB2 1EZ, UK, (fax: +44-(0)1223-336033 or e-mail: deposit@ccdc.cam.ac.uk).

Crystal structure description

Compound **5l** crystallizes in the centrosymmetric space group P2/n. The asymmetric unit

(Figure S2A) consists of two molecules with a very close conformation of the main structural core (RMSD ~ 0.7 Å). For both molecules, a positional disorder is observed. The first molecule exhibits alternative spatial orientations of the cyclohexyl group, with refined site occupancies of 77% and 23%. In the second, one of the methyls of the tri-methoxy benzyl fragment with nearly equal occupancies, 51% and 49% (the less abundant conformers are shown in Figure S1A with carbon atoms in green). These two disordered fragments are located in proximity in the crystal lattice.

The molecule of **5l** adopts a hairpin-like conformation (Figure S2B) with exposure of N-H donors of the hydrogen bonds. This leads to tetramer formation through N-H...O and N-H...N H-bond system (Figure S2C). A large number of C-H...O and C-H...π contacts additionally stabilize the crystal.

Acknowledgments
The crystal structure analysis was performed on the equipment purchased thanks to the financial support of the Ministry of Science and Higher Education, Warsaw, Poland [grant number 6903/IA/SP/2018].

References:

[1] Rigaku-Oxford Diffraction; CrysAlisPro Oxford Diffraction Ltd, Abingdon, England V 1. 171. 36. 2.; (release 27-06-2012 CN) 2006.

[2] Burla M.C., Caliandro R., Carrozzini B. Cascarano G.L., Cuocci C., Giacovazzo C., Mallamo M., Mazzone A., Polidori G., J. Appl. Cryst. 2015, 48 (1), 306–309.

[3] Sheldrick G. M., Acta Cryst. 2008, A64, 112-122.

[4] Farrugia L.J., J. Appl. Cryst. 1999, 32, 837-838.

[5] Macrae C. F., Sovago I., Cottrell S.J., Galek P.T.A., McCabe P., Pidcock E., Platings M., Shields G.P., Stevens J.S., Towler M. and Wood P.A., J. Appl. Cryst. 2020, 53, 226-235.

Table S2. Crystal data and final refinement results for structures **5l** and **4d**.

	**5l**	4d
Empirical moiety formula	2 x C_{27} H_{32} N_6 O_4	C_{13} H_{18} N_5 O_4
Formula weight [g/mol]	1009.17	308.32
Crystal system	Monoclinic	orthorhombic
Space group	P2/*n*	*Pbca*
Unite cell dimensions	a = 18.2386(1) Å<br>b = 9.4353(1) Å<br>c = 30.2359(2) Å<br>α=90°<br>β=107.096(1)°<br>γ=90°	a = 12.59332(16)Å<br>b = 10.19994(14)Å<br>c = 22.0119(2) Å<br>α=90°<br>b= 90°<br>g=90°
Volume [Å^3]	4973.28(7)	2827.44(4)
Z	4	8
D_{calc} [Mg/m^3]	1.348	1.449
μ [mm^{-1}]	0.756	0.923
F(000)	2144	1304
Crystal size [mm^3]	0.5 x 0.15 x 0.02	0.15 x 0.15 x 0.1
Θ range	2.55° to 75.33°	4.78° to 74.97°
Index ranges	$-22 \leq h \leq 22$,<br>$-11 \leq k \leq 10$,<br>$-37 \leq l \leq 37$	$-15 \leq h \leq 216$,<br>$-12 \leq k \leq 11$,<br>$-27 \leq l \leq 27$

Refl. collected	151527	32354
Independent reflections	10196<br>[R(int) = 0.0543]	3072<br>[R(int) = 0.0424]
Completeness [%] to Θ	99.1 (Θ 75.3°)	99.8 (Θ 67.68°)
Absorption correction	Multi-scan	Multi-scan
Tmin. and Tmax.	0.328 and 1.000	0.426 and 1.000
Data/ restraints/parameters	10196 / 0 / 767	3072 / 0 / 203
GooF on F2	1.059	1.079
Final R indices [I>2sigma(I)]	R1= 0.0384,<br>wR2= 0.0903	R1= 0.0769,<br>wR2= 0.2110
R indices (all data)	R1= 0.0415,<br>wR2= 0.0922	R1= 0.0778,<br>wR2= 0.2118
$\Delta\rho_{max}$, $\Delta\rho_{min}$ [e·Å^{-3}]	0.200 and -0.237	0.53 and -0.42

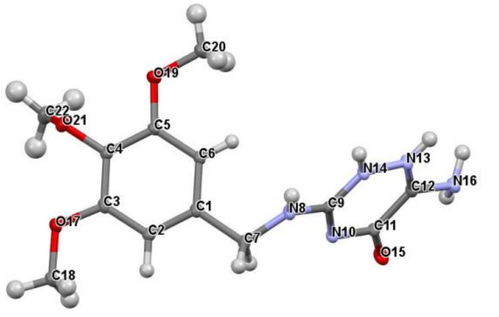

Figure S1. Molecular geometry observed in the crystal structure of compound **4d** showing the atom labelling scheme. The positional disorder within the benzene ring is observed with equal site occupancy (50:50). Displacement ellipsoids of non-hydrogen atoms are drawn at the 30% probability level. H atoms are presented as small spheres with an arbitrary radius.

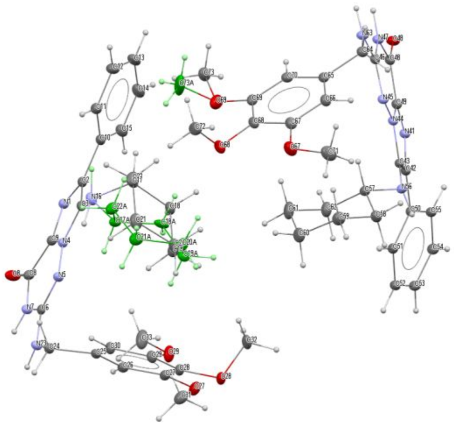

A

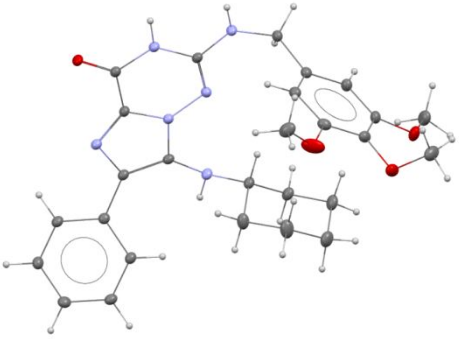

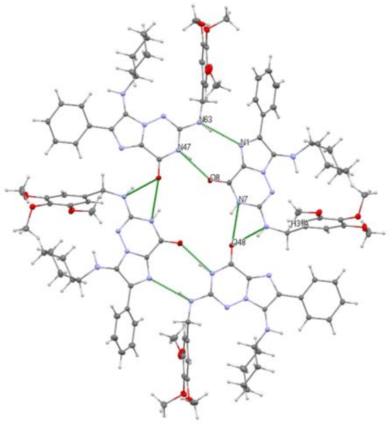

B **C**

Figure S2. Two molecules in the symmetric unit (A) of **5l** crystal structure with the atom labeling scheme. Both molecules exhibit a positional disorder (the less abundant conformation is marked with C-atoms in green). A single molecule adopts the hairpin-like conformation (B). Strong H-bonds motives lead to the formation of tetramers in the crystal lattice (C). Displacement ellipsoids of non-hydrogen atoms are drawn at the 30% probability level. H atoms are presented as small spheres with an arbitrary radius.

The pK predication by ChemAxon software.

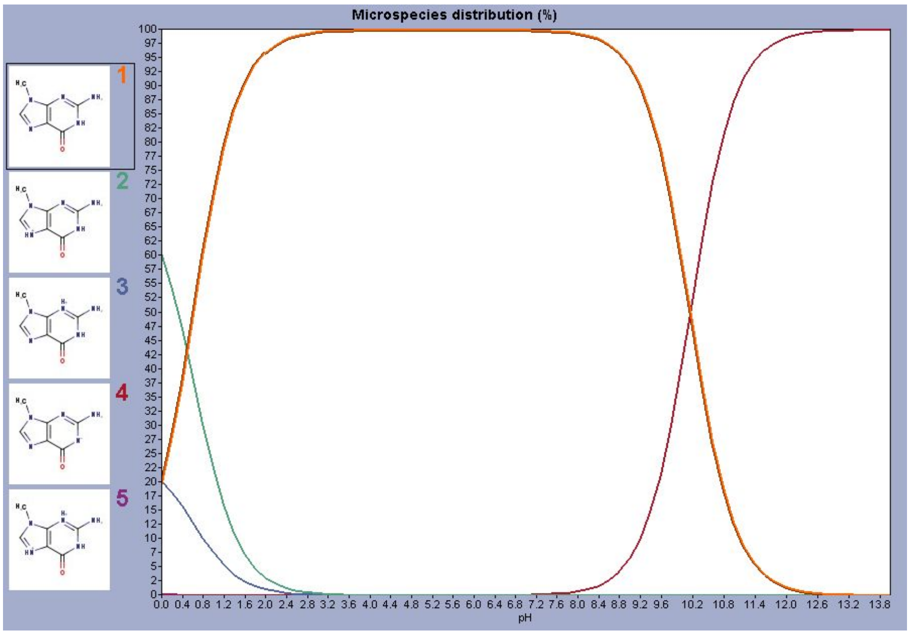

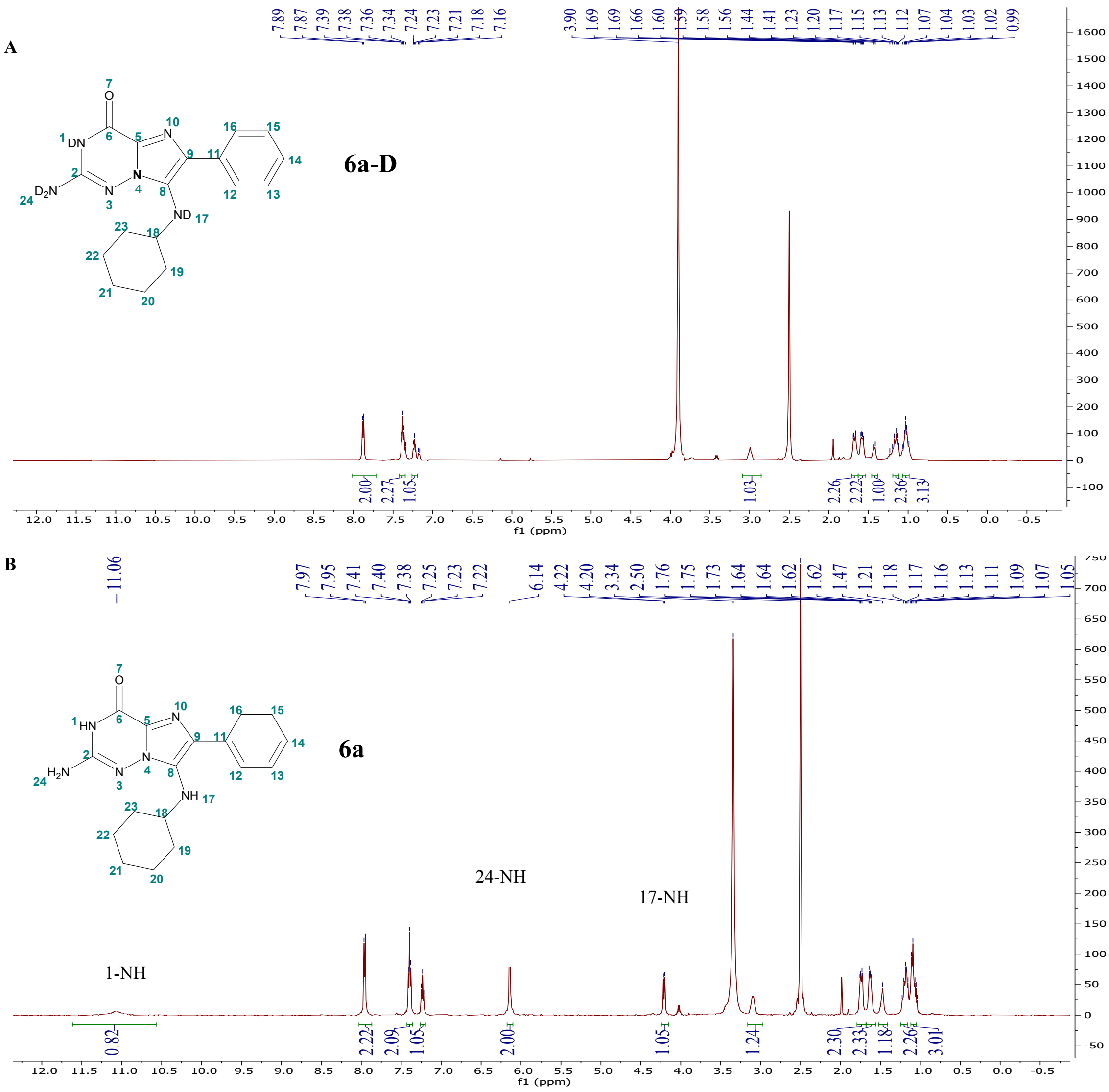

Figure S3. The comparison between [2]D-NMR spectrum of **6a-D** (A, DMSO-d_6 and several drops D_2O) and ^{1}H-NMR spectrum of **6a** (B, DMSO-d_6)

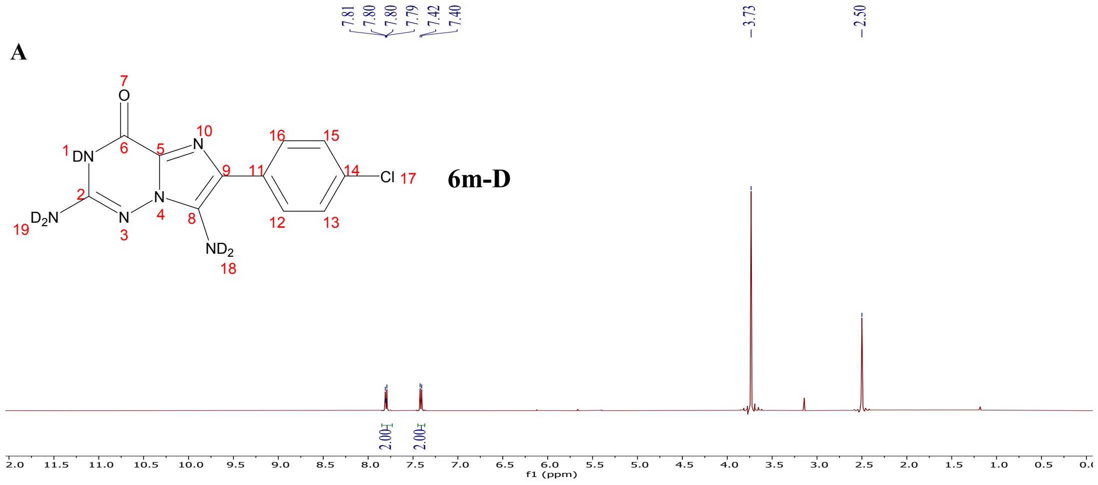

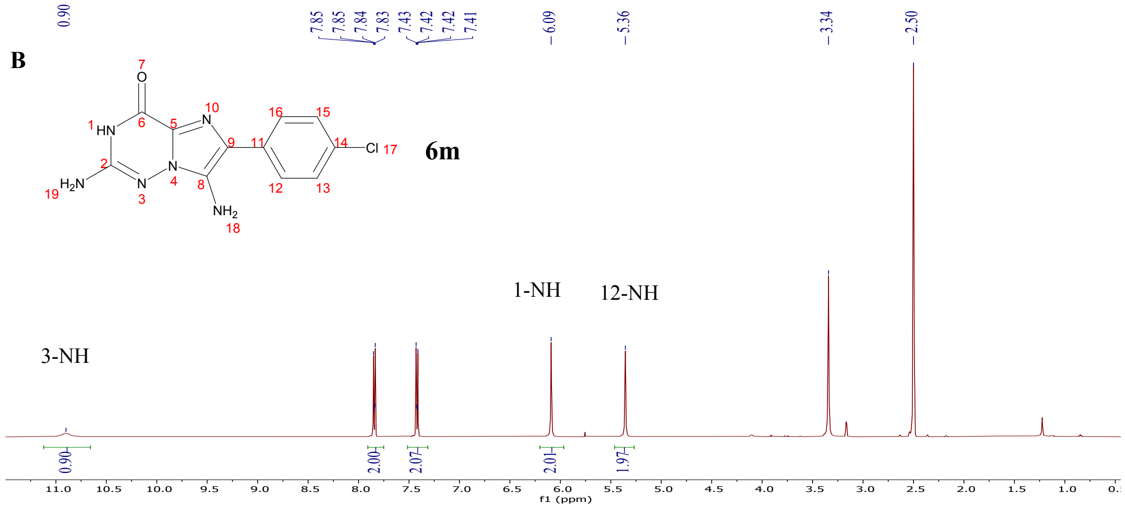

Figure S4: The comparison of between ^{2}D-NMR spectrum of **6m-D** (A, DMSO-d_6 and several drops D_2O) and ^{1}H-NMR spectrum of **6m** (B, DMSO-d_6)

Proposed Mechanism:

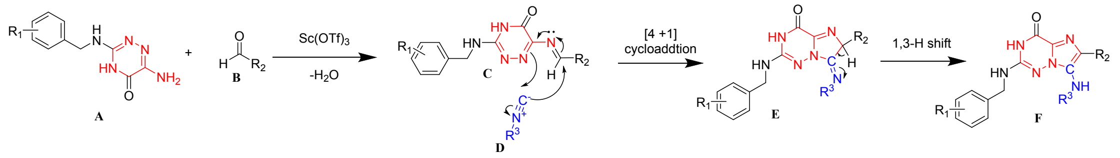

First, 2-aminopyridine (A) reacts with aldehyde (B) in the presence of $Sc(OTf)_3$ to form imine intermediate C, then C reacts with isocyanide (D) though [4 +1] cycloaddition to give E, finally, then aromatization of E via double 1,3-H shift to form the imidazole-fused heterocycle F.

^{1}H NMR spectrum of **4a** (500 MHz, DMSO-d_6)

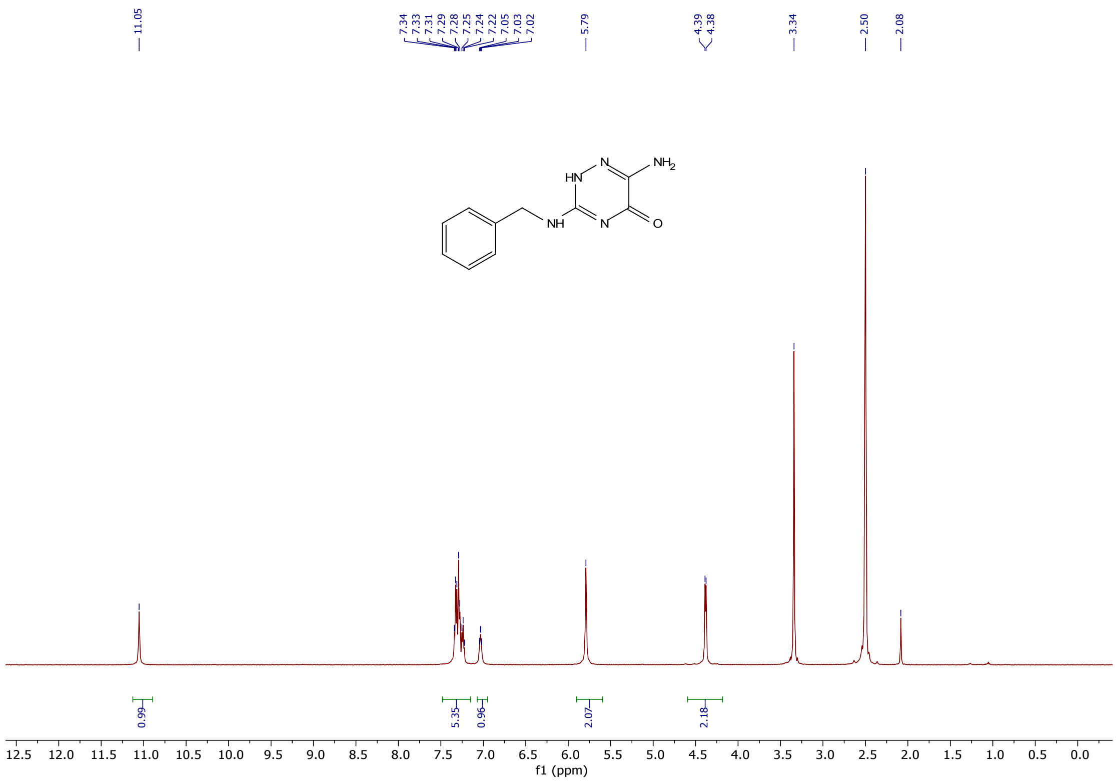

$^{13}C\{^{1}H\}$ NMR spectrum of **4a** (126 MHz, DMSO-d_6)

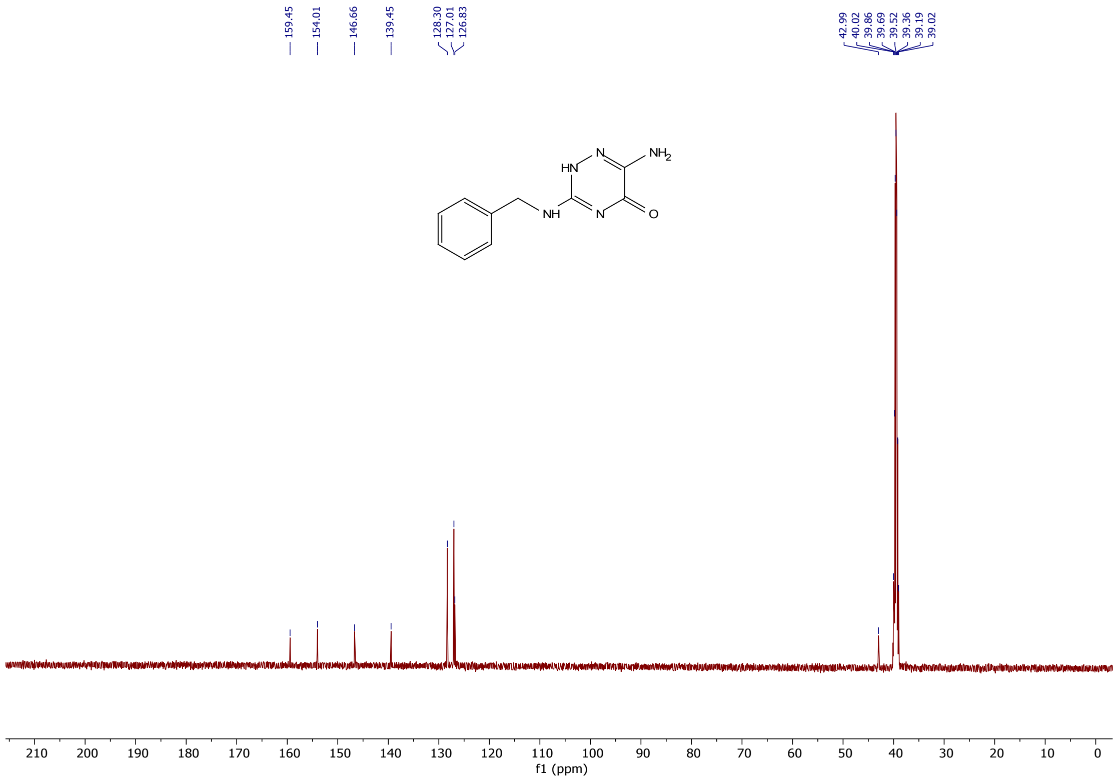

^{1}H NMR spectrum of **4b** (500 MHz, DMSO-d_6)

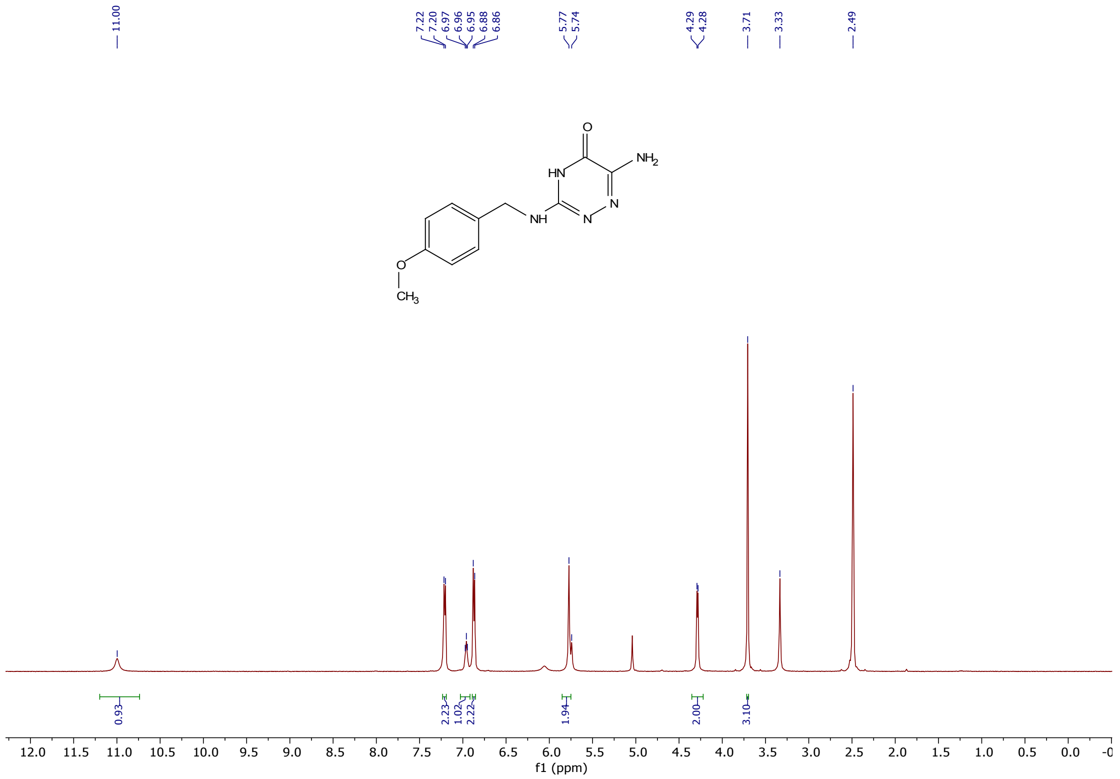

^{13}C{^{1}H} NMR spectrum of **4b** (126 MHz, DMSO-d_6)

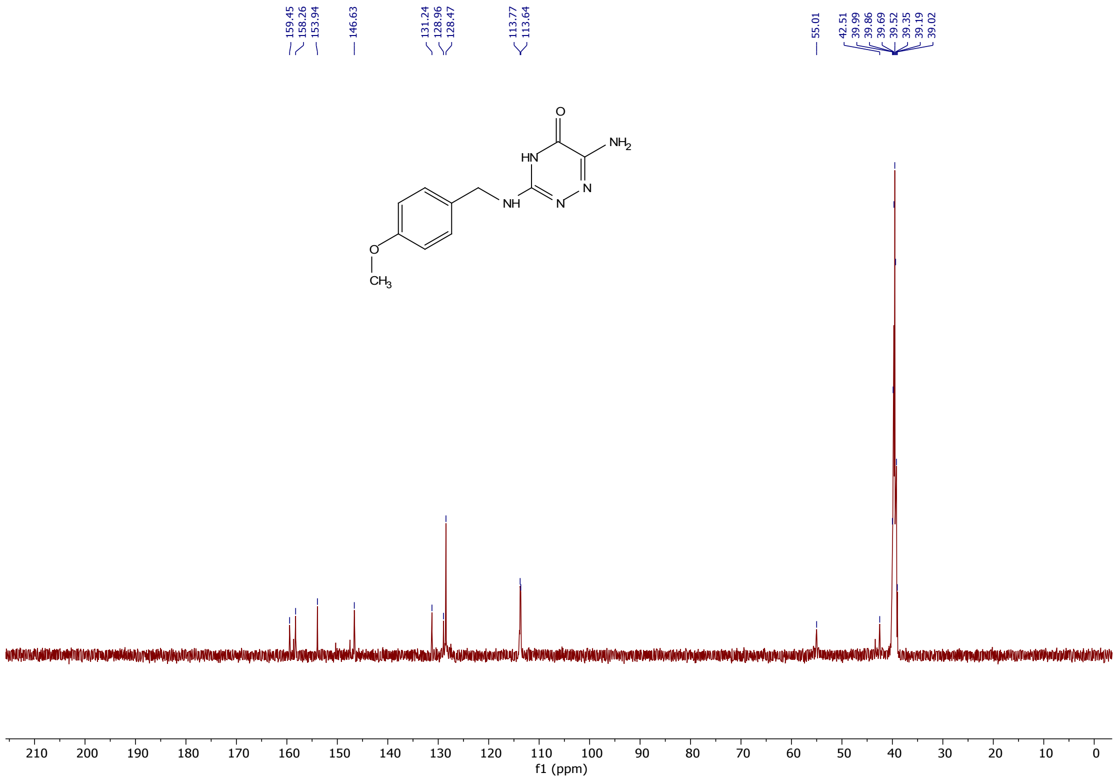

^{1}H NMR spectrum of **4c** (500 MHz, DMSO-d_6)

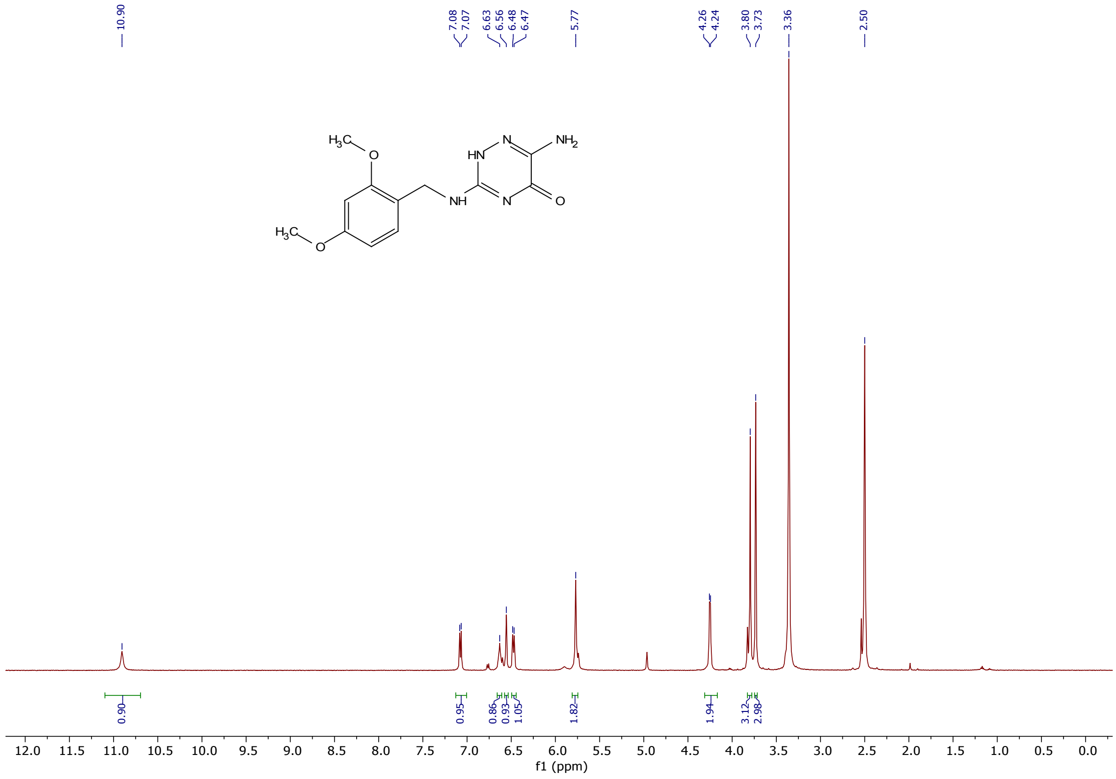

^{13}C{^{1}H} NMR spectrum of **4c** (126 MHz, DMSO-d_6)

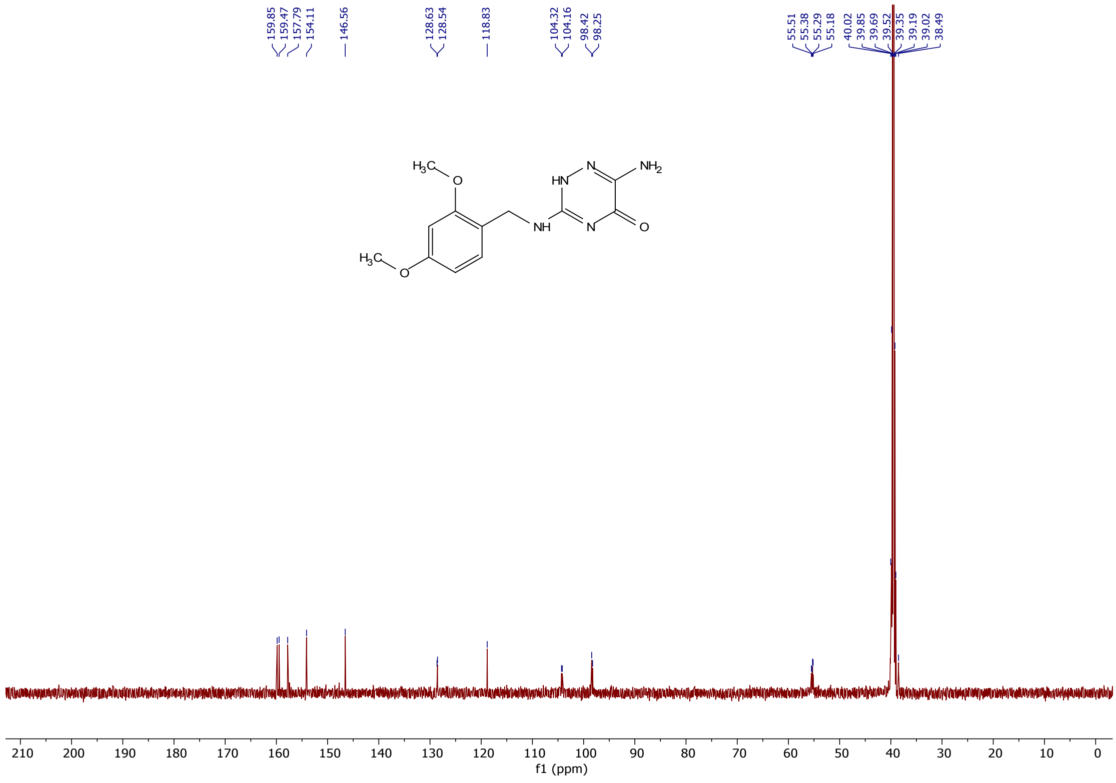

^{1}H NMR spectrum of **4d** (500 MHz, DMSO-d_6)

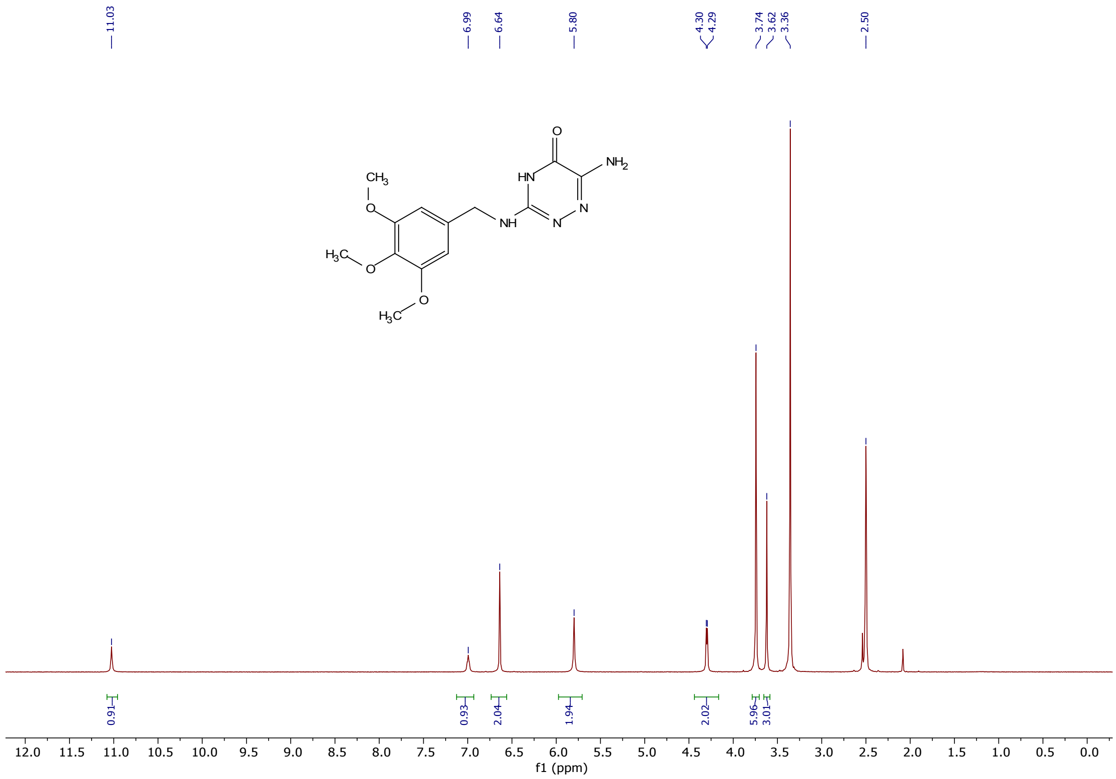

$^{13}C\{^{1}H\}$ NMR spectrum of **4d** (126 MHz, DMSO-d_6)

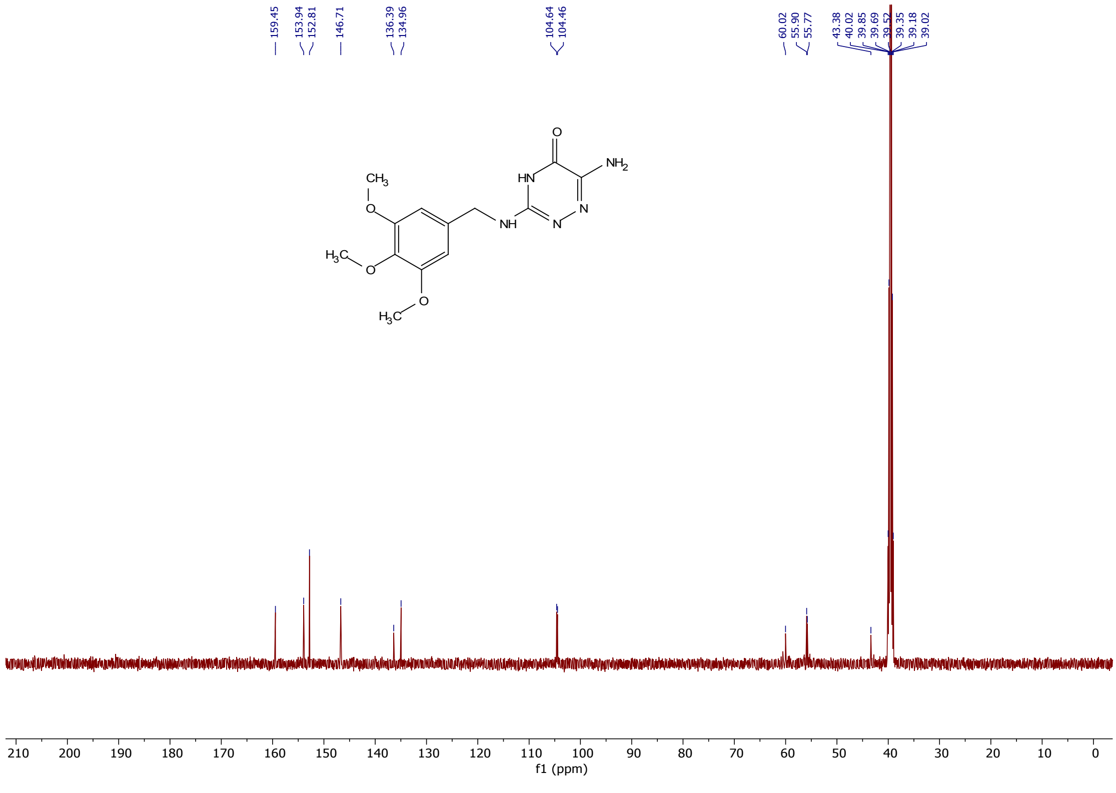

^{1}H NMR spectrum of **5a** (500 MHz, DMSO-d_6)

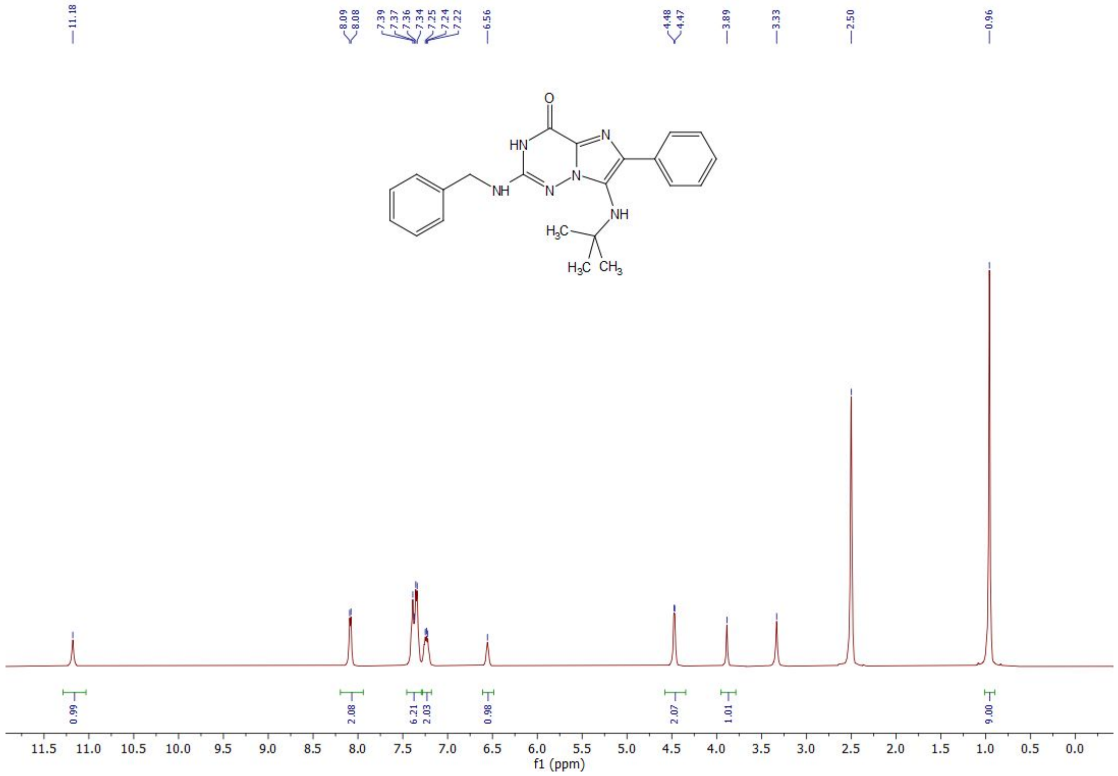

$^{13}C\{^{1}H\}$ NMR spectrum of **5a** (126 MHz, DMSO-d_6)

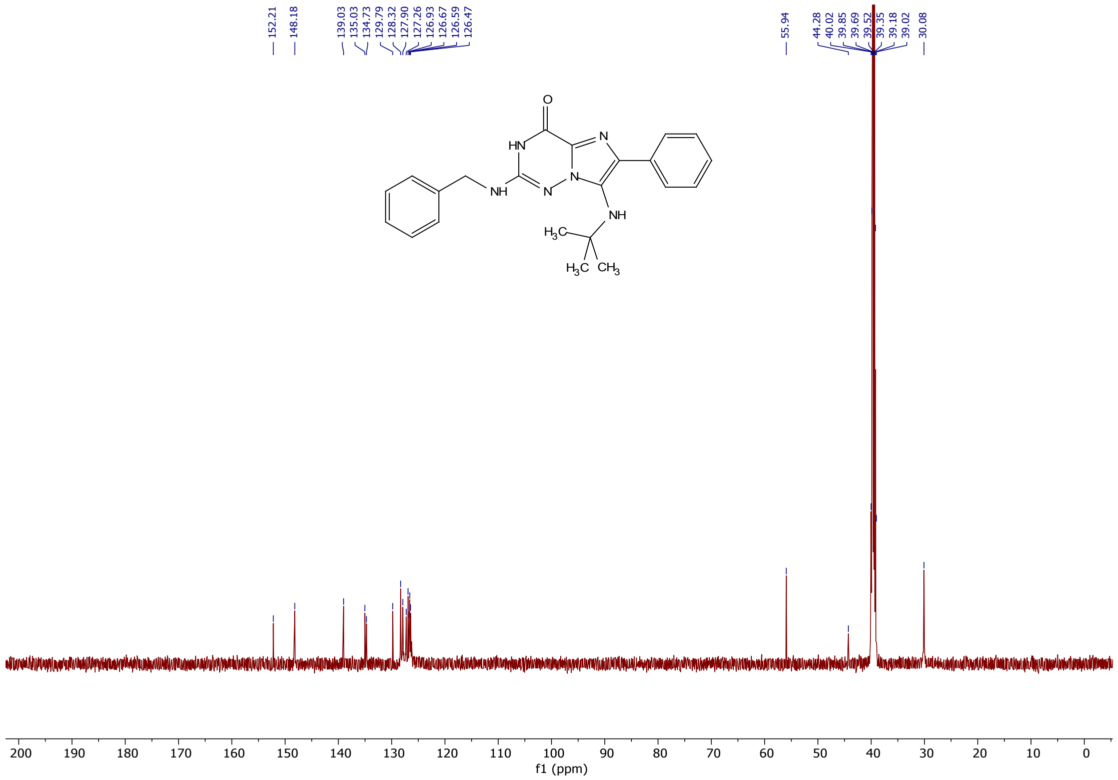

^{1}H NMR spectrum of **5b** (500 MHz, DMSO-d_6)

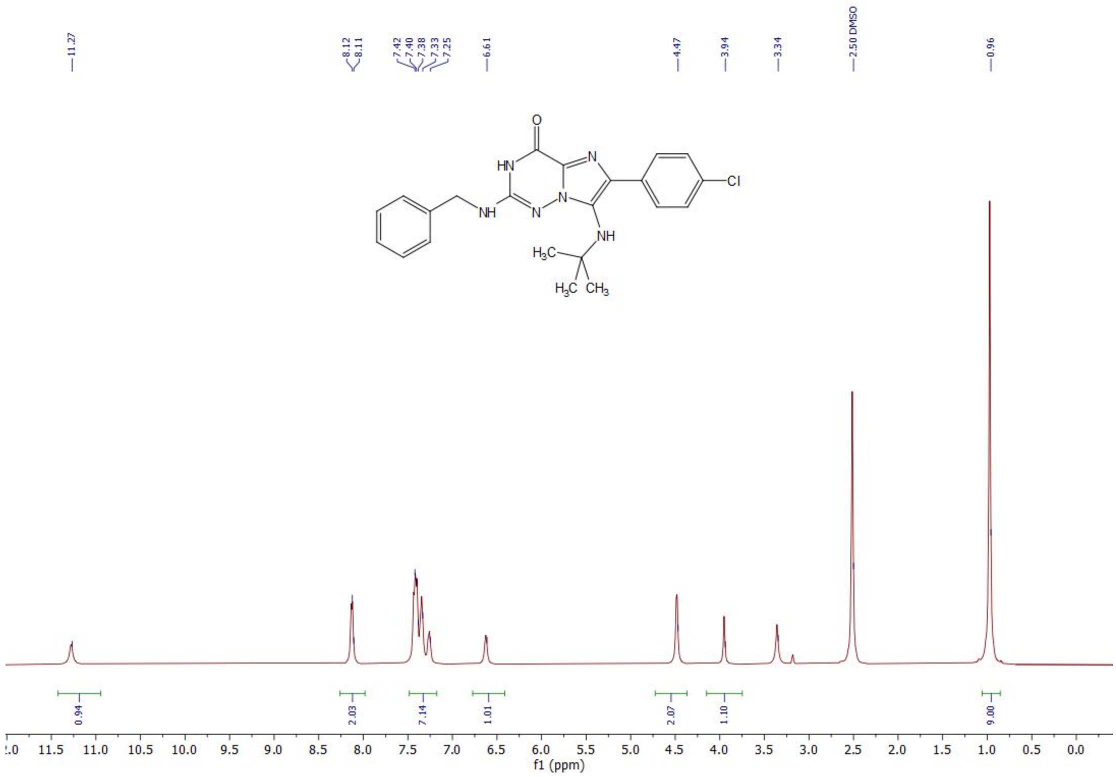

$^{13}C\{^{1}H\}$ NMR spectrum of **5b** (126 MHz, DMSO-d_6)

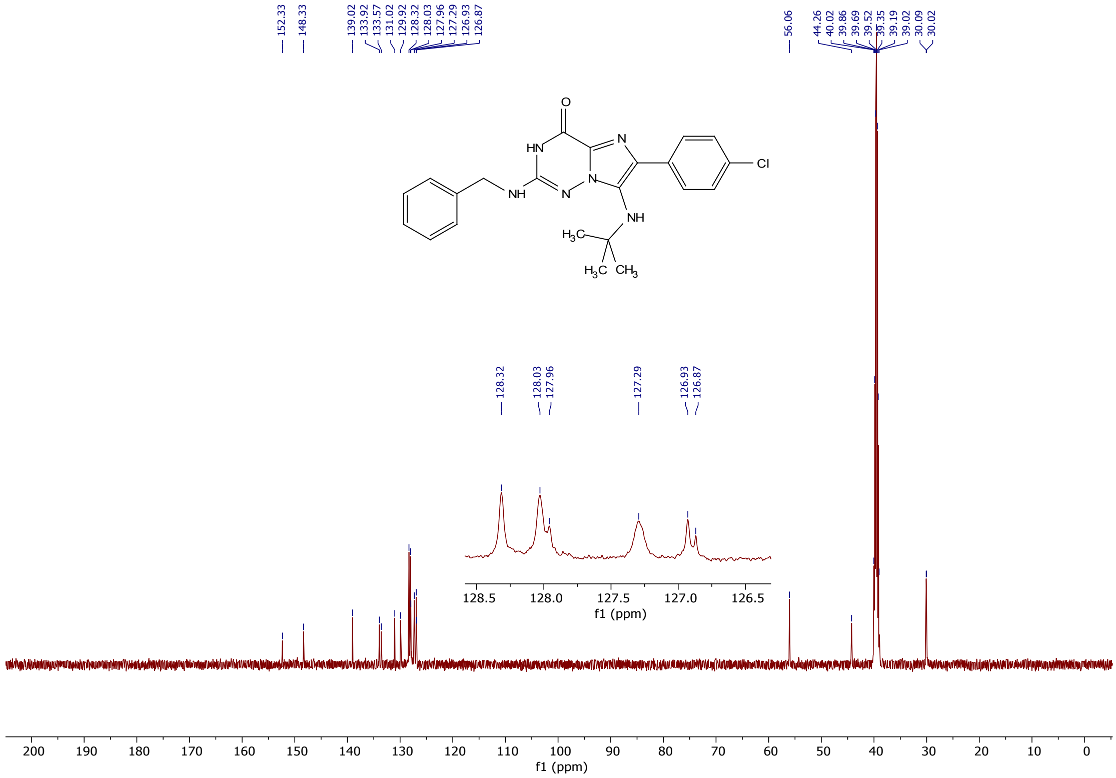

^{1}H NMR spectrum of **5c** (500 MHz, DMSO-d$_6$)

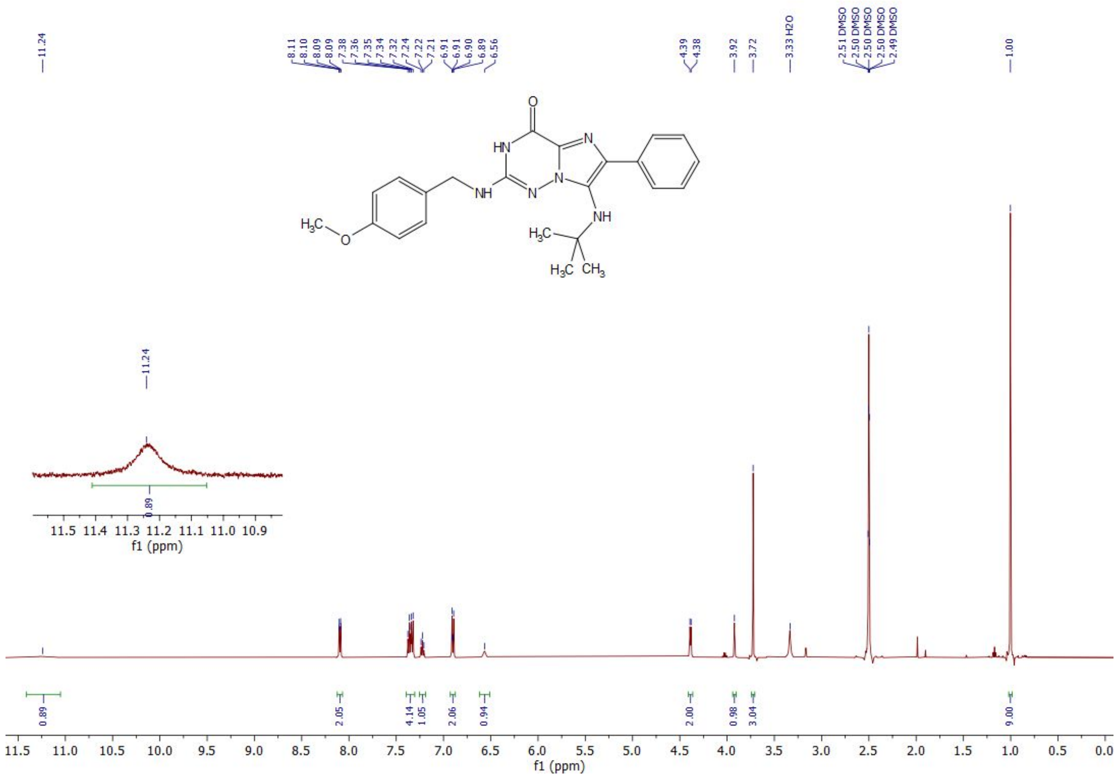

^{13}C{^{1}H} NMR spectrum of **5c** (126 MHz, DMSO-d$_6$)

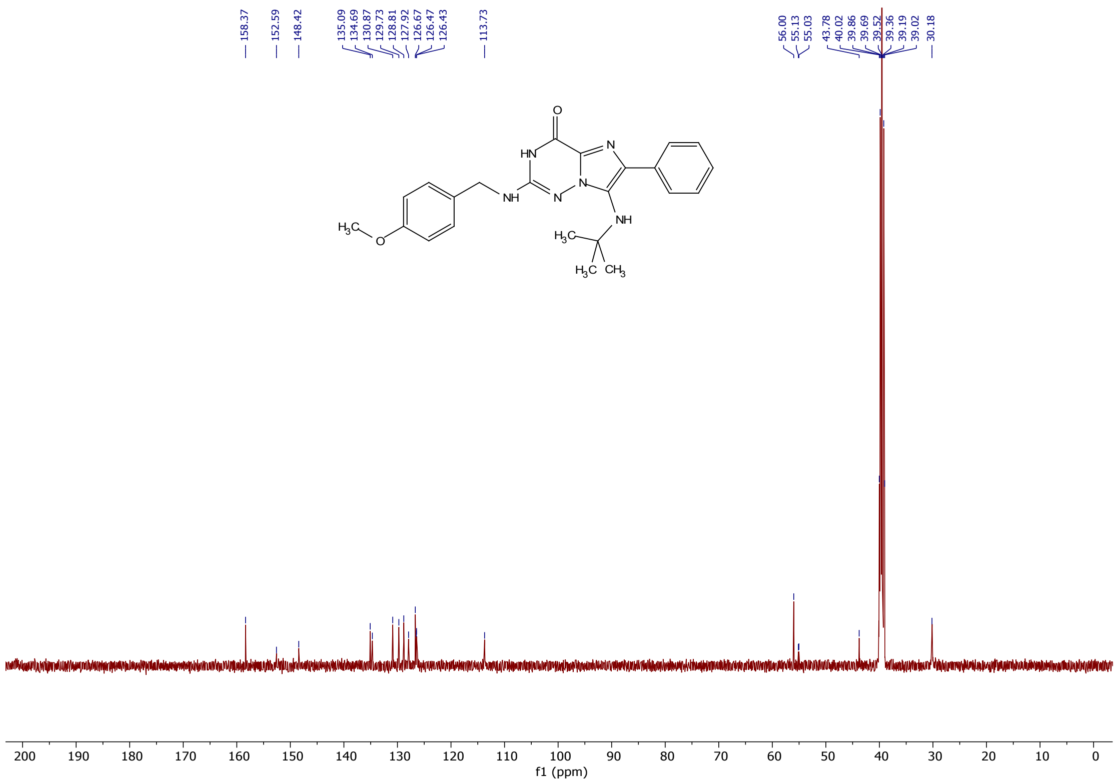

^{1}H NMR spectrum of **5d** (500 MHz, DMSO-d$_6$)

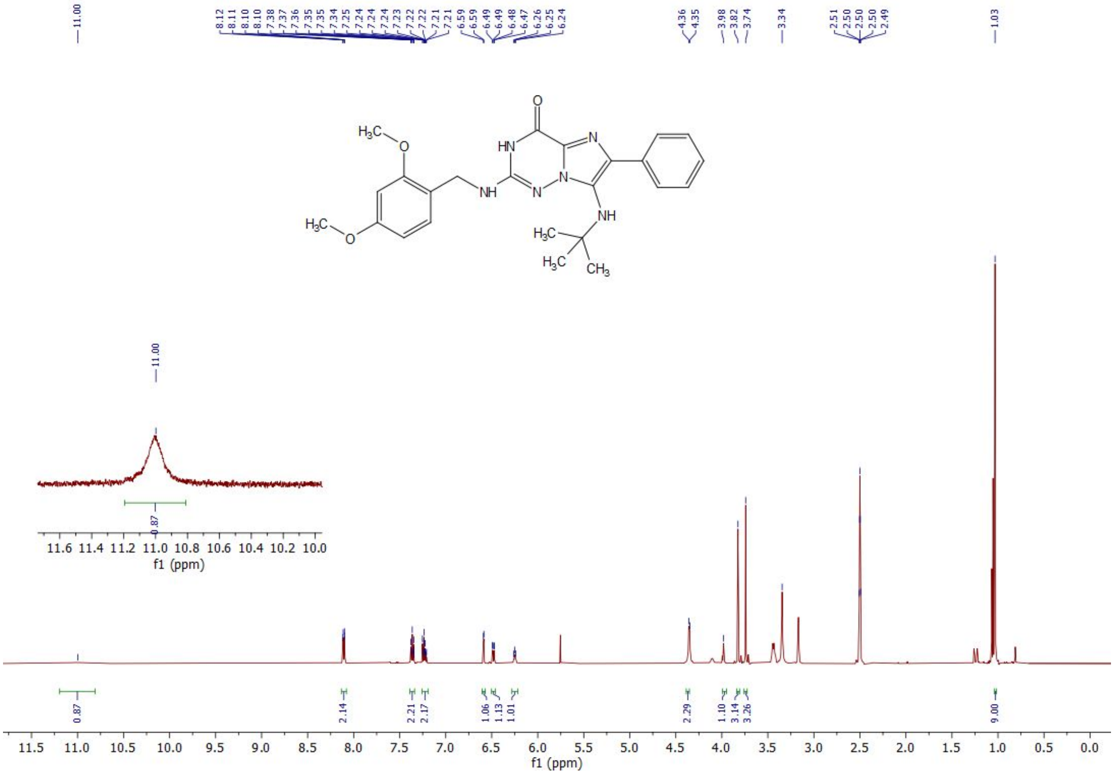

$^{13}C\{^{1}H\}$ NMR spectrum of **5d** (126 MHz, DMSO-d$_6$)

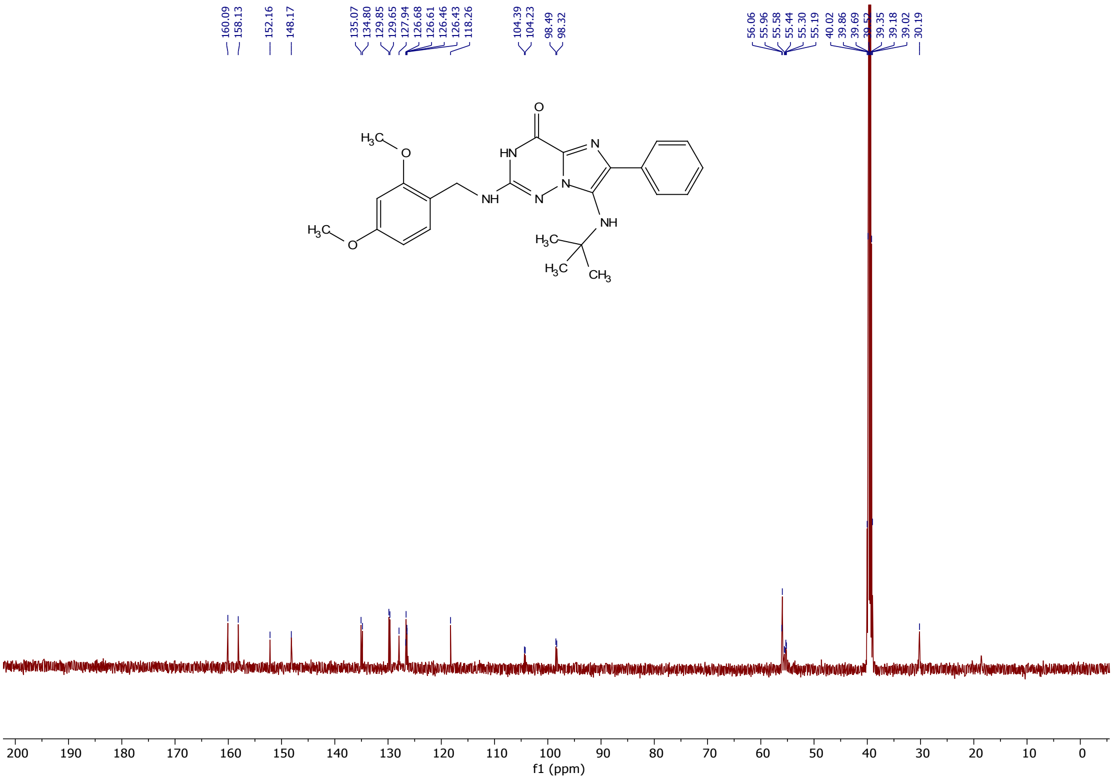

^{1}H NMR spectrum of **5e**(500 MHz, DMSO-d$_6$)

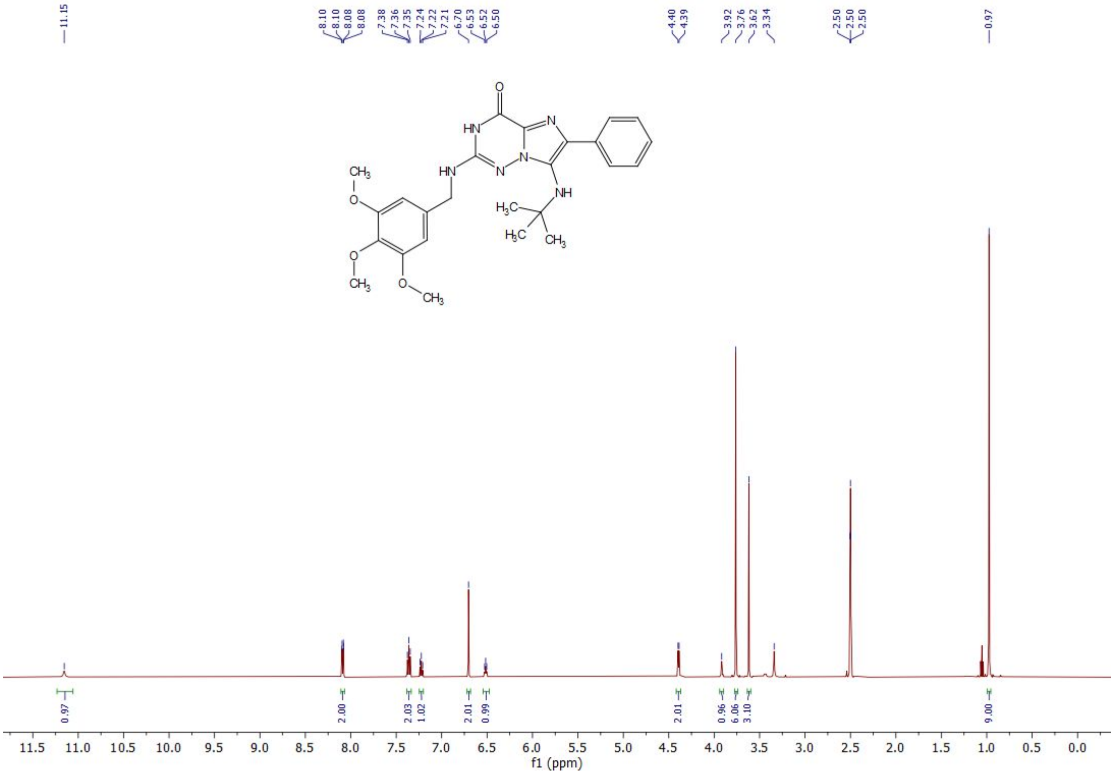

^{13}C{^{1}H} NMR spectrum of **5e** (126 MHz, DMSO-d$_6$)

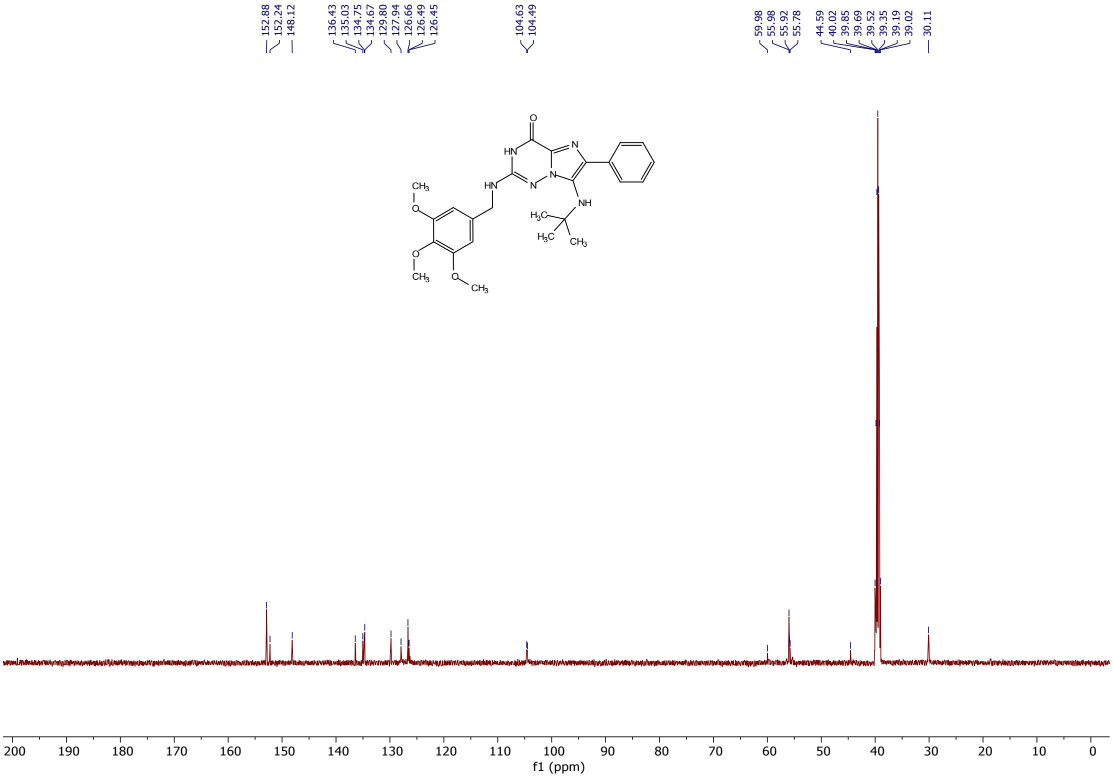

^{1}H NMR spectrum of **5f** (500 MHz, DMSO-d_6)

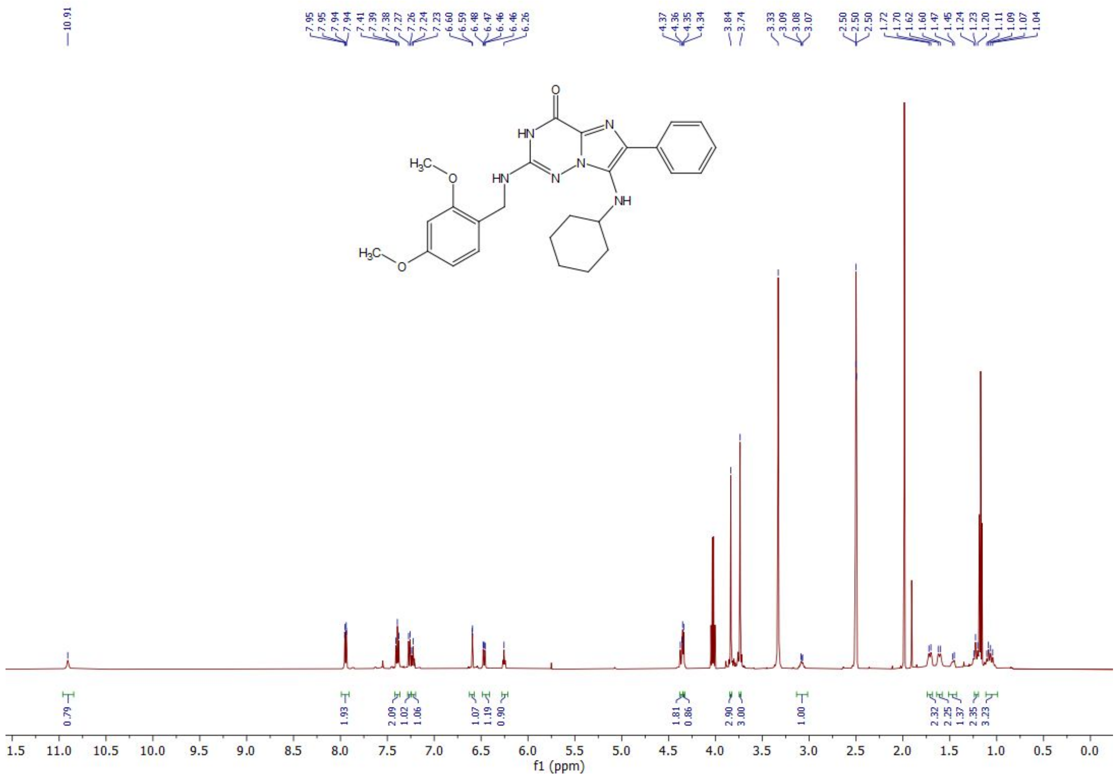

$^{13}C\{^{1}H\}$ NMR spectrum of **5f** (126 MHz, DMSO-d_6)

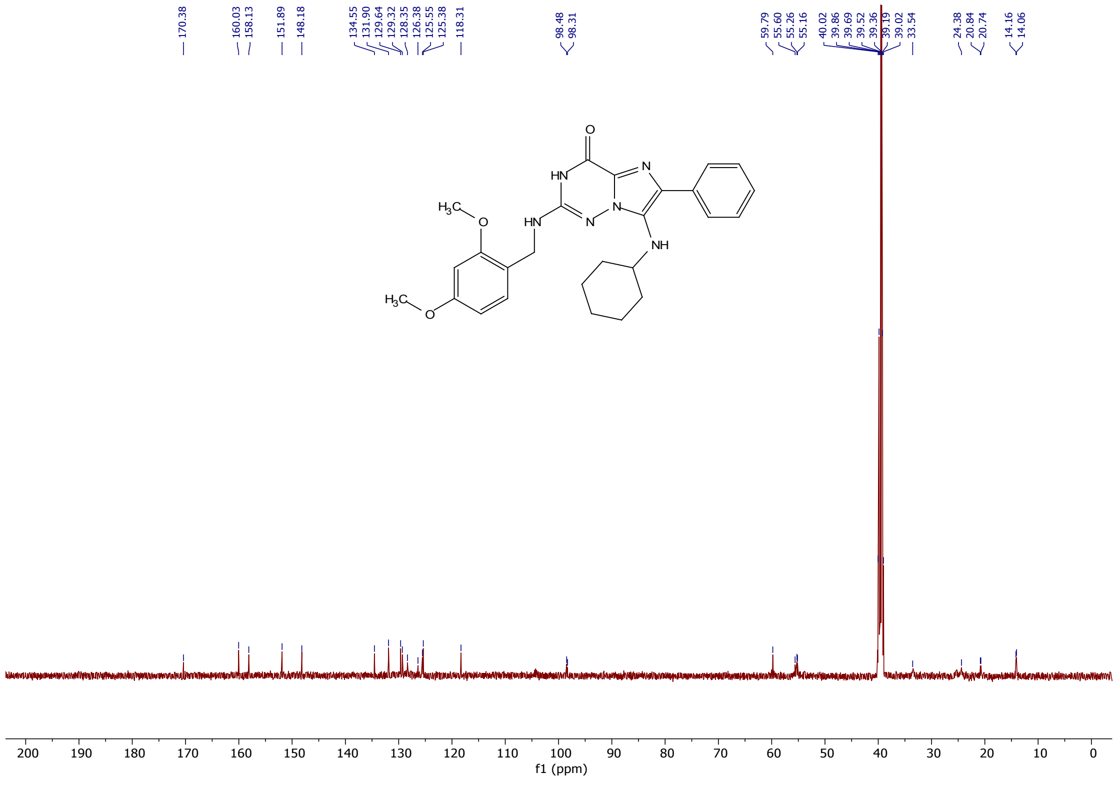

^{1}H NMR spectrum of **5g** (500 MHz, DMSO-d$_6$)

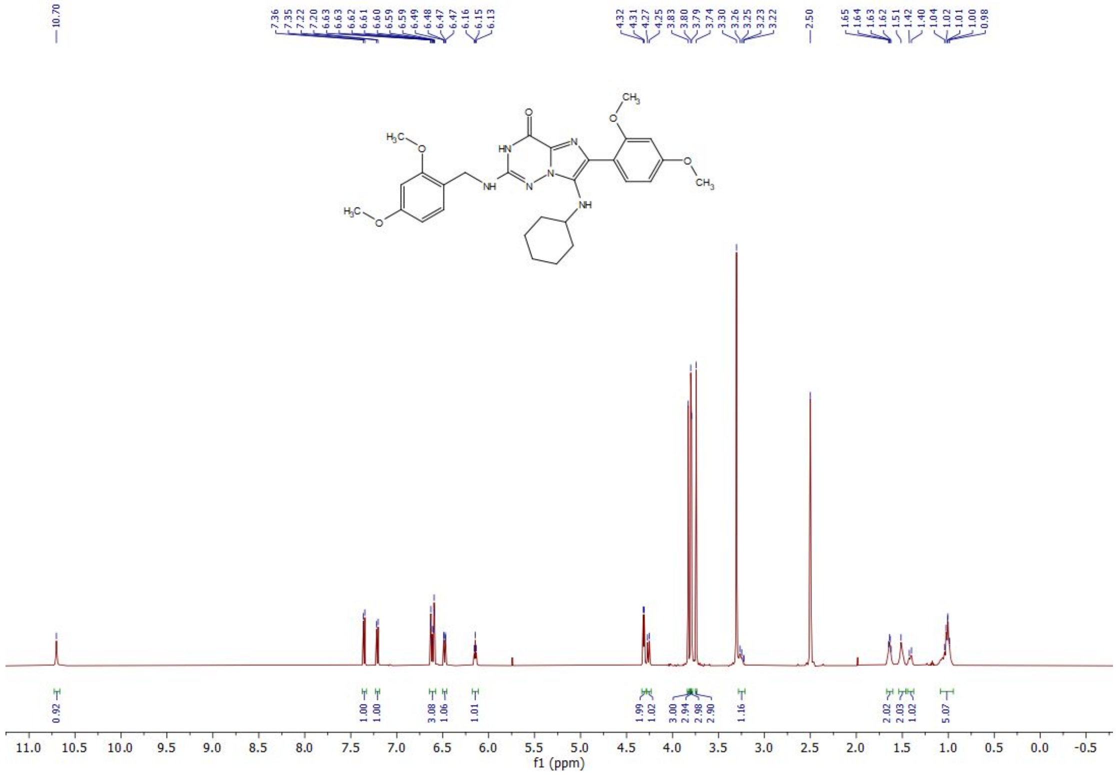

^{13}C{^{1}H} NMR spectrum of **5g** (126 MHz, DMSO-d$_6$)

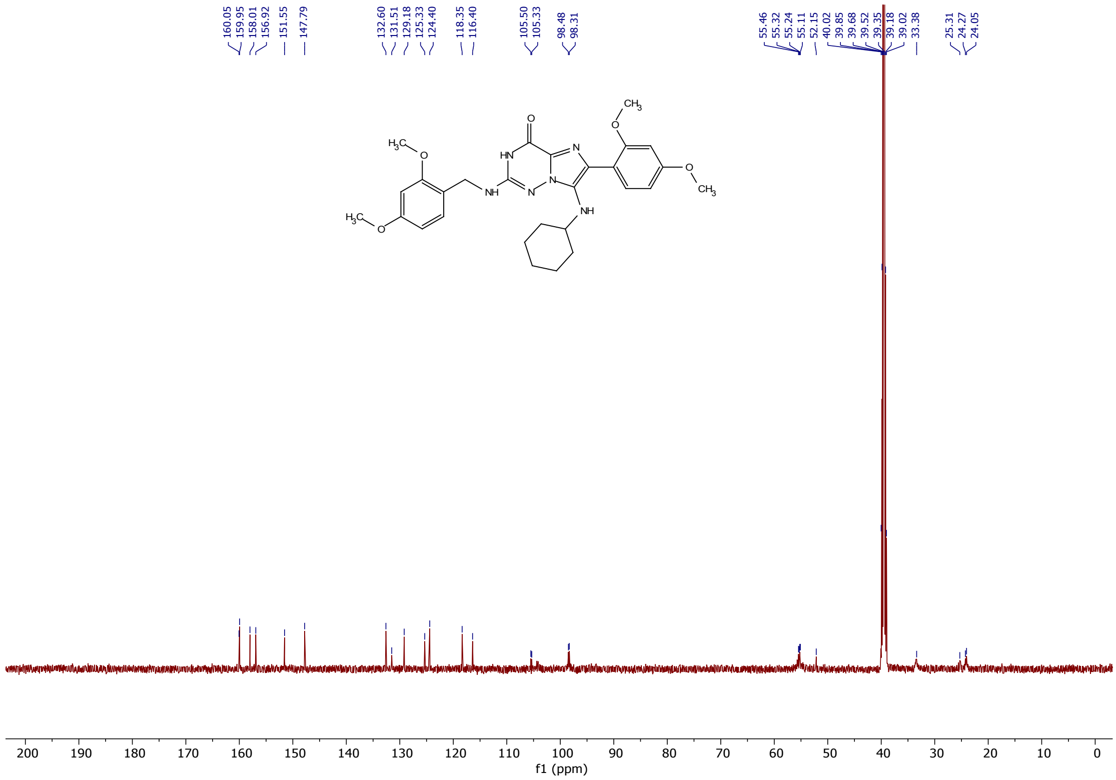

^{1}H NMR spectrum of **5h** (500 MHz, DMSO-d$_6$)

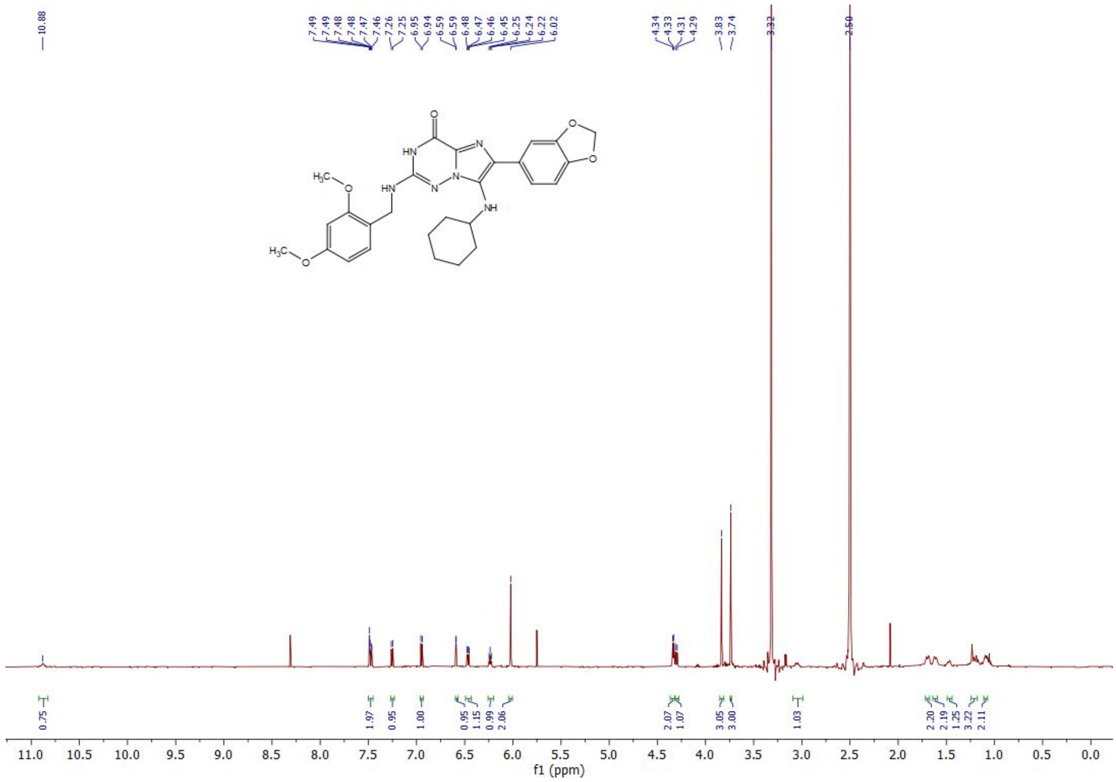

^{13}C{^{1}H} NMR spectrum of **5h** (126 MHz, DMSO-d$_6$)

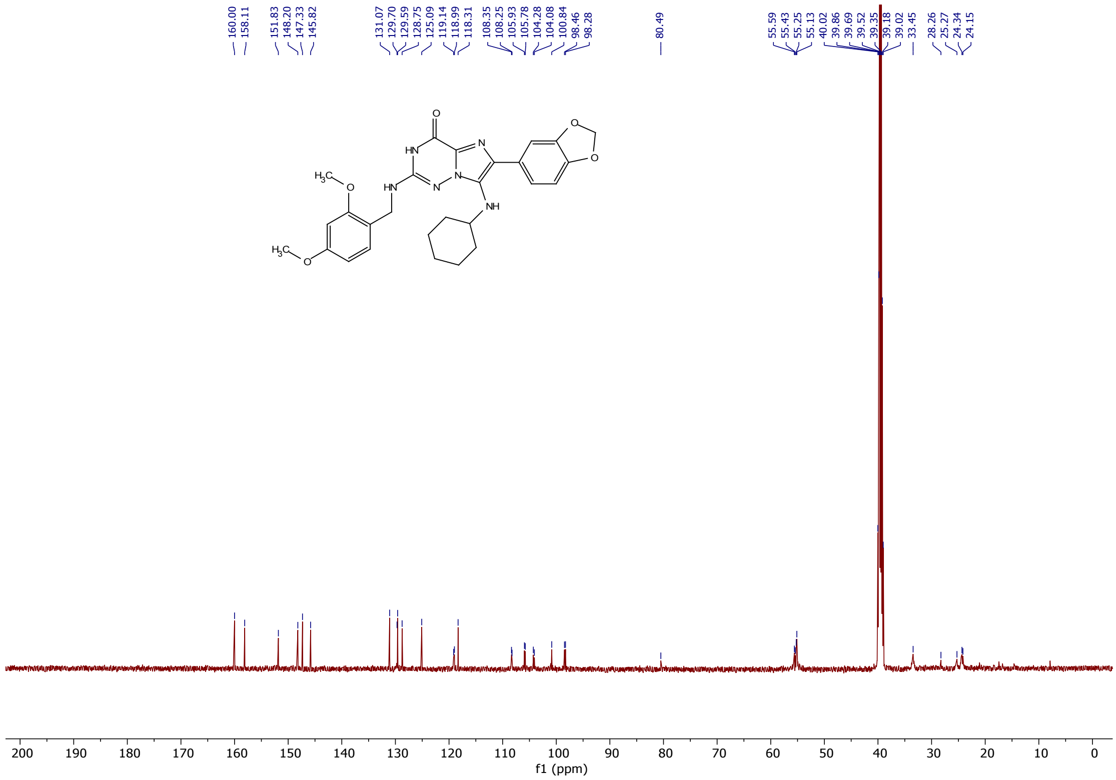

^{1}H NMR spectrum of **5i** (500 MHz, DMSO-d_6)

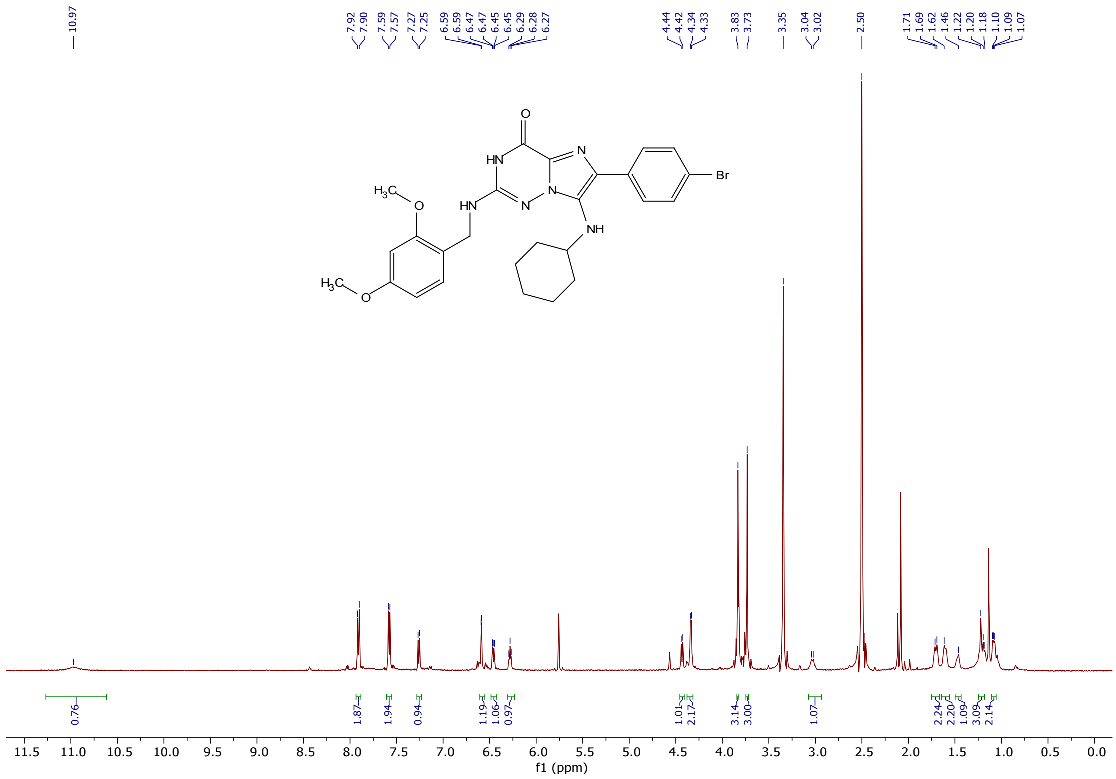

^{13}C{^{1}H} NMR spectrum of **5i** (126 MHz, DMSO-d_6)

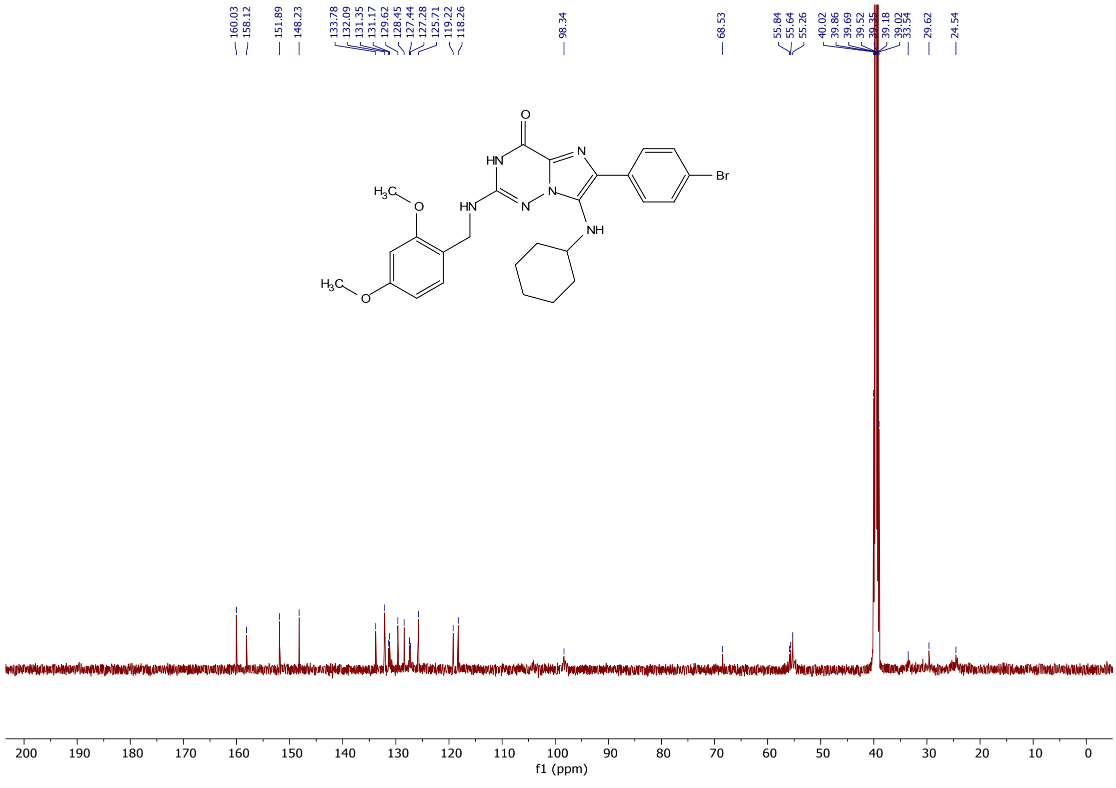

^{1}H NMR spectrum of **5j** (500 MHz, DMSO-d_6)

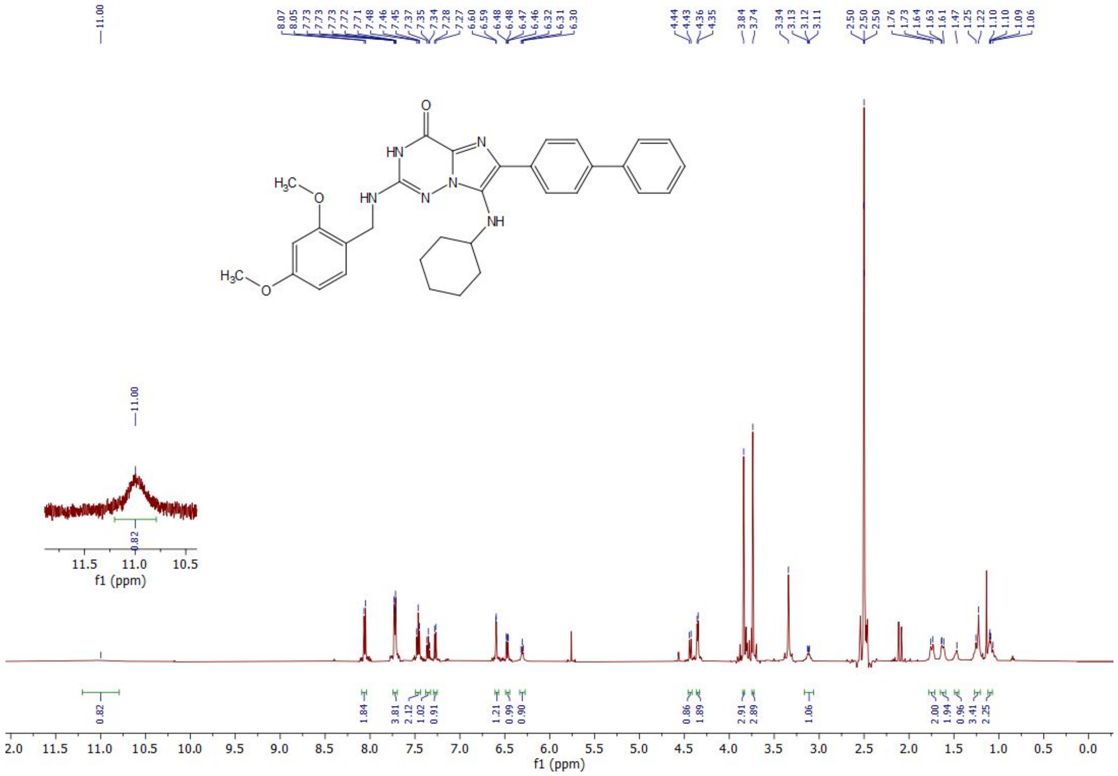

$^{13}C\{^{1}H\}$ NMR spectrum of **5j** (126 MHz, DMSO-d_6)

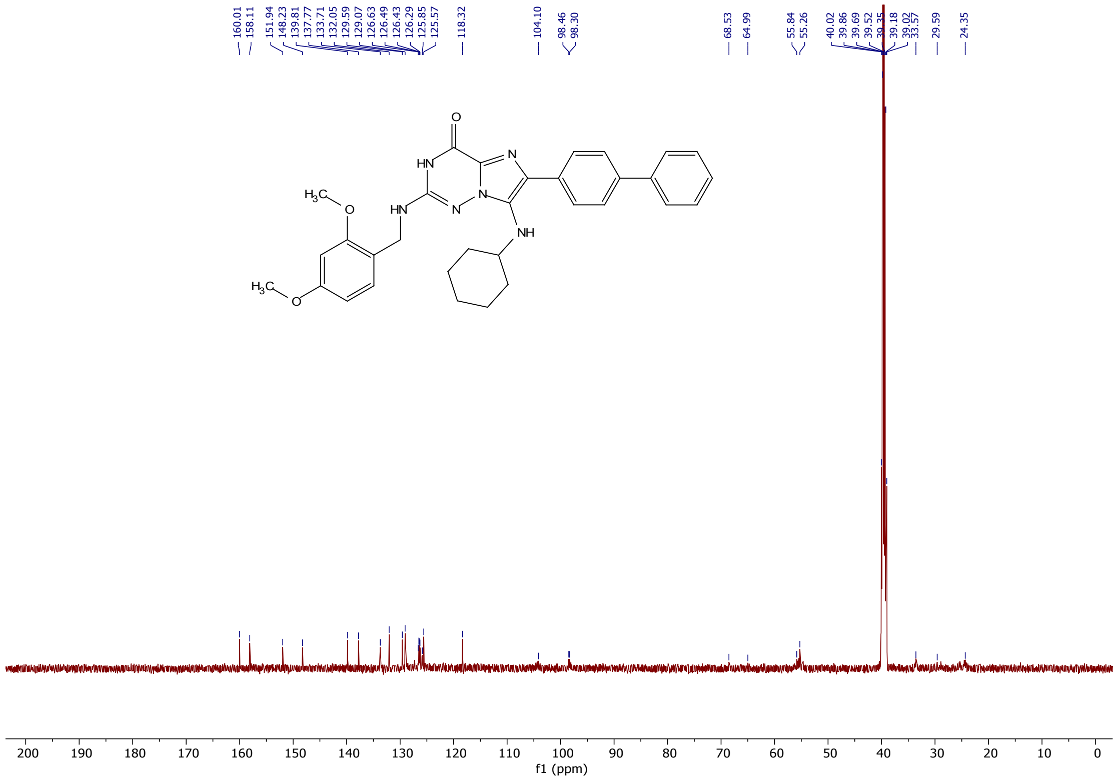

^{1}H NMR spectrum of **5k** (500 MHz, DMSO-d_6)

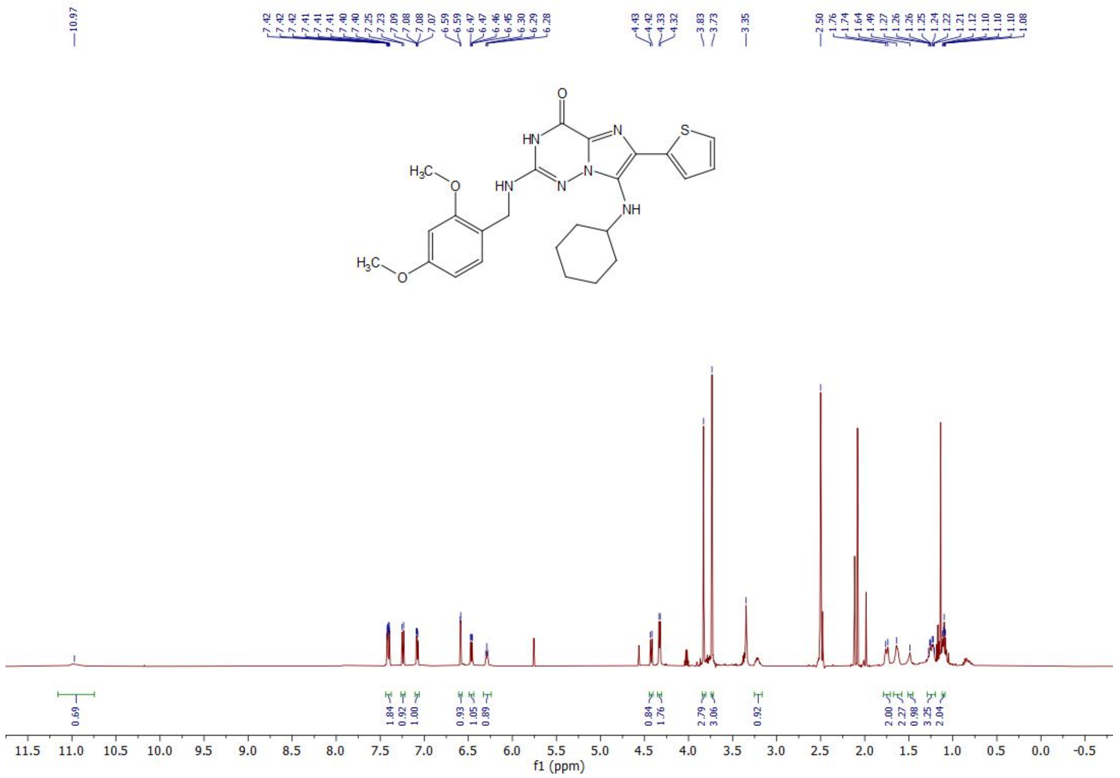

$^{13}C\{^{1}H\}$ NMR spectrum of **5k** (126 MHz, DMSO-d_6)

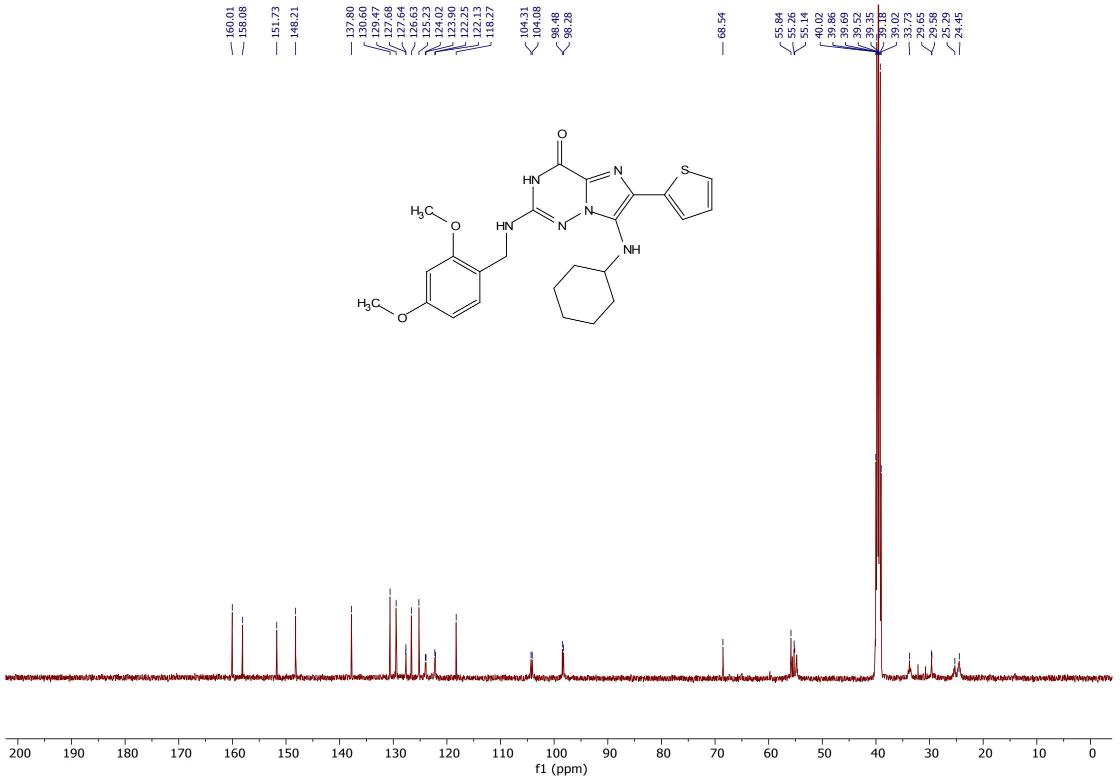

^{1}H NMR spectrum of **5l** (500 MHz, DMSO-d_6)

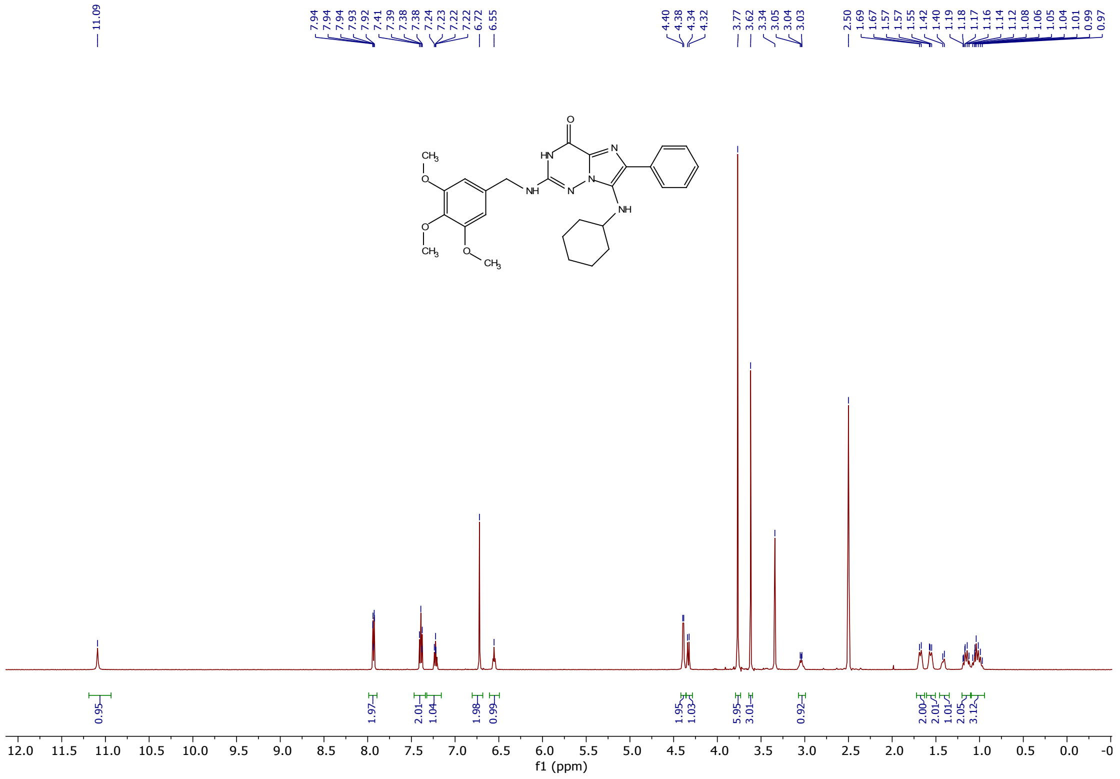

$^{13}C\{^{1}H\}$ NMR spectrum of **5l** (126 MHz, DMSO-d_6)

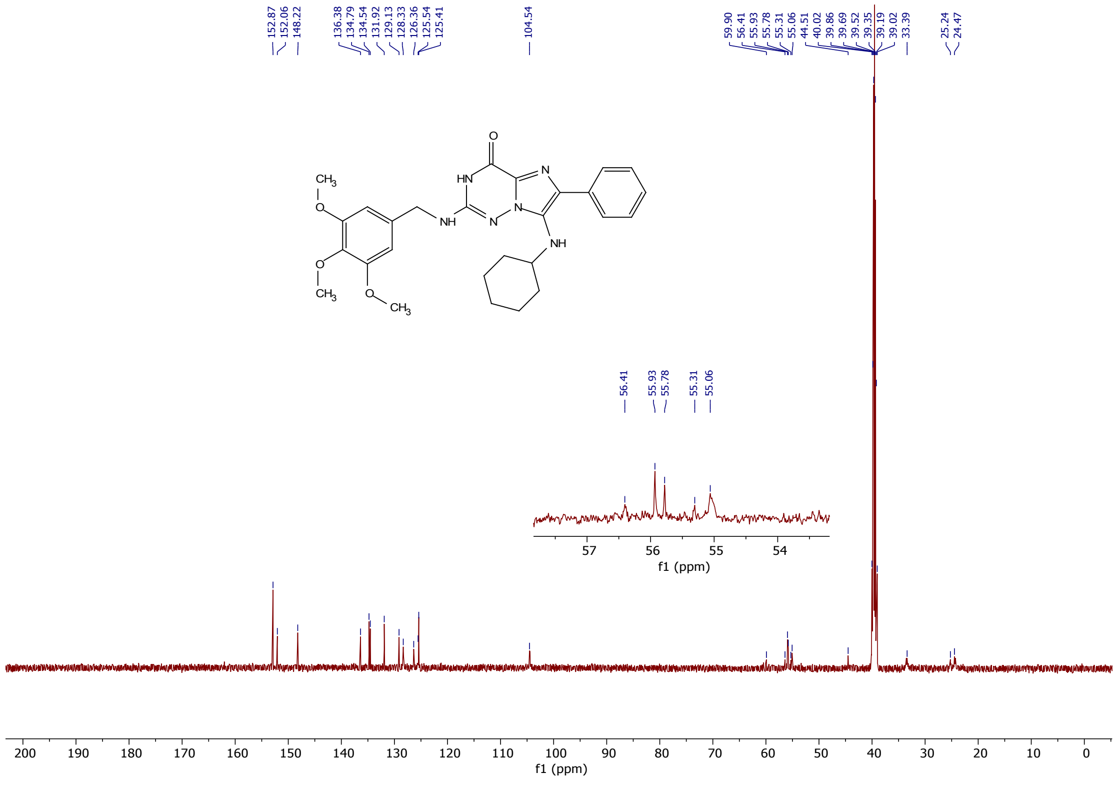

^{1}H NMR spectrum of **5m** (500 MHz, DMSO-d_6)

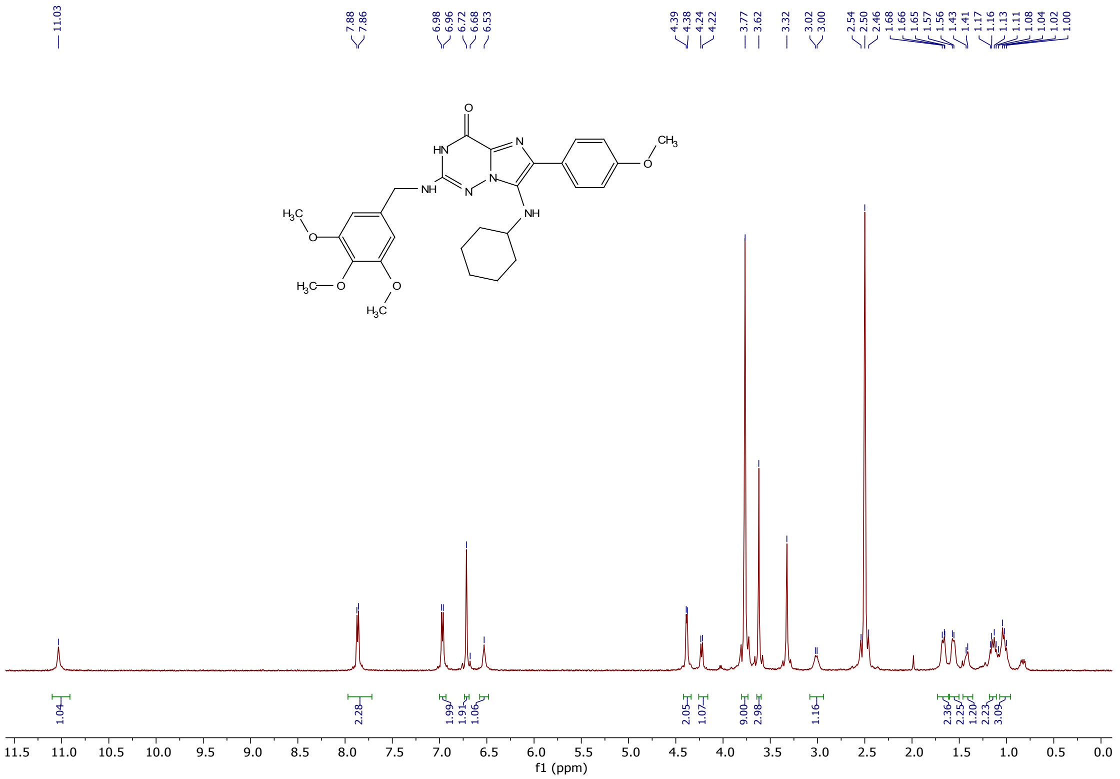

^{13}C{^{1}H} NMR spectrum of **5m** (126 MHz, DMSO-d_6)

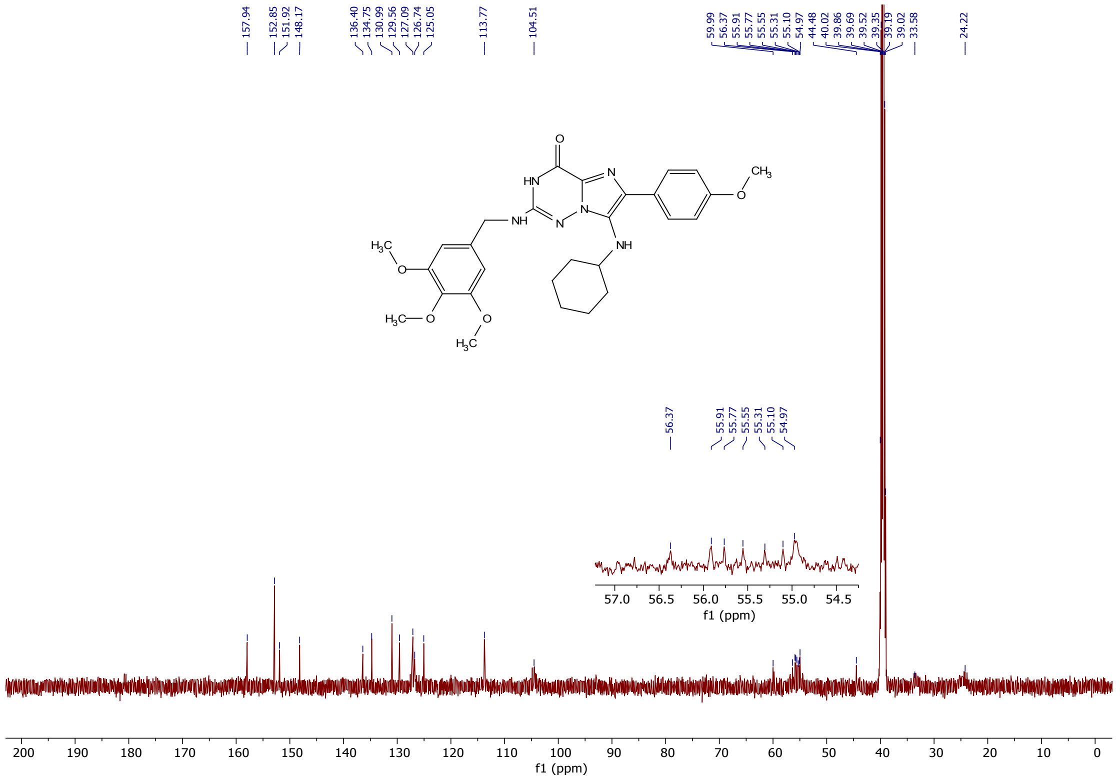

^{1}H NMR spectrum of **5n** (500 MHz, DMSO-d_6)

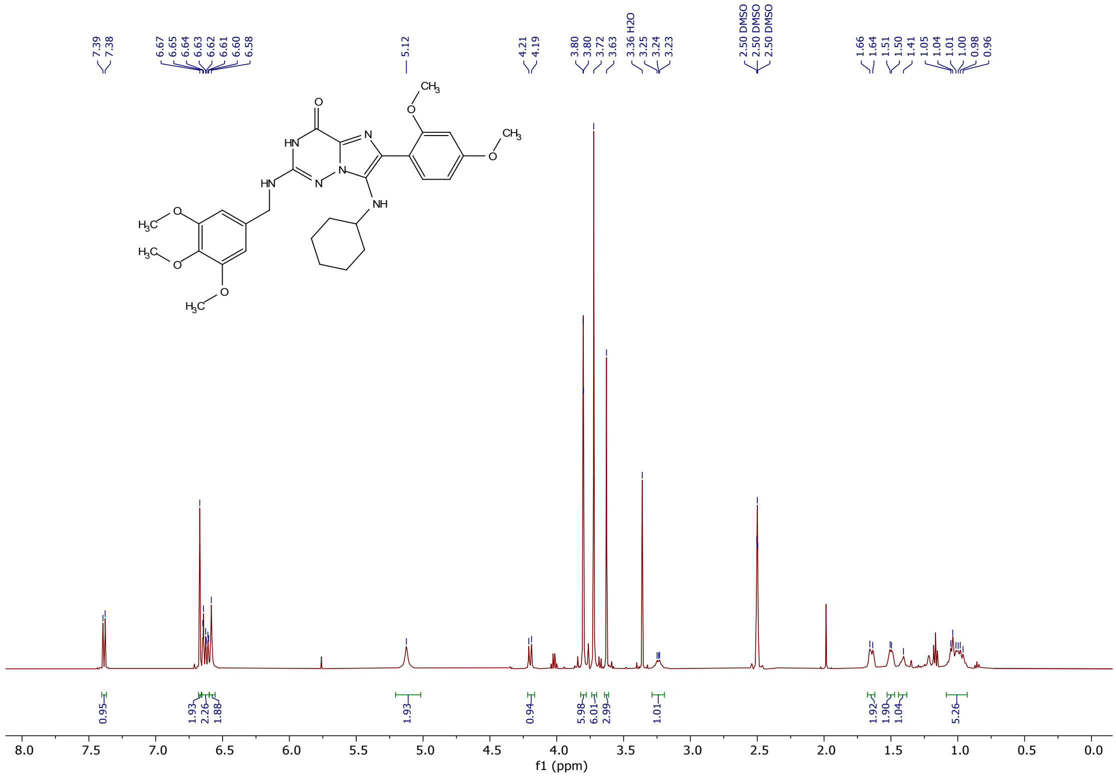

^{13}C{^{1}H} NMR spectrum of **5n** (126 MHz, DMSO-d_6)

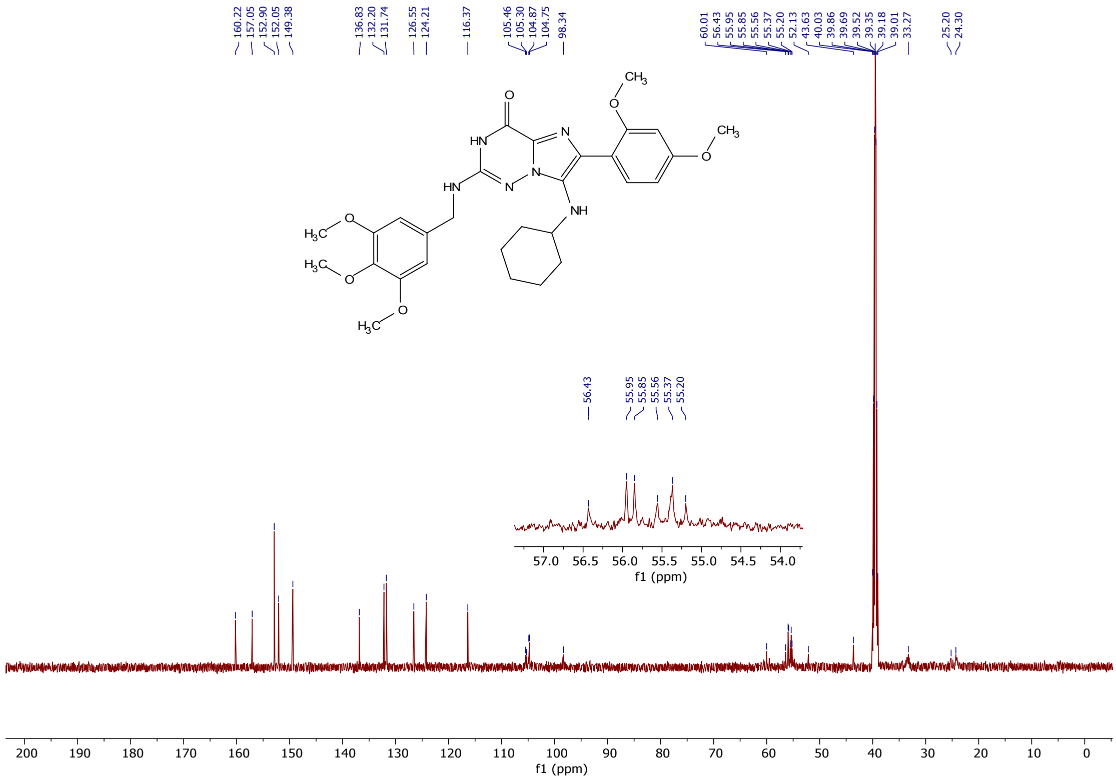

^{1}H NMR spectrum of **5o** (500 MHz, DMSO-d_6)

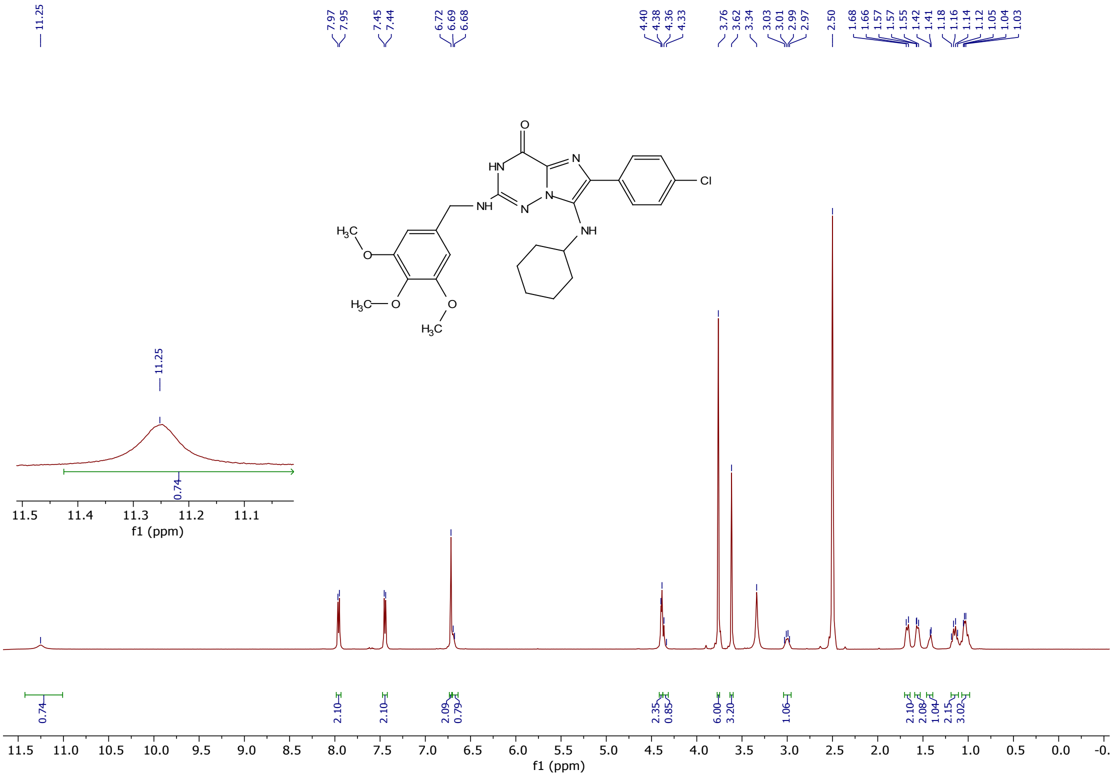

$^{13}C\{^{1}H\}$ NMR spectrum of **5o** (126 MHz, DMSO-d_6)

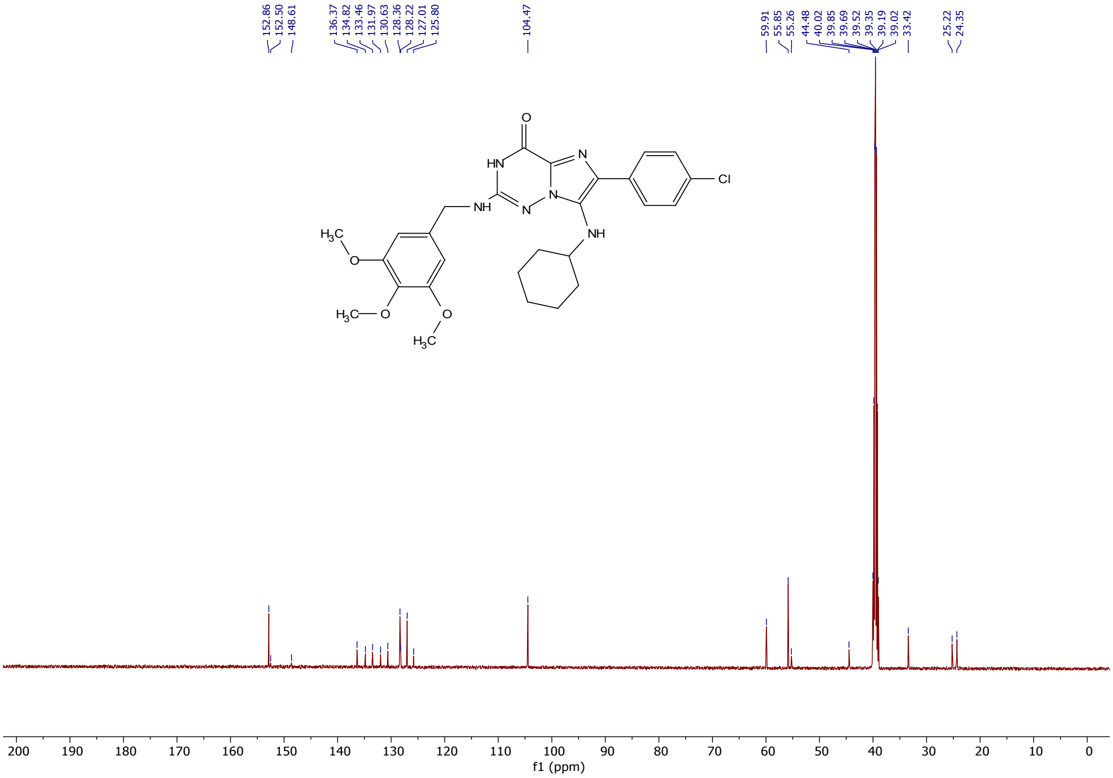

^{1}H NMR spectrum of **5p** (500 MHz, DMSO-d$_6$)

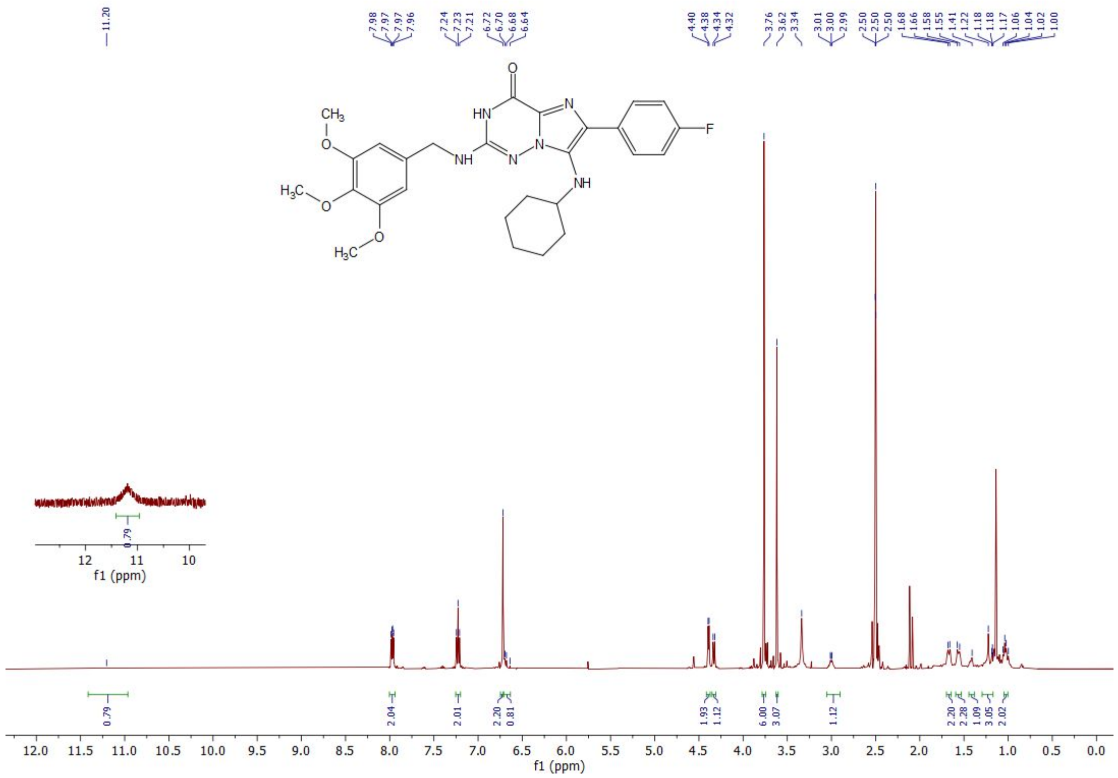

$^{13}C\{^{1}H\}$ NMR spectrum of **5p** (126 MHz, DMSO-d$_6$)

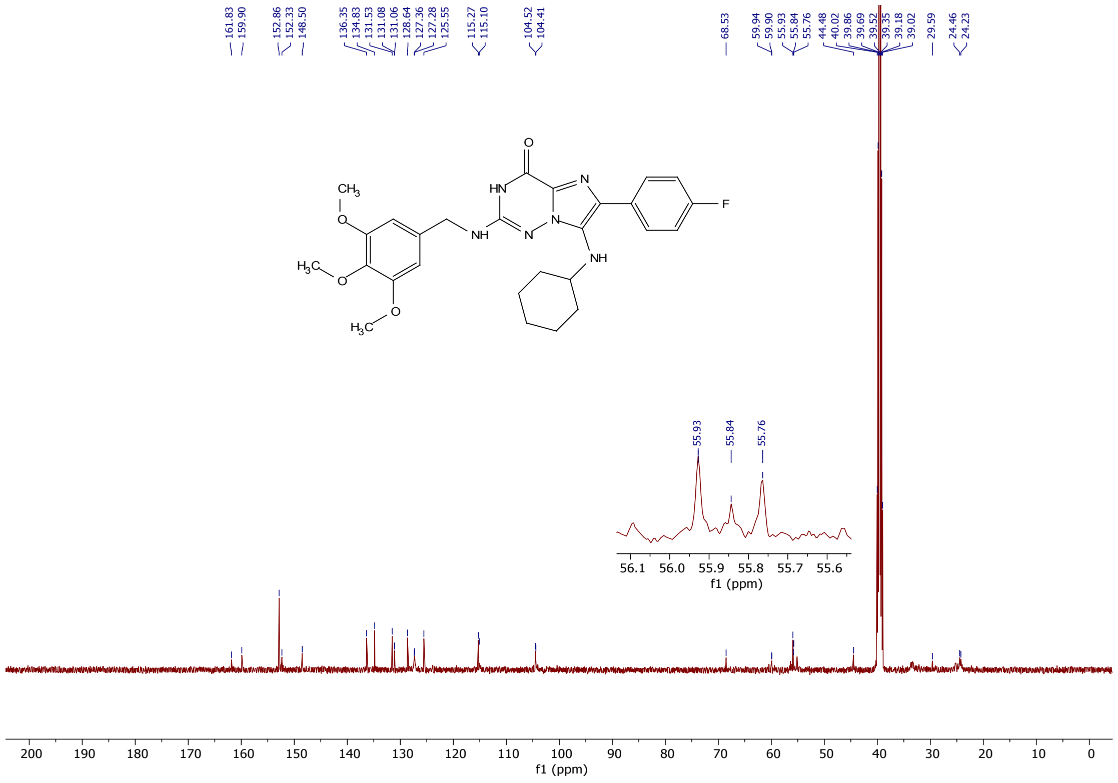

^{1}H NMR spectrum of **5q** (500 MHz, DMSO-d_6)

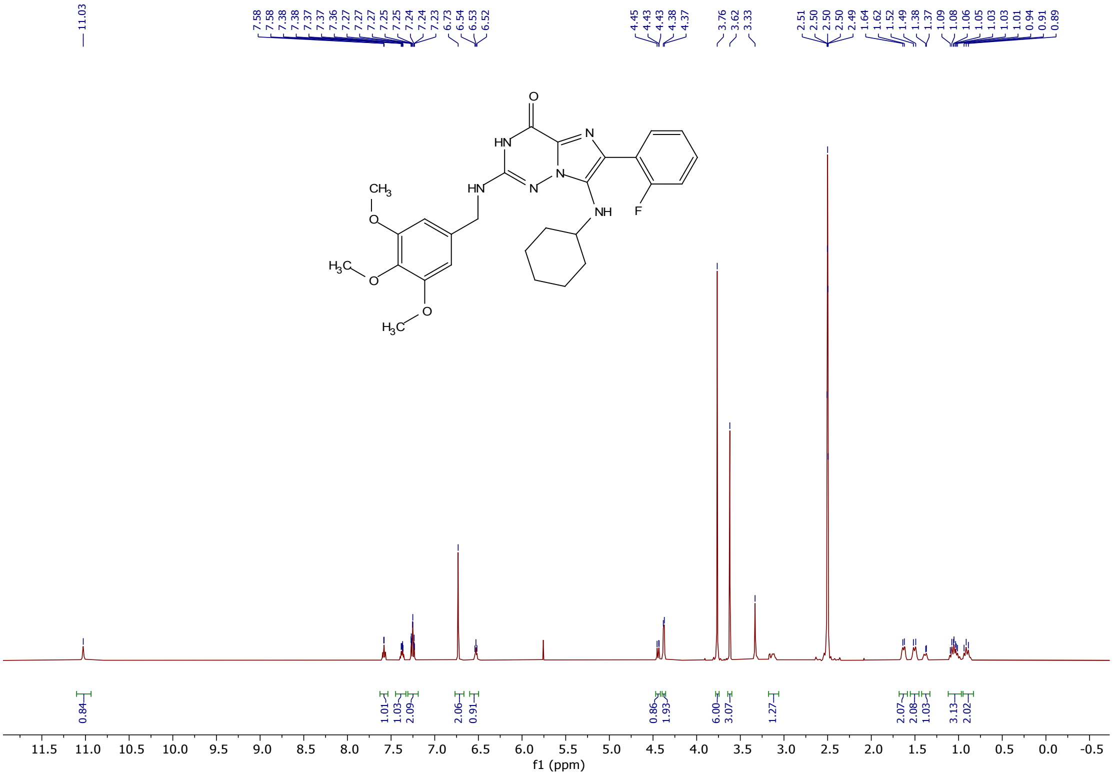

^{13}C{^{1}H} NMR spectrum of **5q** (126 MHz, DMSO-d_6)

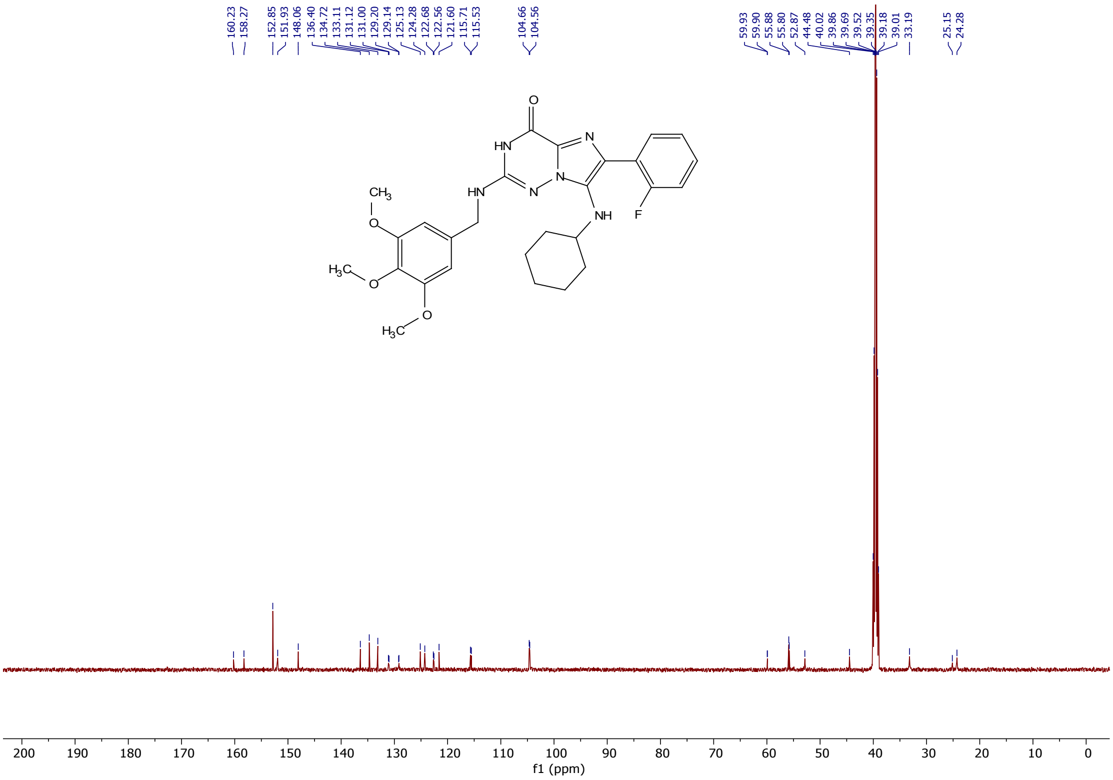

^{1}H NMR spectrum of **5r** (500 MHz, DMSO-d$_6$)

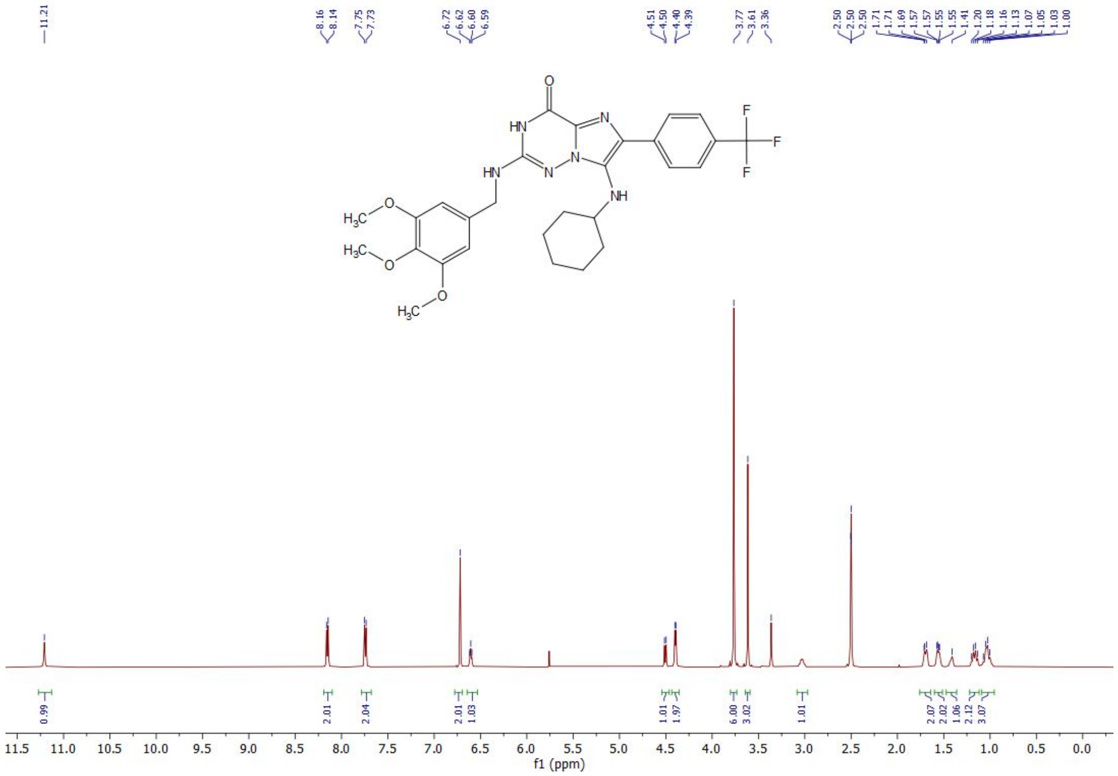

^{13}C{^{1}H} NMR spectrum of **5r** (126 MHz, DMSO-d$_6$)

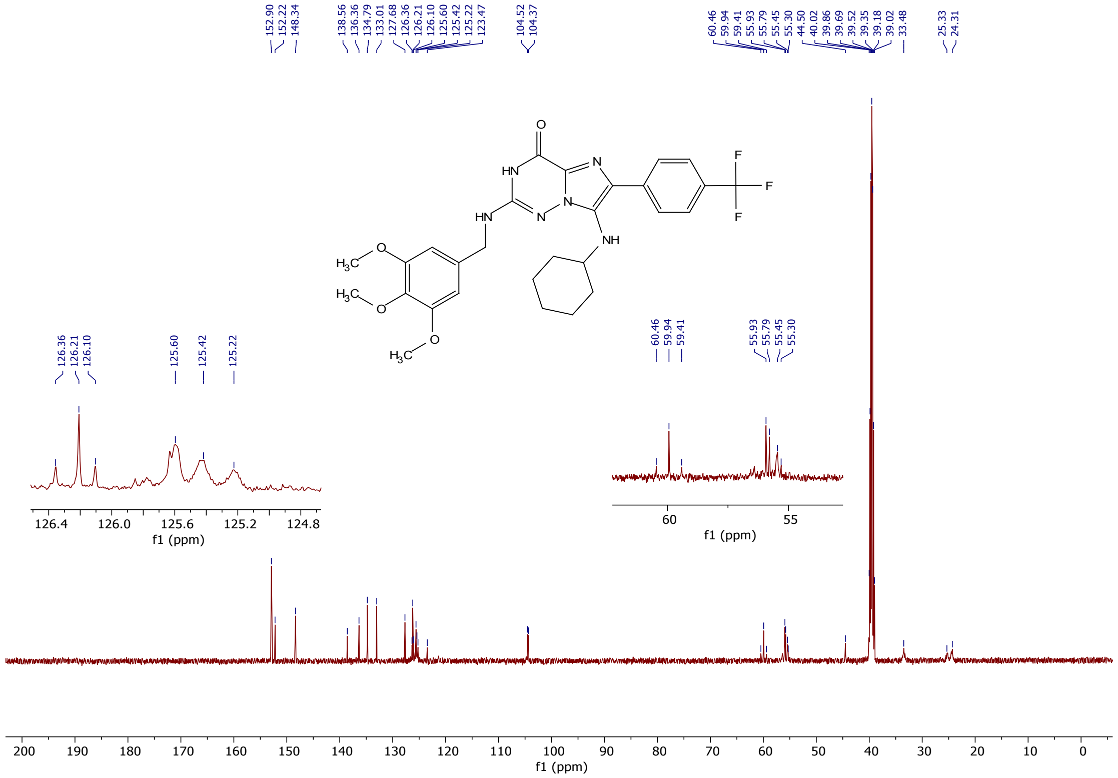

^{1}H NMR spectrum of **5s** (500 MHz, DMSO-d_6)

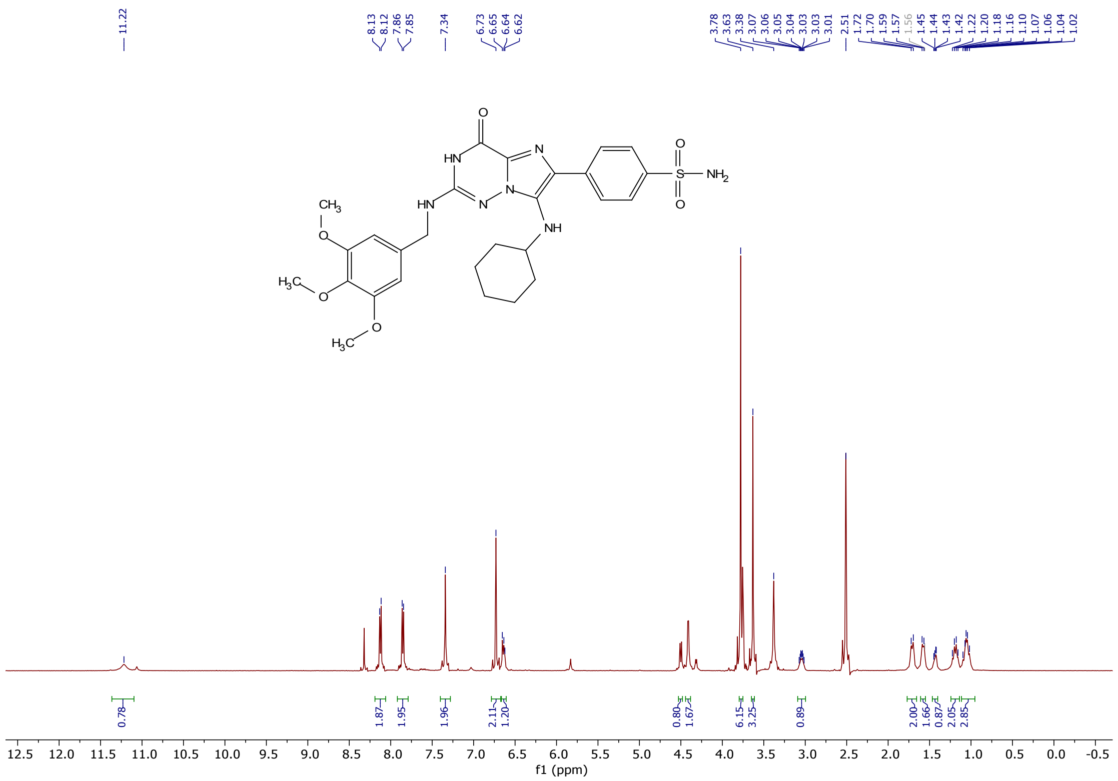

$^{13}C\{^{1}H\}$ NMR spectrum of **5s** (126 MHz, DMSO-d_6)

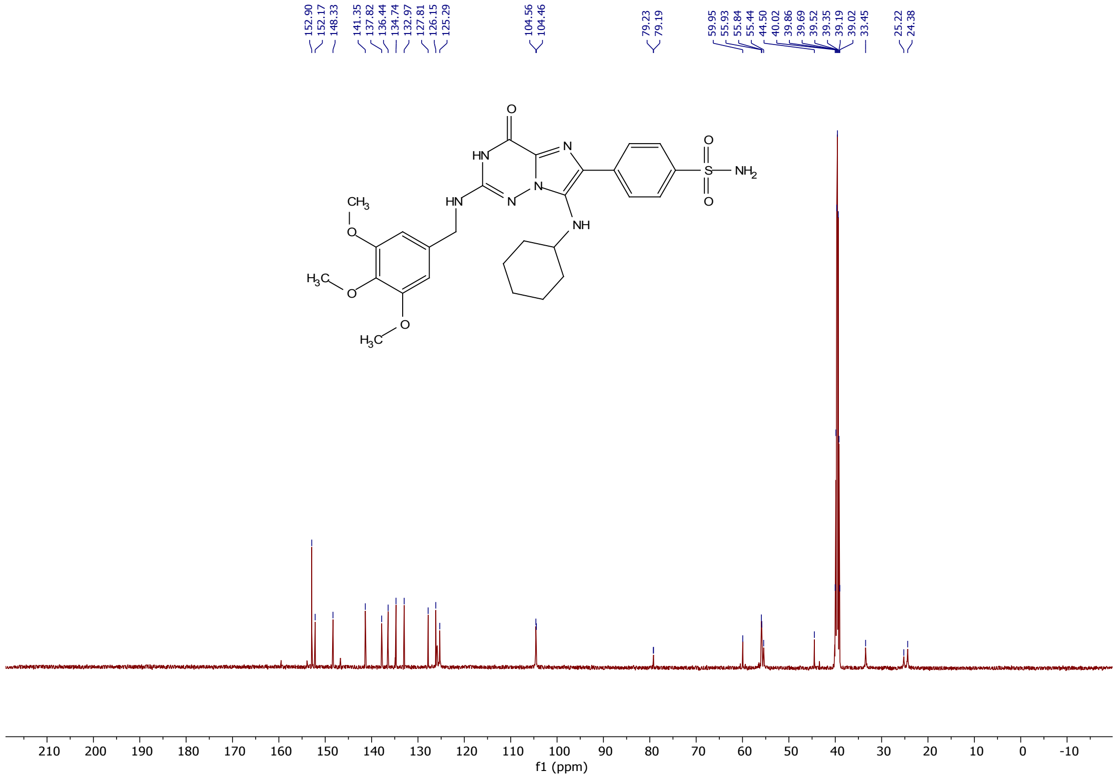

^{1}H NMR spectrum of **5u**(500 MHz, DMSO-d$_6$)

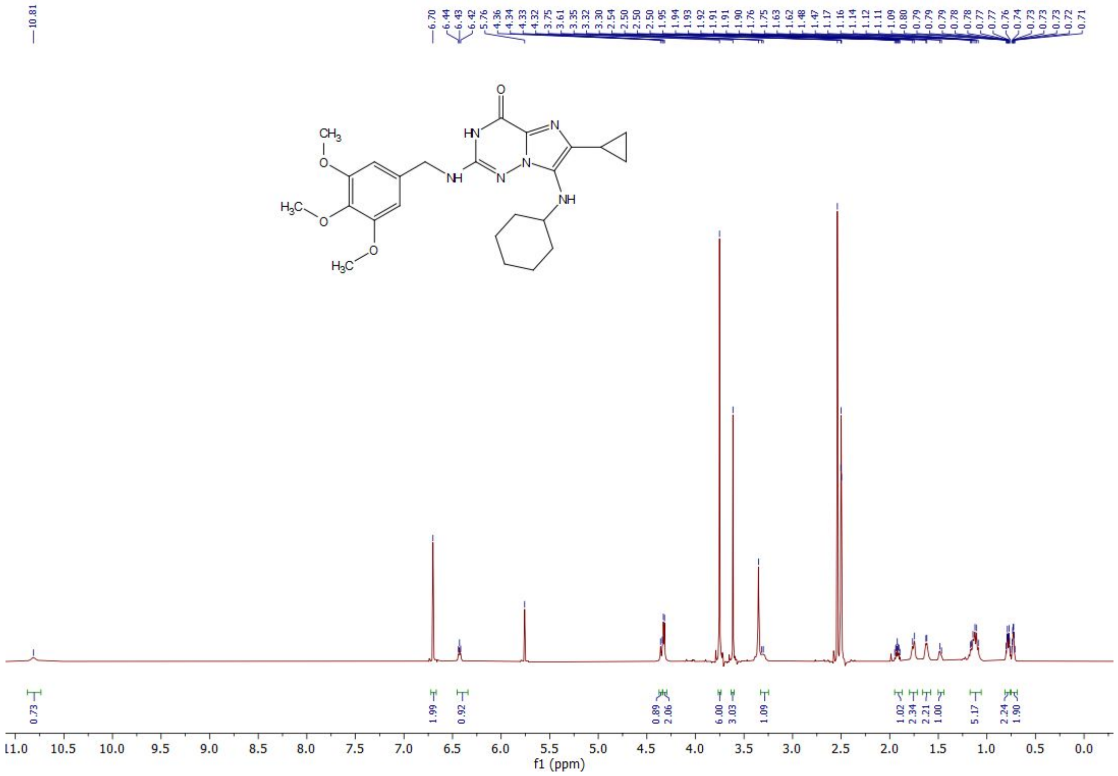

^{13}C{^{1}H} NMR spectrum of **5u** (126 MHz, DMSO-d$_6$)

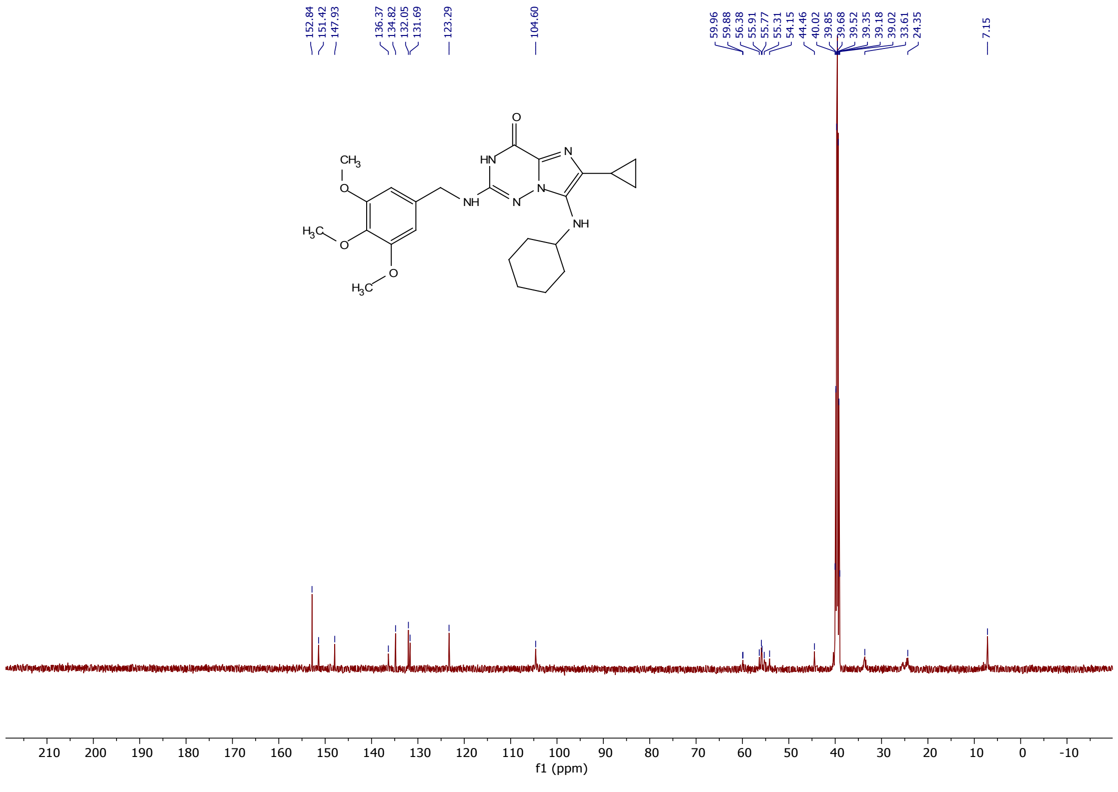

^{1}H NMR spectrum of **5v** (500 MHz, DMSO-d_6)

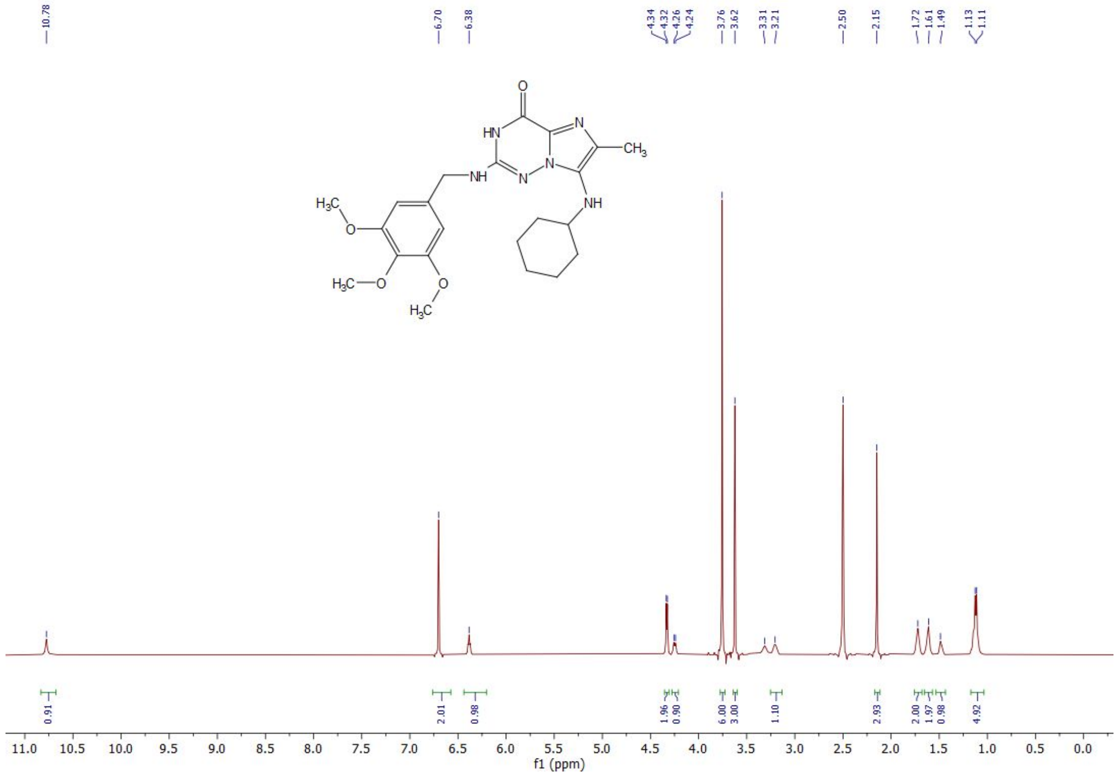

^{13}C{^{1}H} NMR spectrum of **5v** (126 MHz, DMSO-d_6)

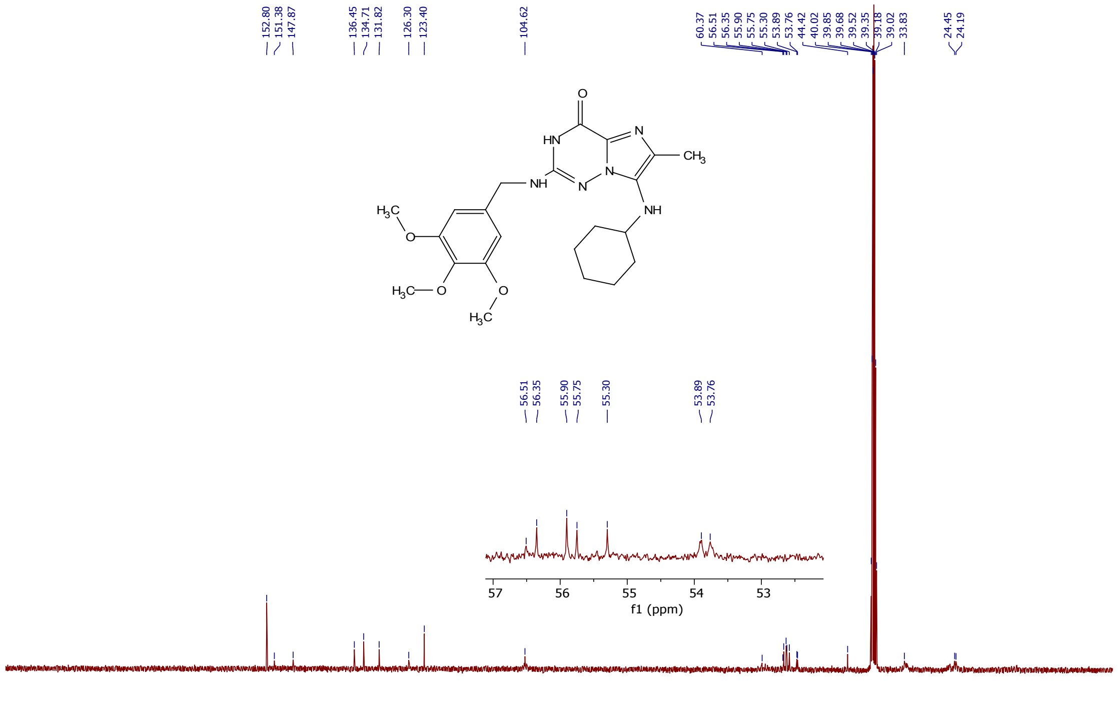

^{1}H NMR spectrum of **5w** (500 MHz, DMSO-d_6)

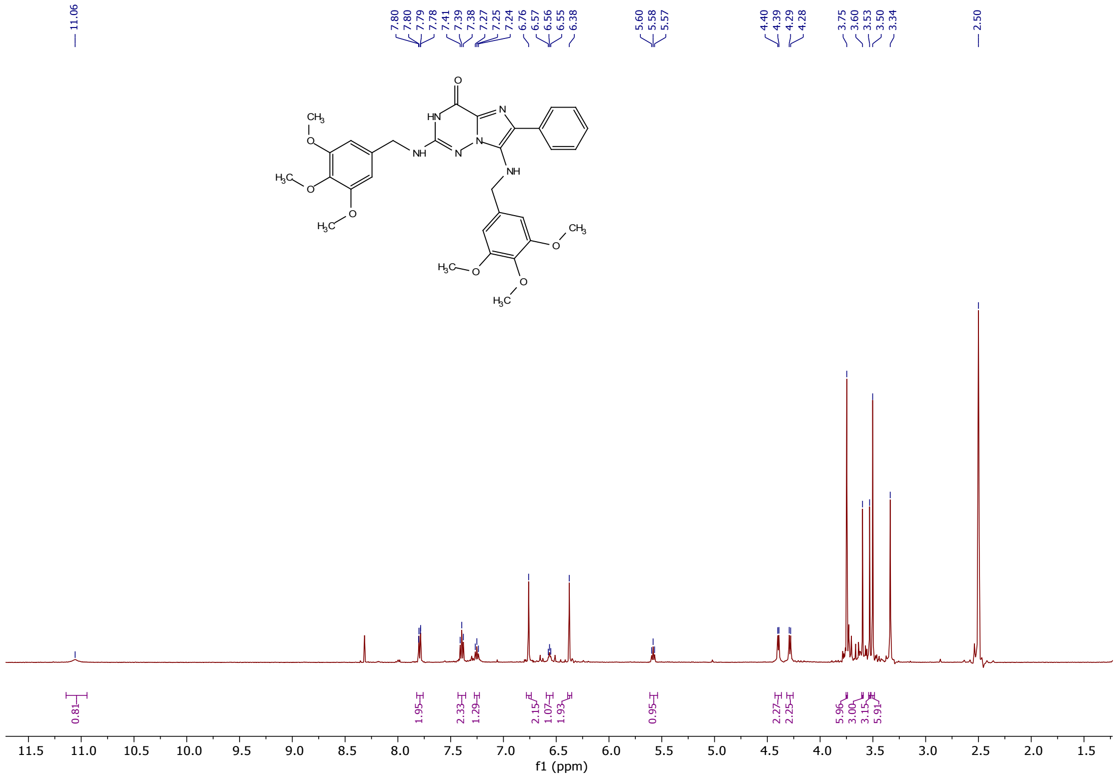

$^{13}C\{^{1}H\}$ NMR spectrum of **5w** (126 MHz, DMSO-d_6)

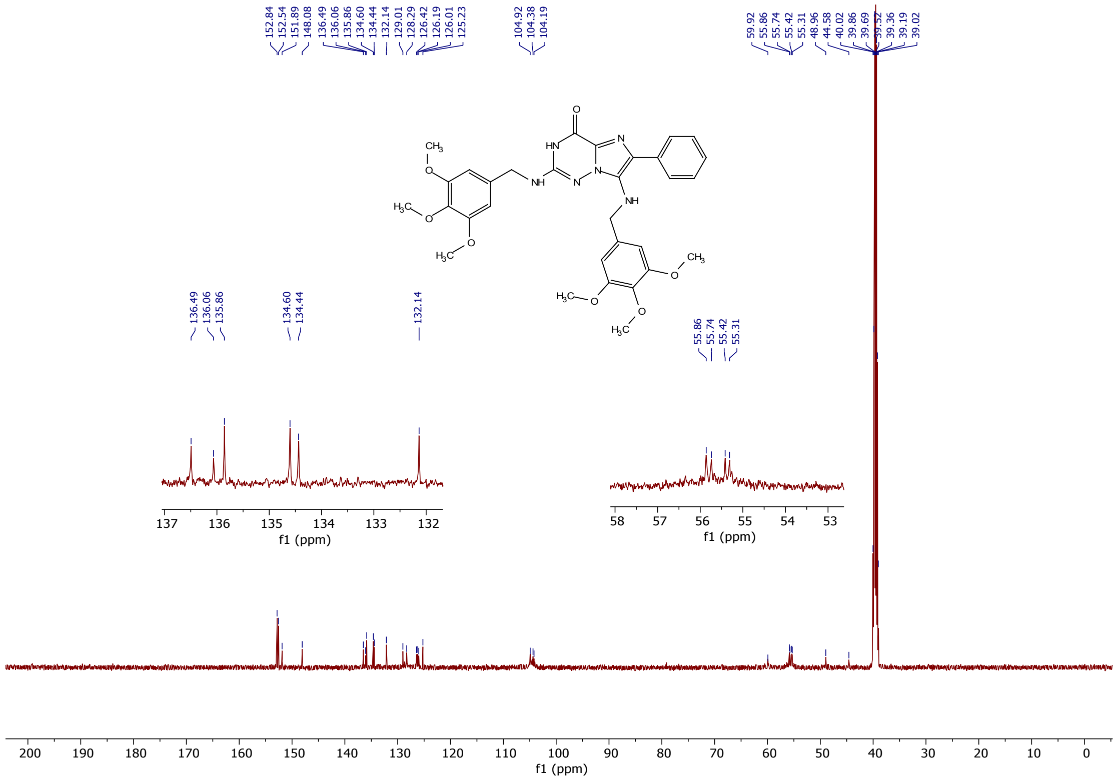

^{1}H NMR spectrum of **6a** (500 MHz, DMSO-d_6)

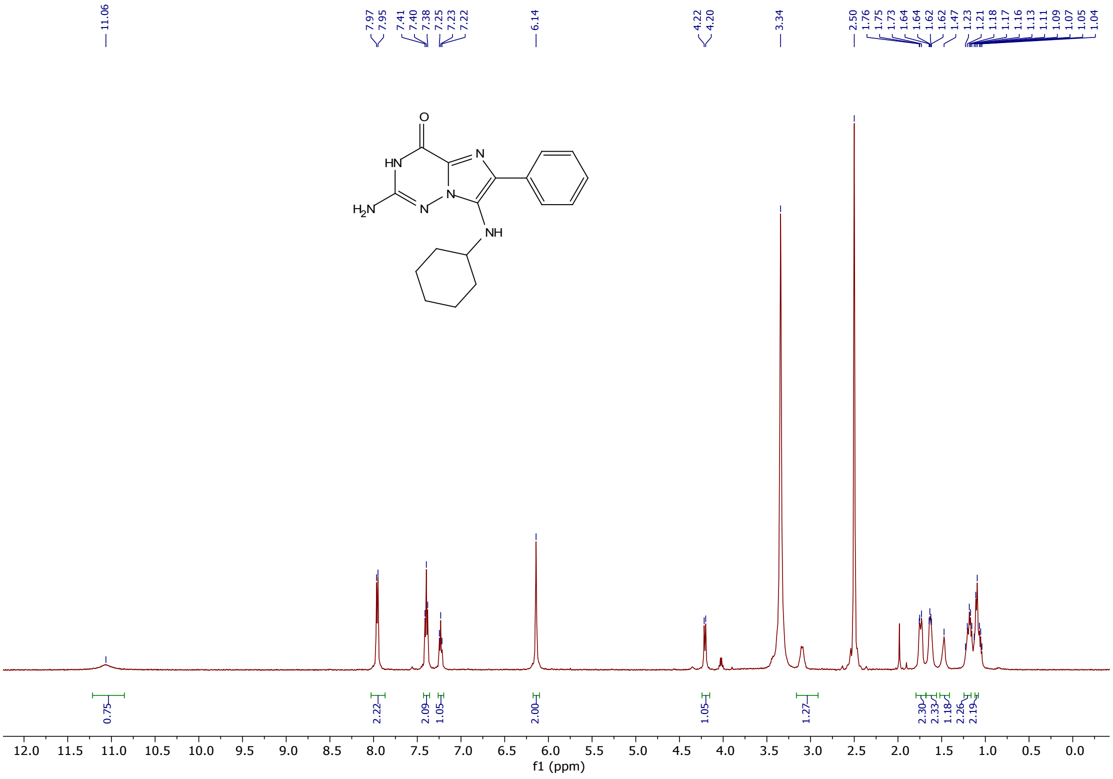

$^{13}C\{^{1}H\}$ NMR spectrum of **6a** (126 MHz, DMSO-d_6)

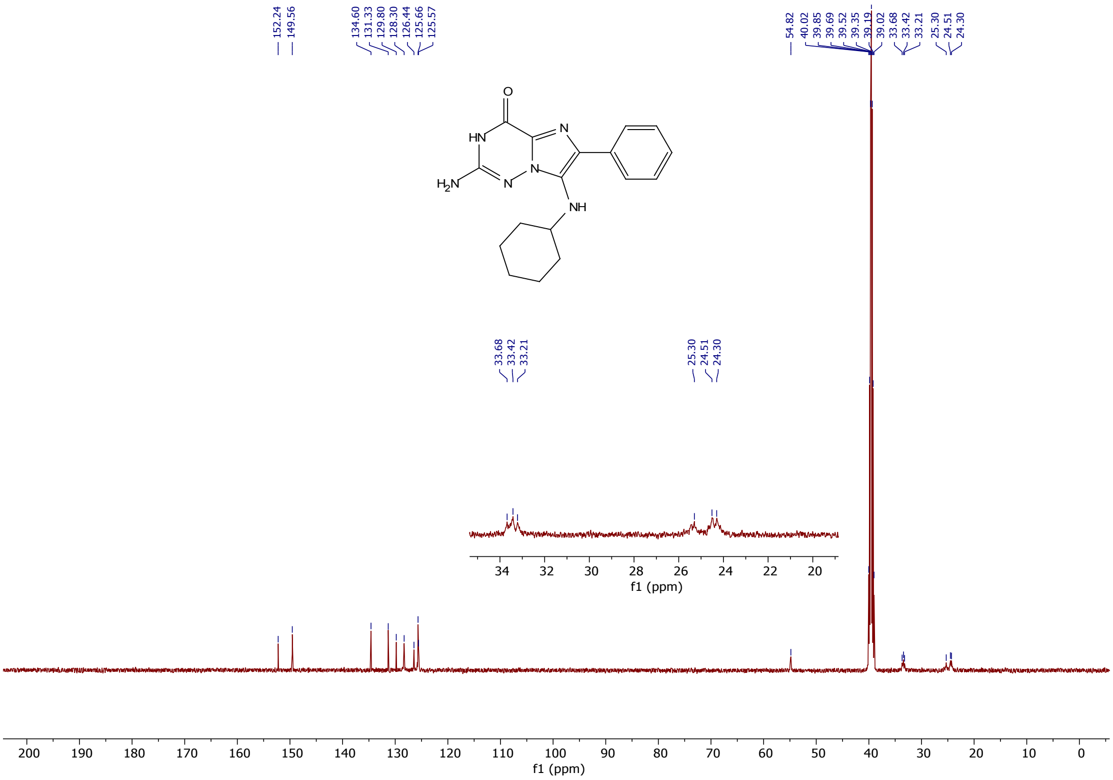

^{1}H NMR spectrum of **6b** (500 MHz, DMSO-d_6)

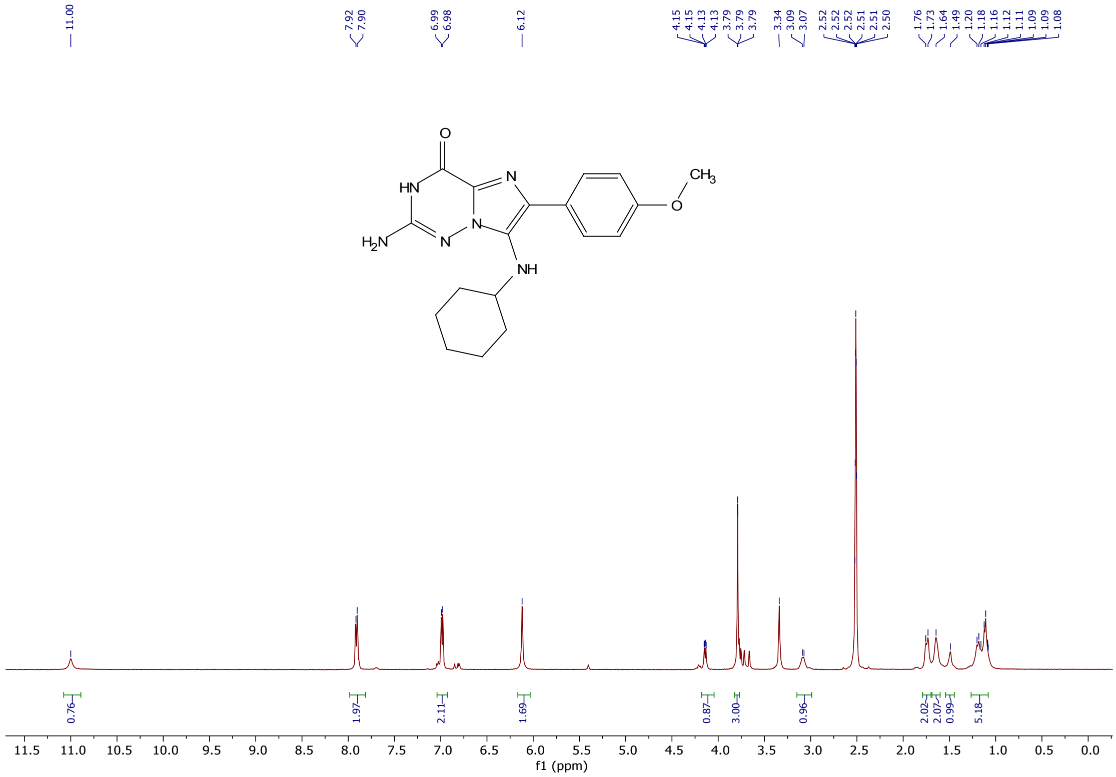

^{13}C{^{1}H} NMR spectrum of **6b** (126 MHz, DMSO-d_6)

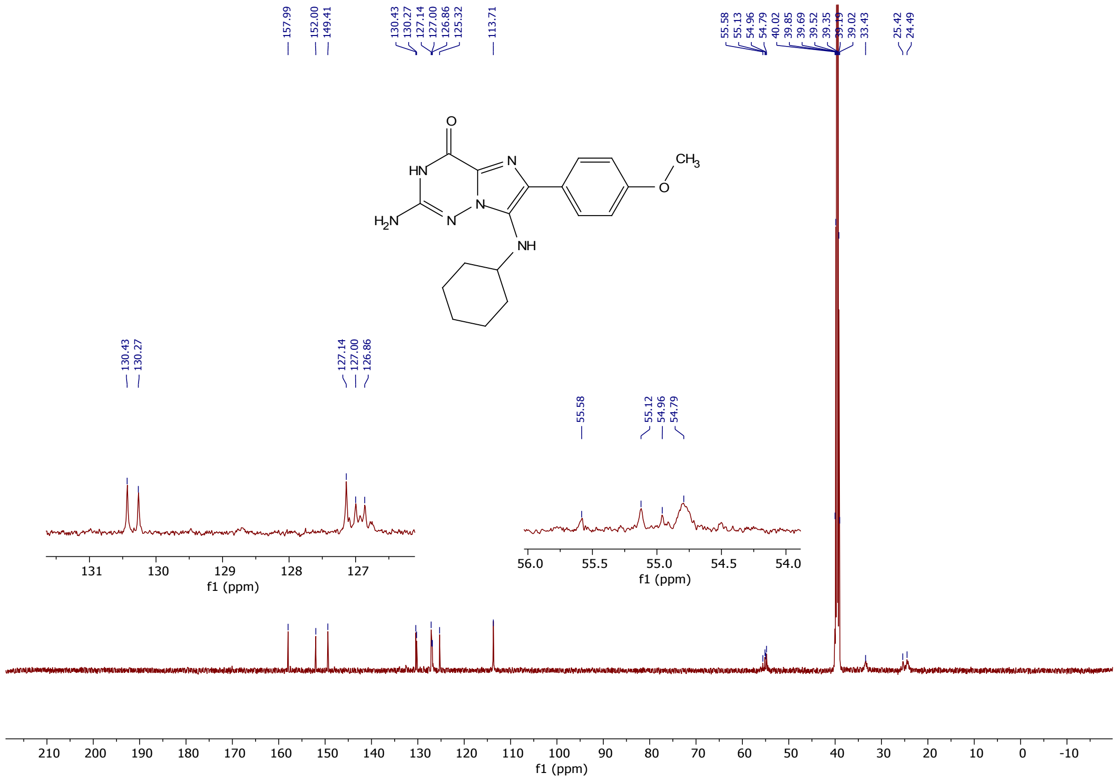

^{1}H NMR spectrum of **6c** (500 MHz, DMSO-d$_6$)

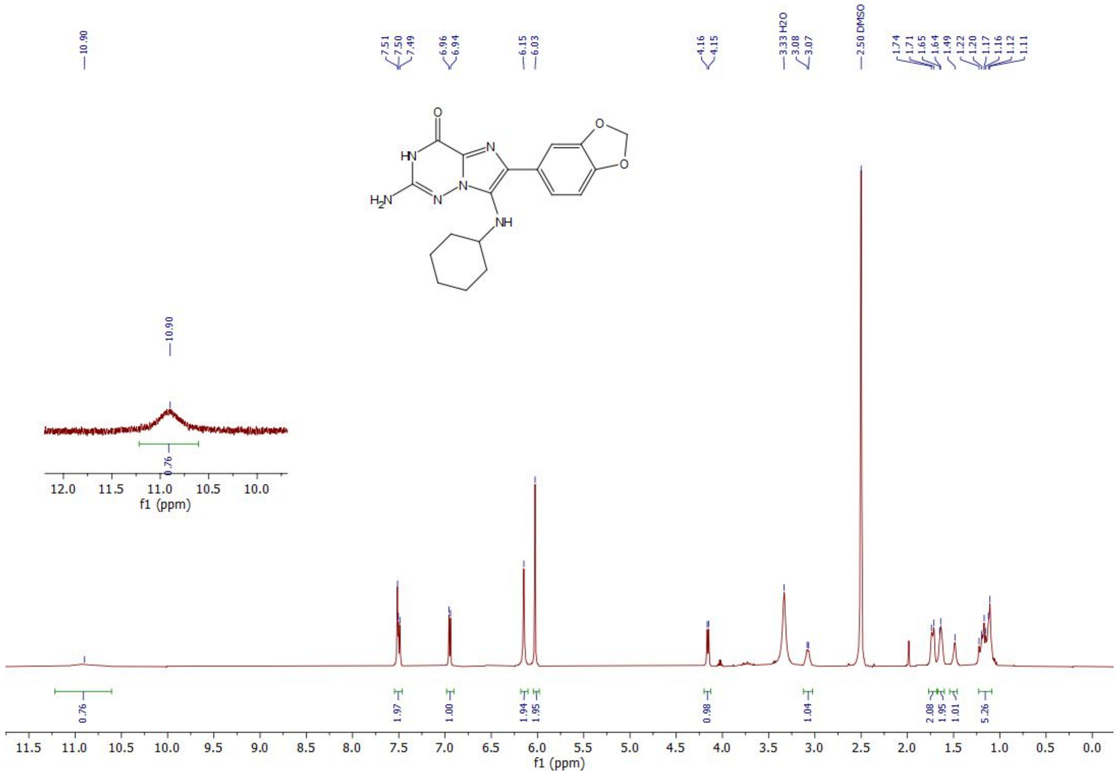

^{13}C{^{1}H} NMR spectrum of **6c** (126 MHz, DMSO-d$_6$)

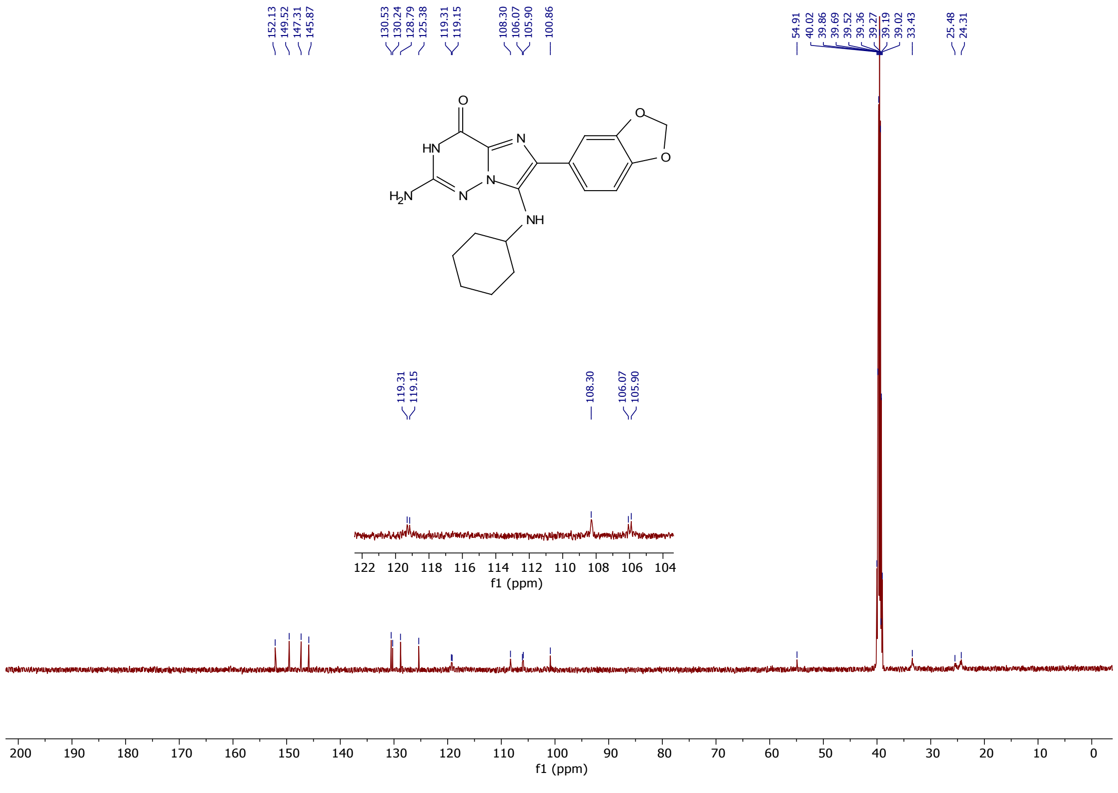

^{1}H NMR spectrum of **6d (6q)** (500 MHz, DMSO-d_6)

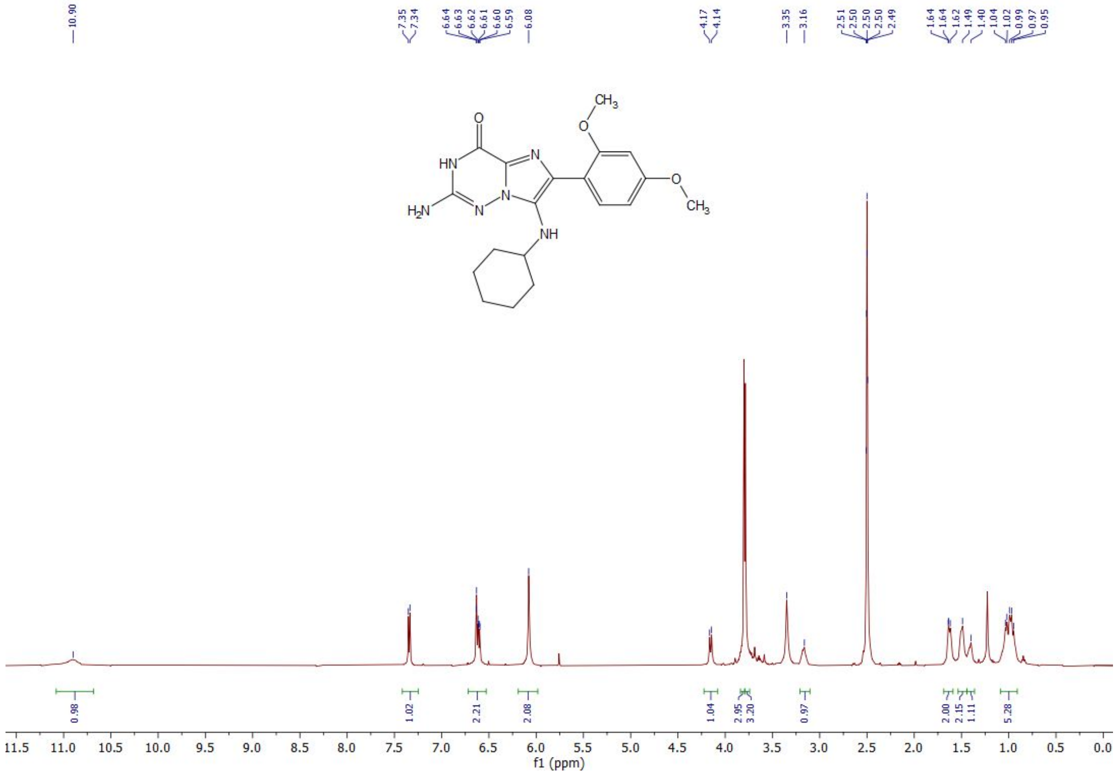

$^{13}C\{^{1}H\}$ NMR spectrum of **6d** (126 MHz, DMSO-d_6)

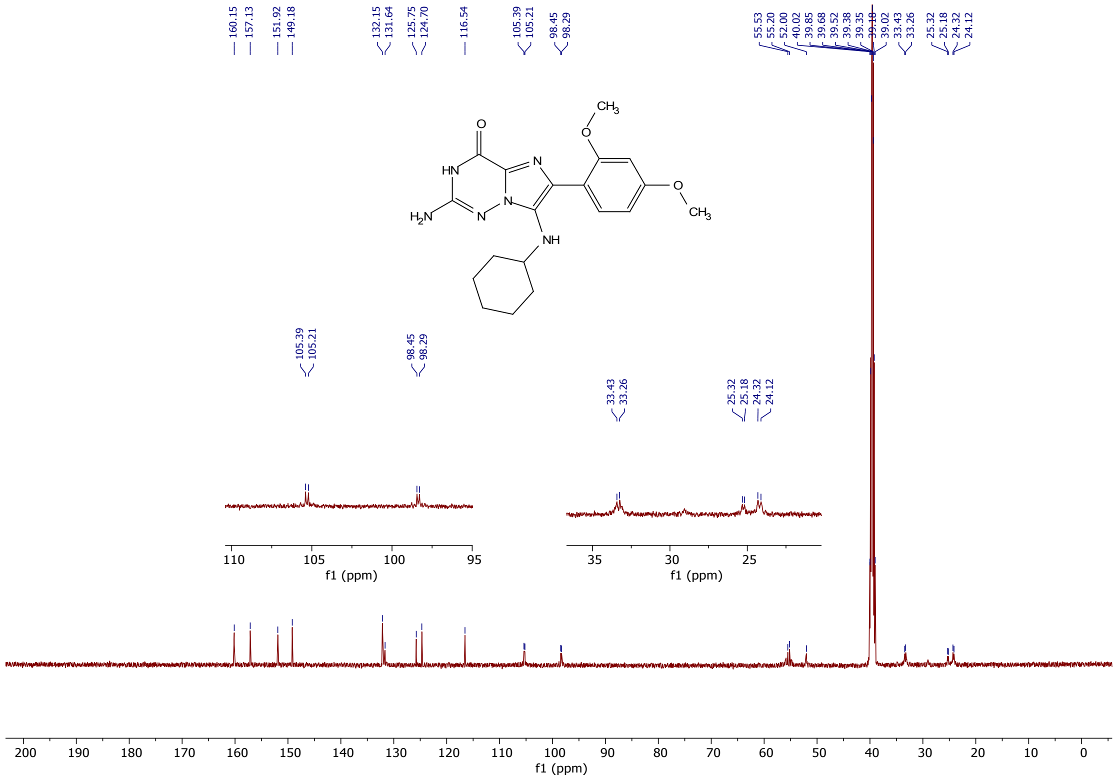

^{1}H NMR spectrum of **6e** (500 MHz, DMSO-d_6)

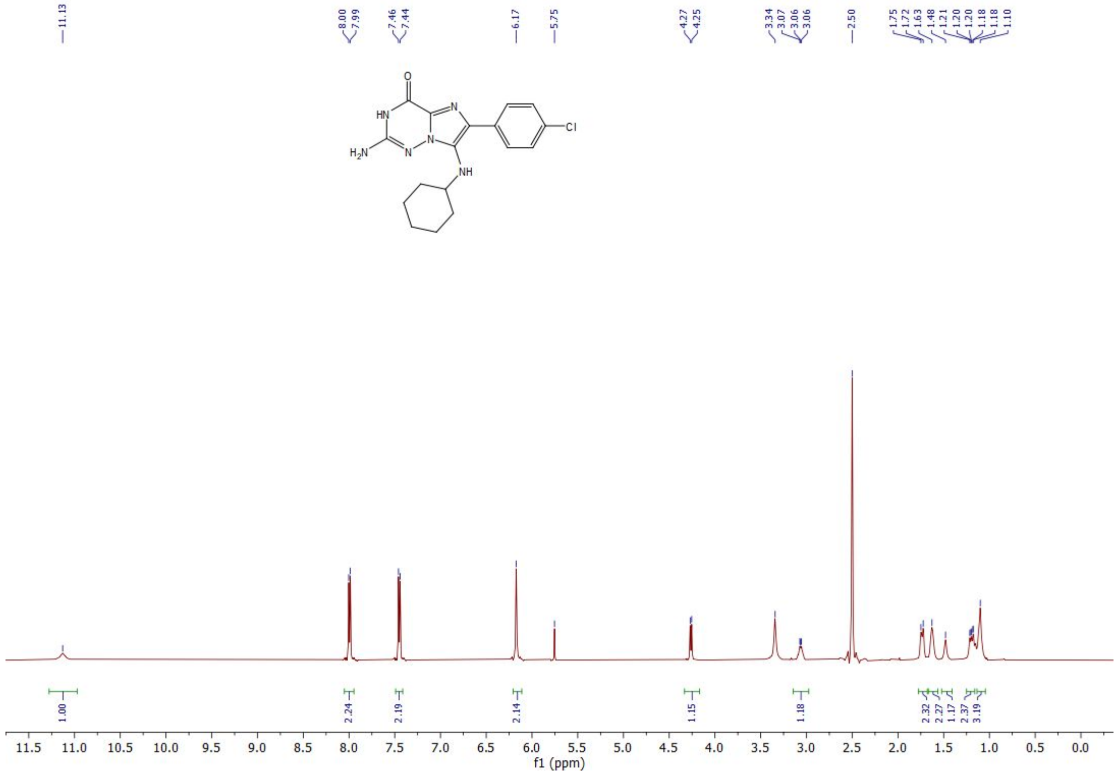

$^{13}C\{^{1}H\}$ NMR spectrum of **6e** (126 MHz, DMSO-d_6)

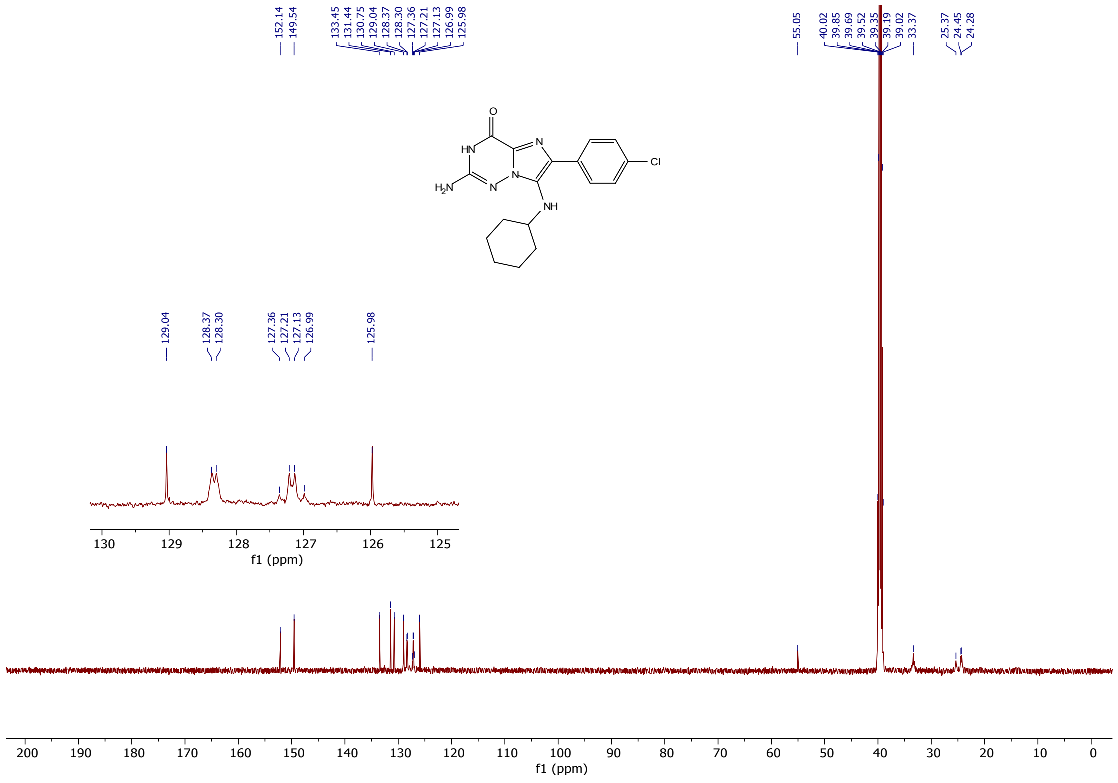

^{1}H NMR spectrum of **6f** (500 MHz, DMSO-d_6)

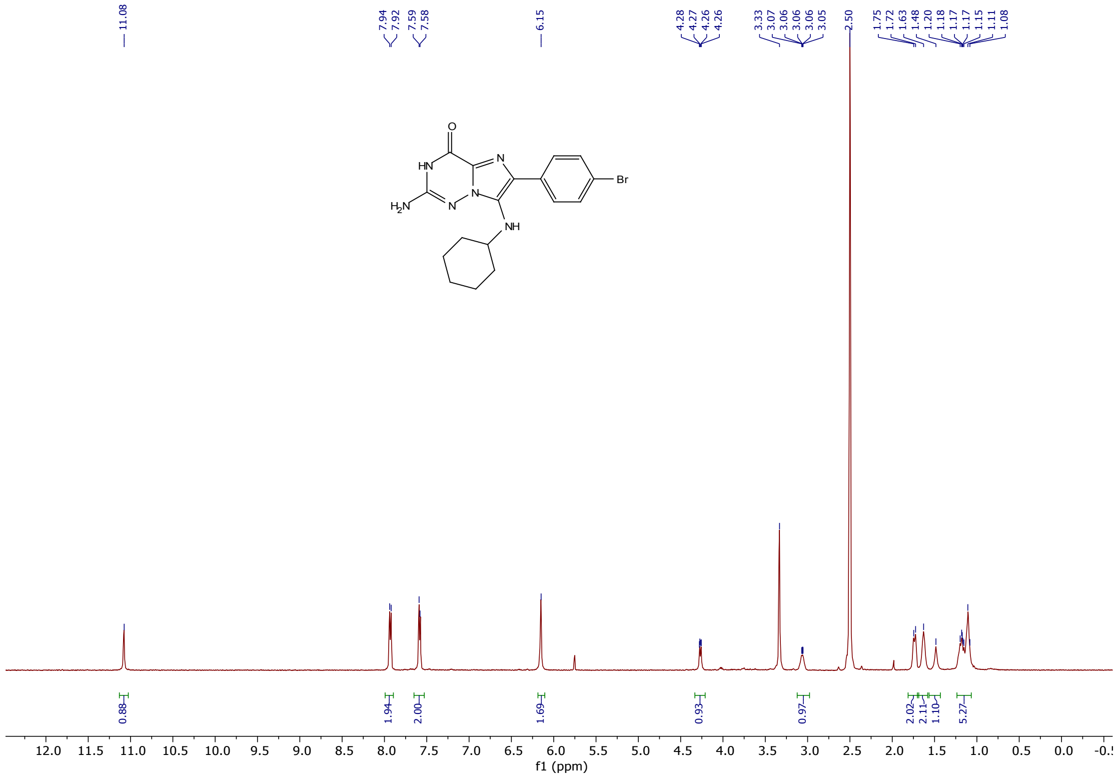

$^{13}C\{^{1}H\}$ NMR spectrum of **6f** (126 MHz, DMSO-d_6)

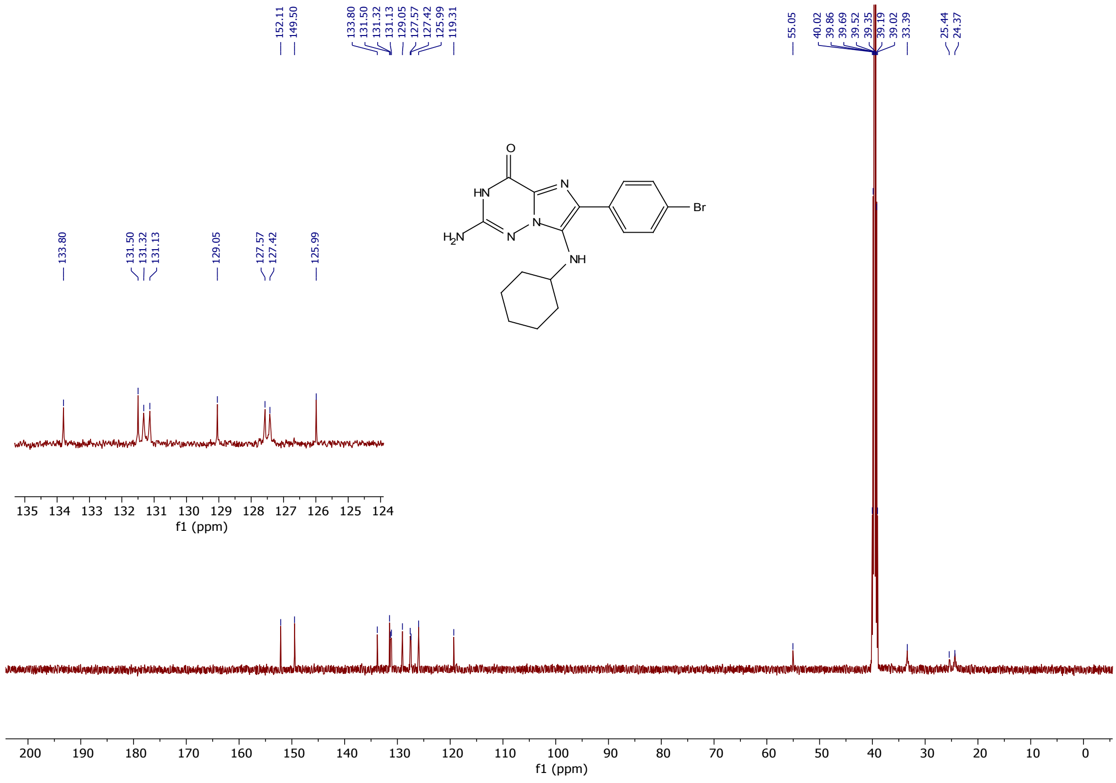

^{1}H NMR spectrum of **6b** (500 MHz, DMSO-d_6)

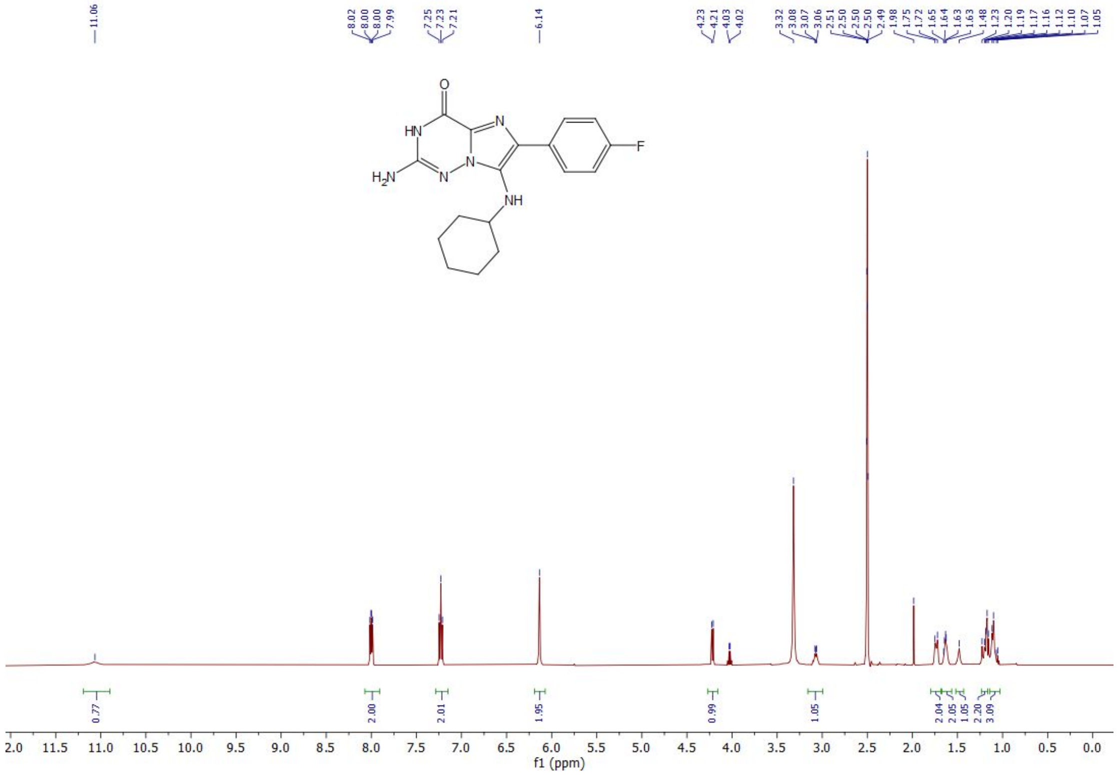

$^{13}C\{^{1}H\}$ NMR spectrum of **6g** (126 MHz, DMSO-d_6)

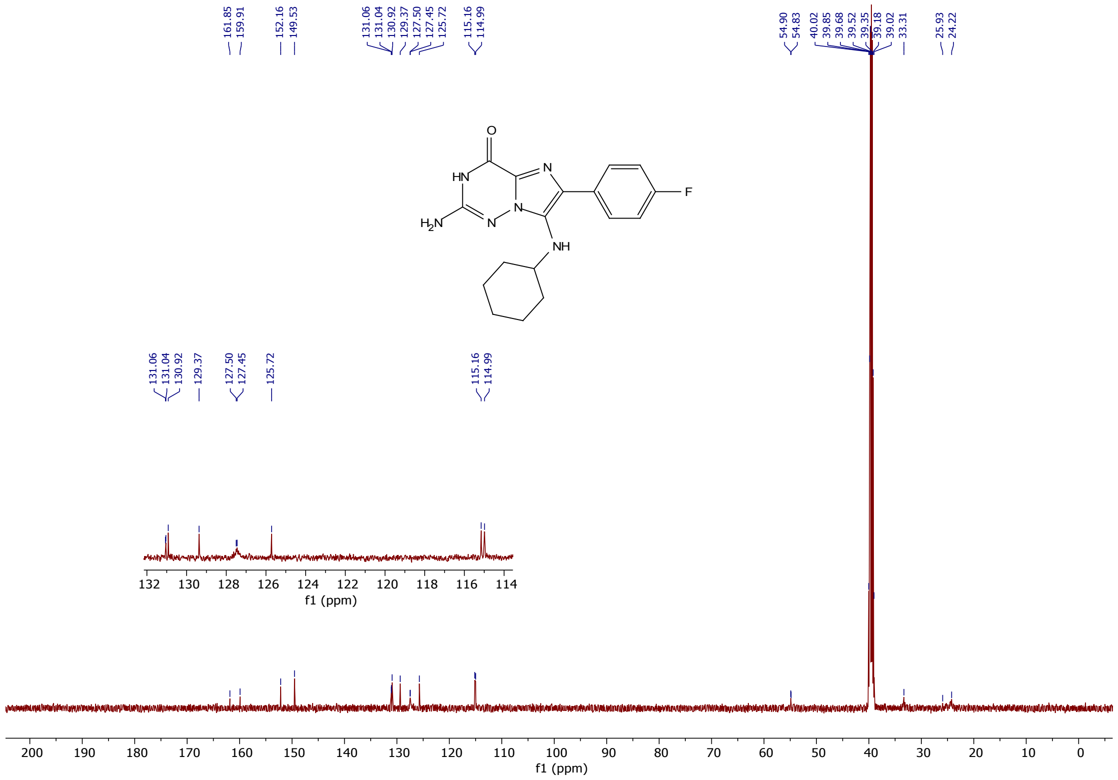

^{1}H NMR spectrum of **6h** (500 MHz, DMSO-d_6)

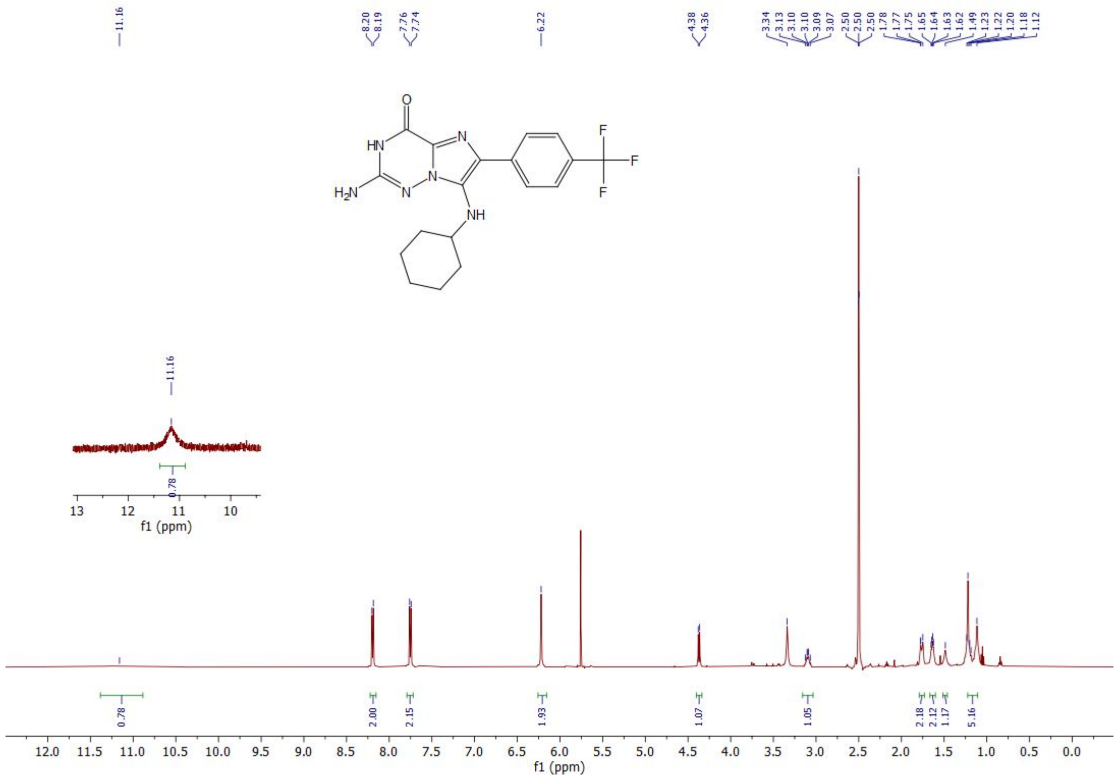

$^{13}C\{^1H\}$ NMR spectrum of **6h** (126 MHz, DMSO-d_6)

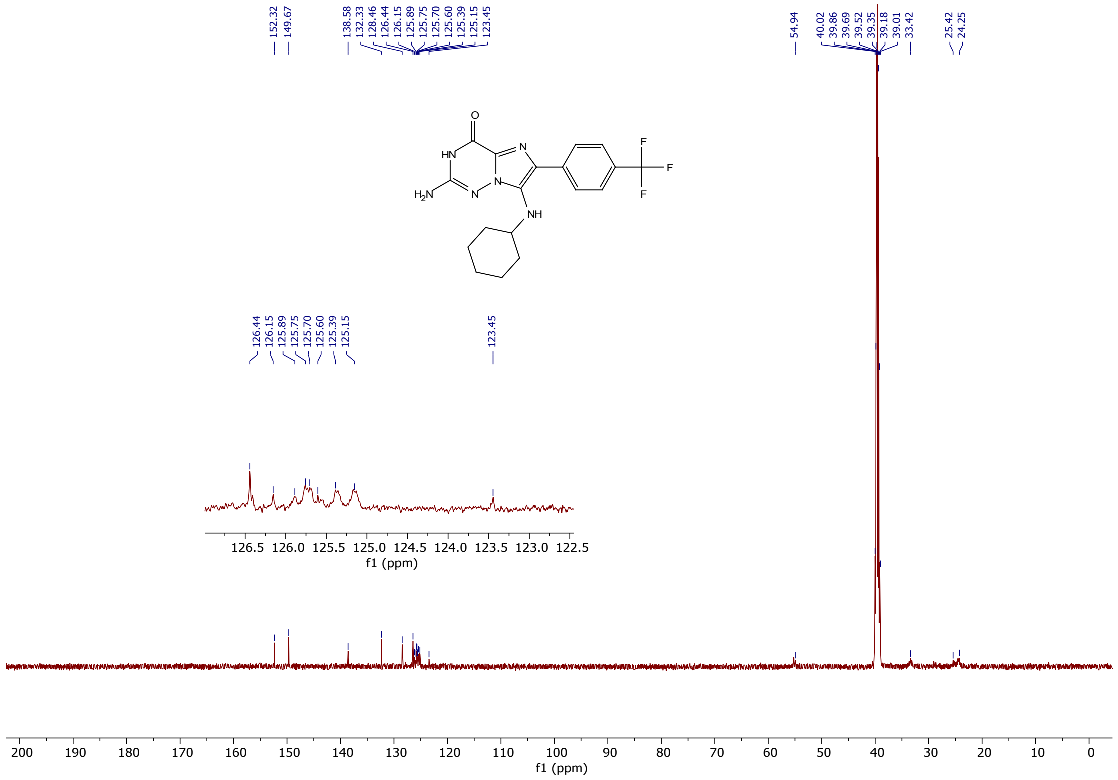

^{1}H NMR spectrum of **6i** (500 MHz, DMSO-d_6)

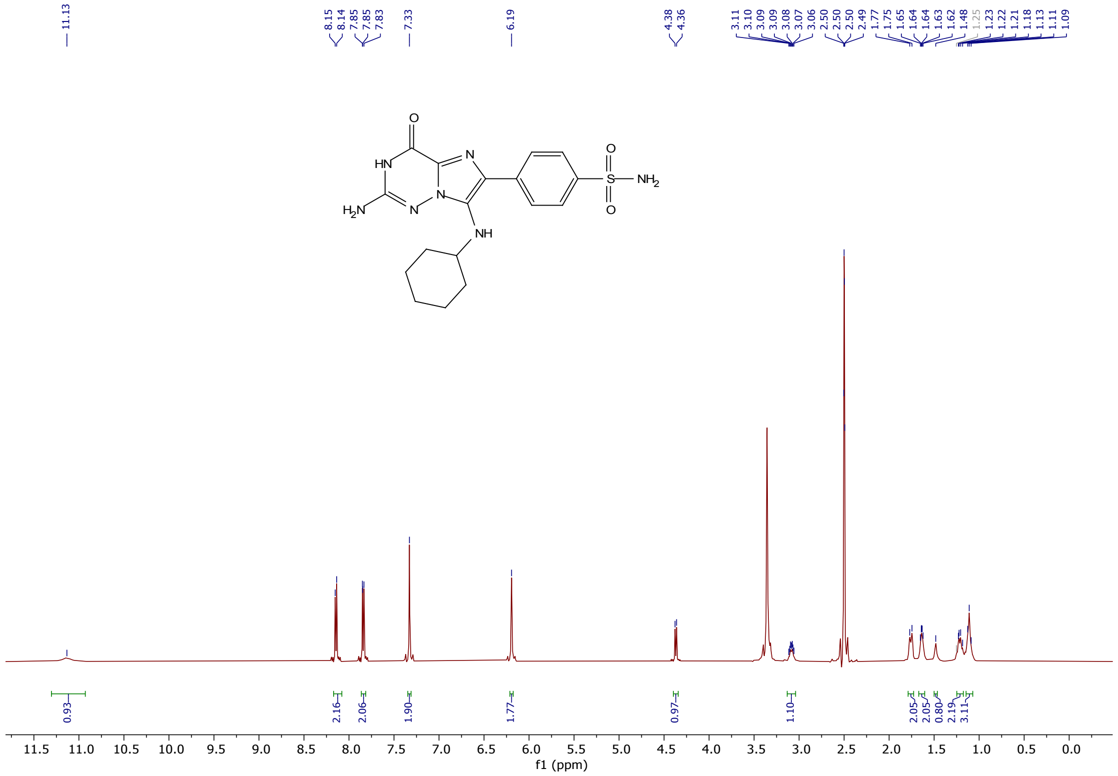

^{13}C{^{1}H} NMR spectrum of **6i** (126 MHz, DMSO-d_6)

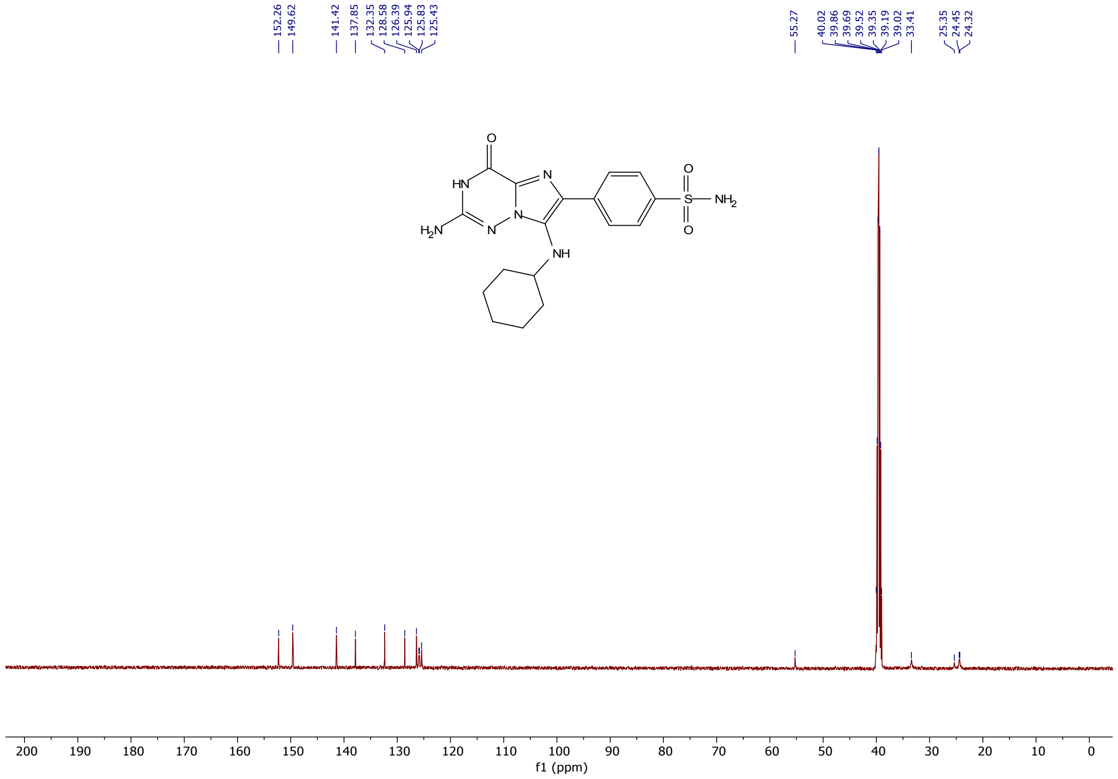

^{1}H NMR spectrum of **6j**(500 MHz, DMSO-d$_6$)

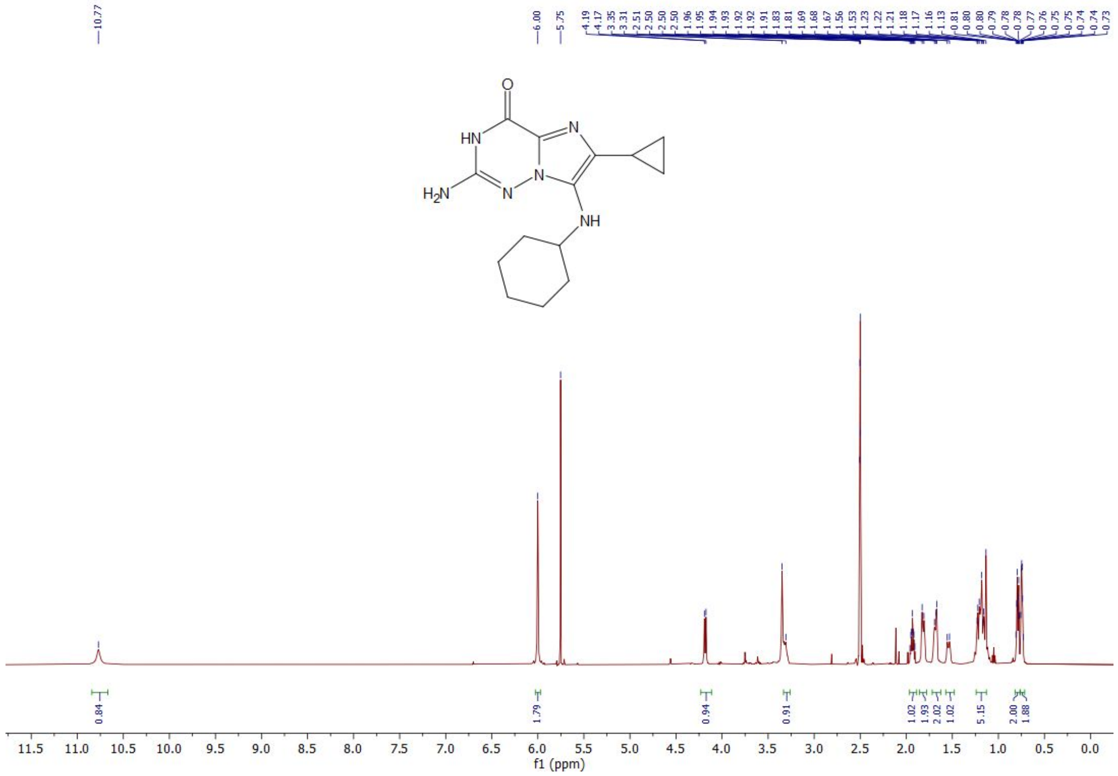

$^{13}C\{^{1}H\}$ NMR spectrum of **6j** (126 MHz, DMSO-d$_6$)

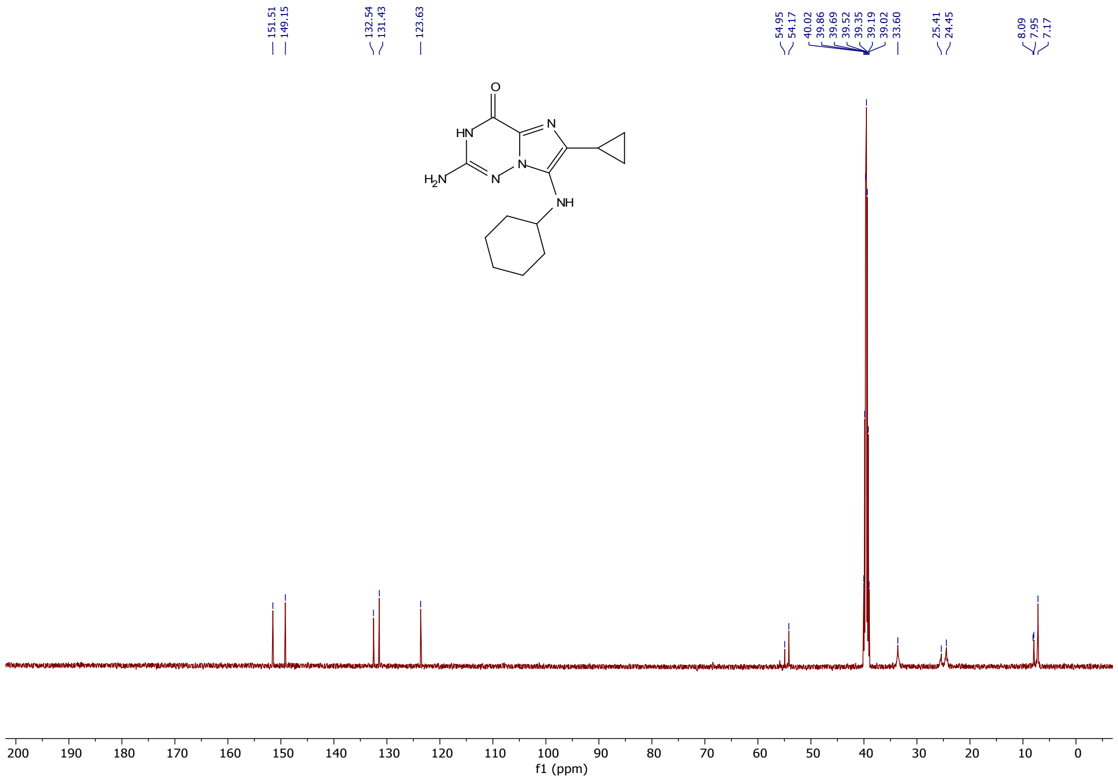

^{1}H NMR spectrum of **6l** (500 MHz, DMSO-d_6)

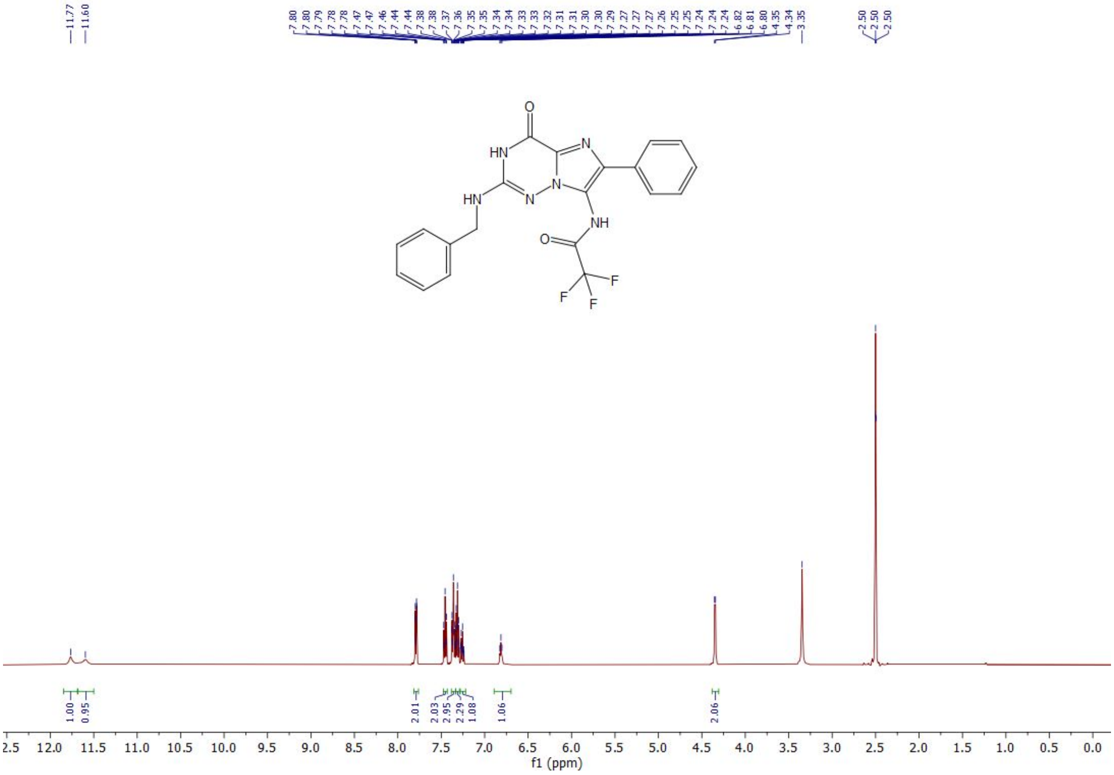

^{13}C{^{1}H} NMR spectrum of **6l** (126 MHz, DMSO-d_6)

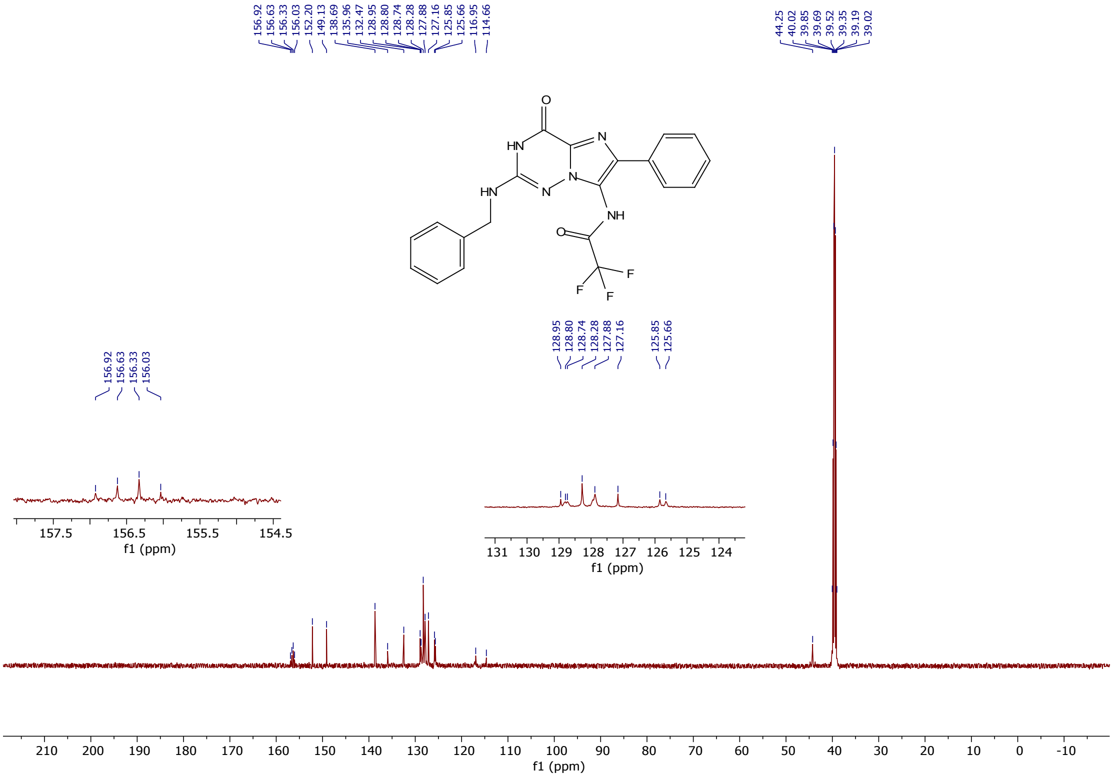

^{1}H NMR spectrum of **6m** (500 MHz, DMSO-d_6)

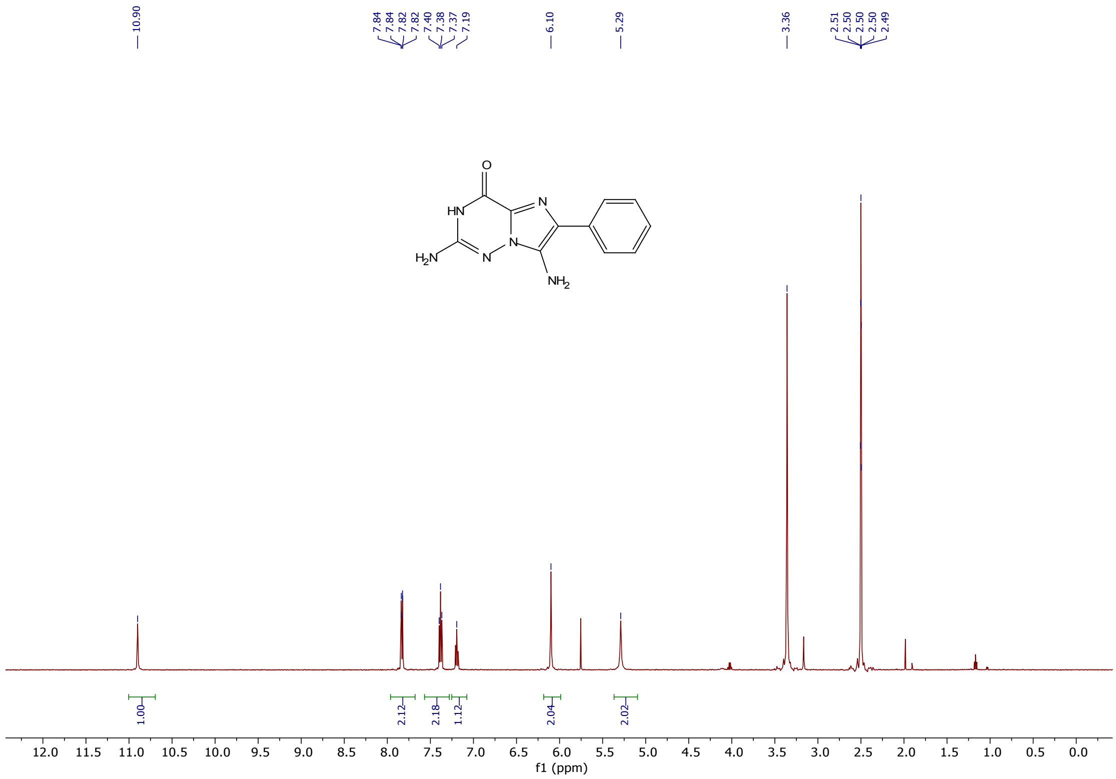

$^{13}C\{^{1}H\}$ NMR spectrum of **6m** (126 MHz, DMSO-d_6)

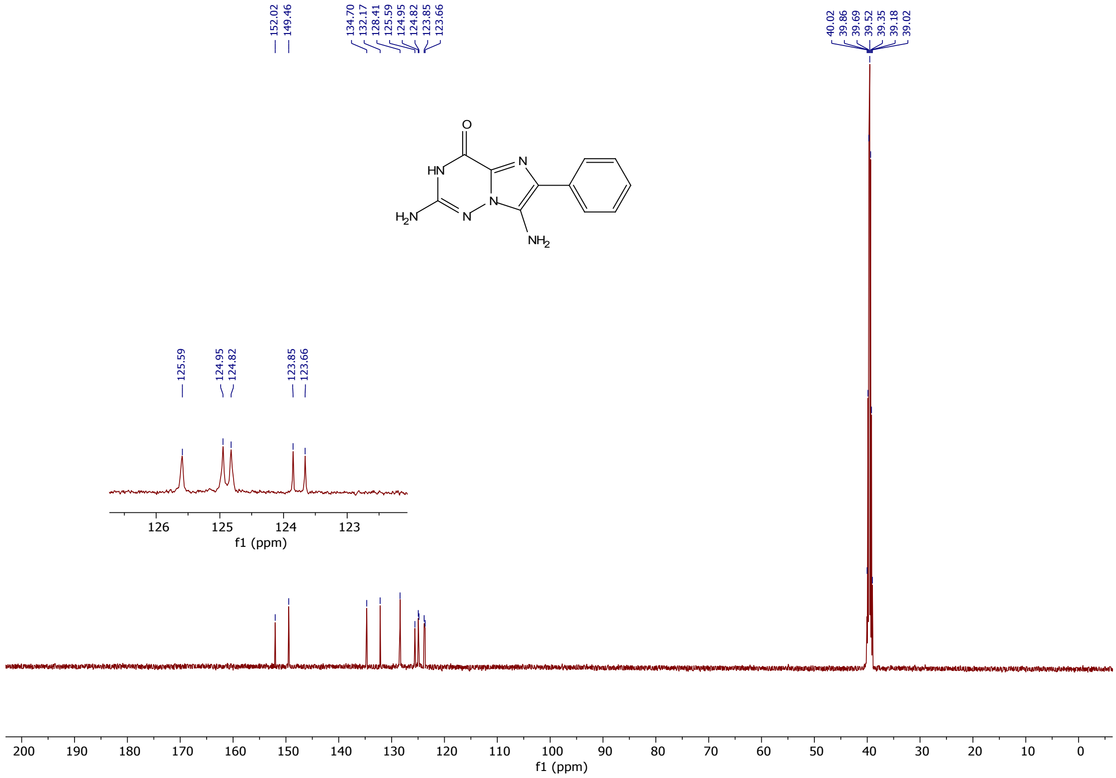

^{1}H NMR spectrum of **6n** (500 MHz, DMSO-d_6)

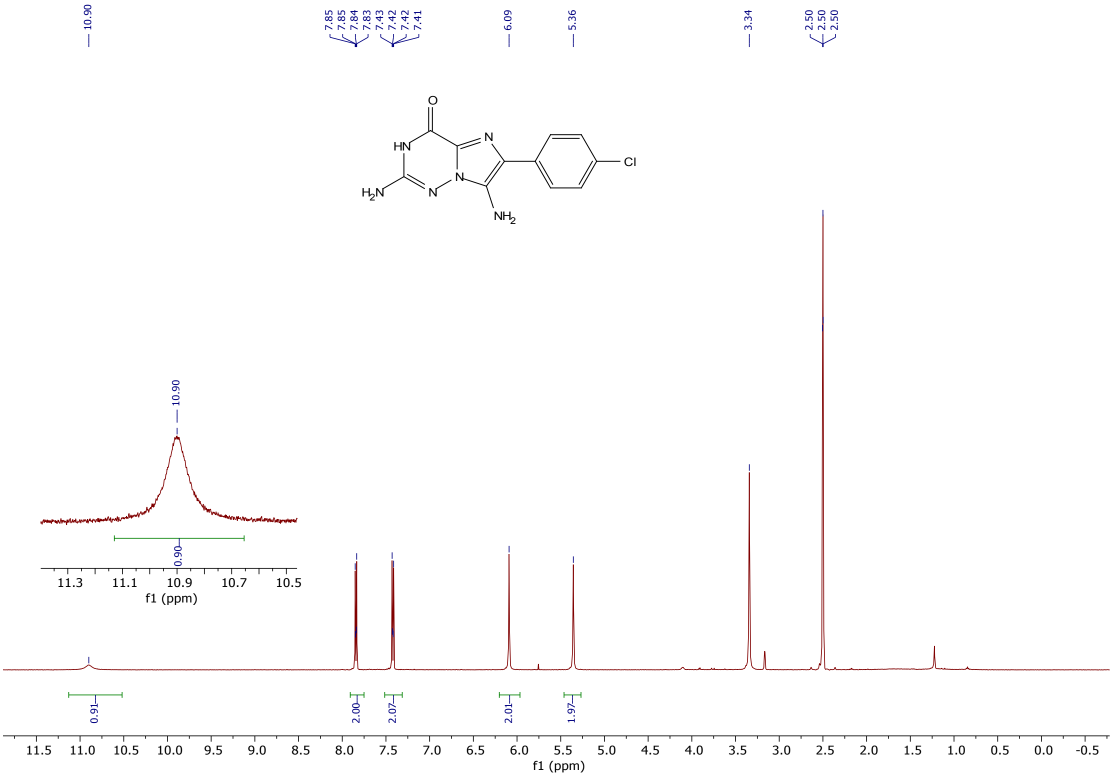

^{13}C{^{1}H} NMR spectrum of **6n** (126 MHz, DMSO-d_6)

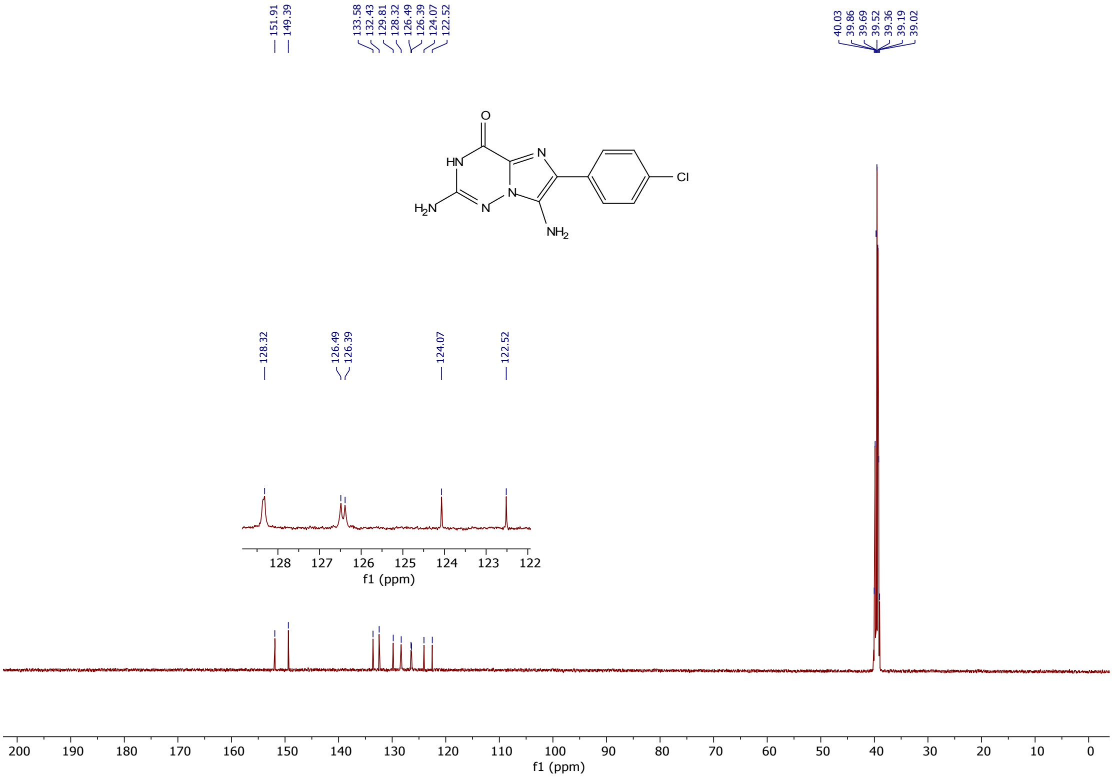

^{1}H NMR spectrum of **6o** (500 MHz, DMSO-d_6)

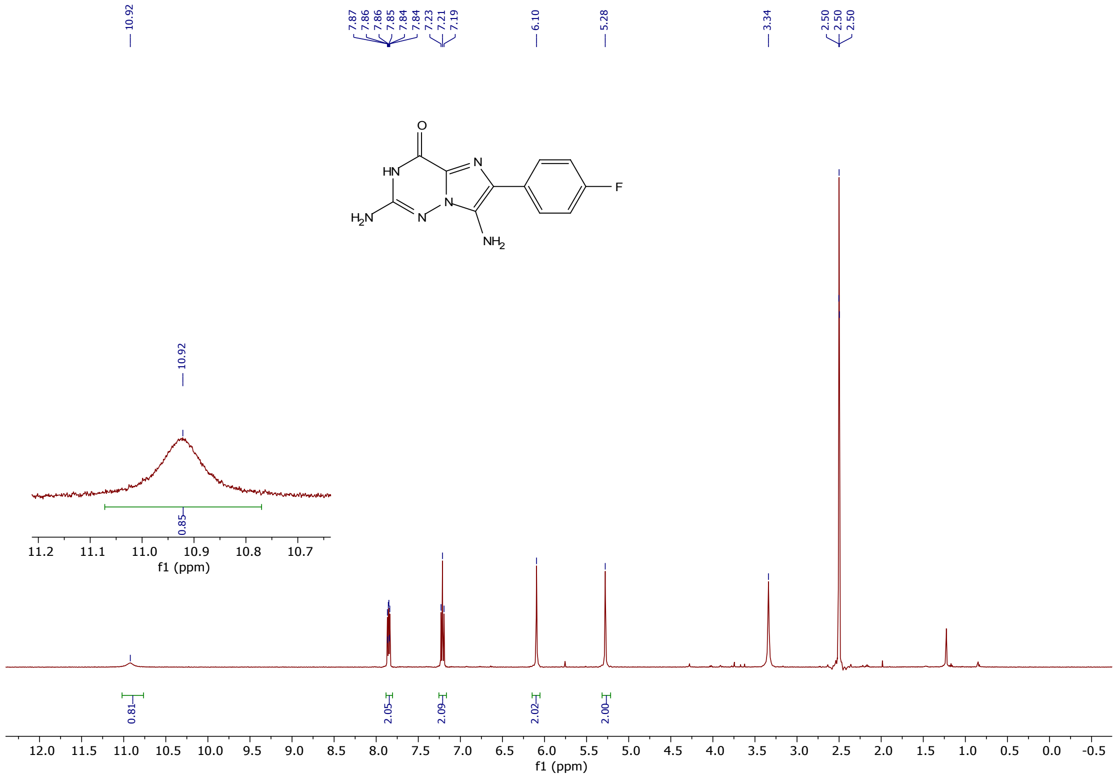

^{13}C{^{1}H} NMR spectrum of **6o** (126 MHz, DMSO-d_6)

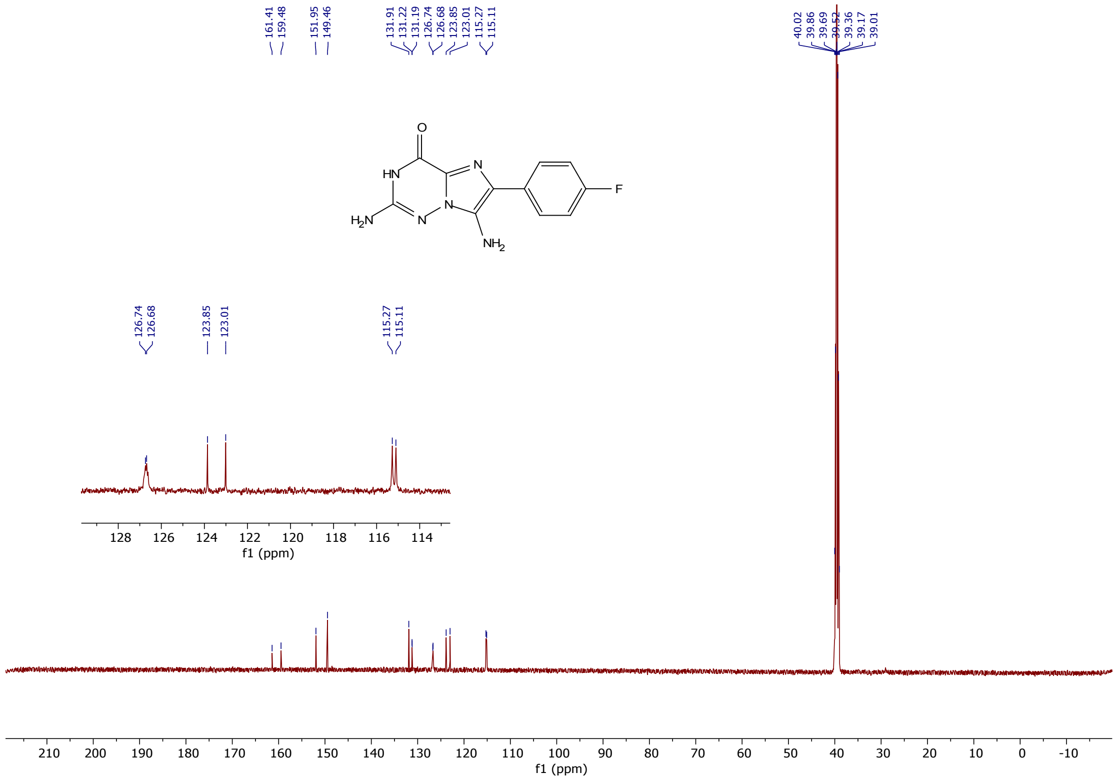

^{1}H NMR spectrum of **6p** (500 MHz, DMSO-d_6)

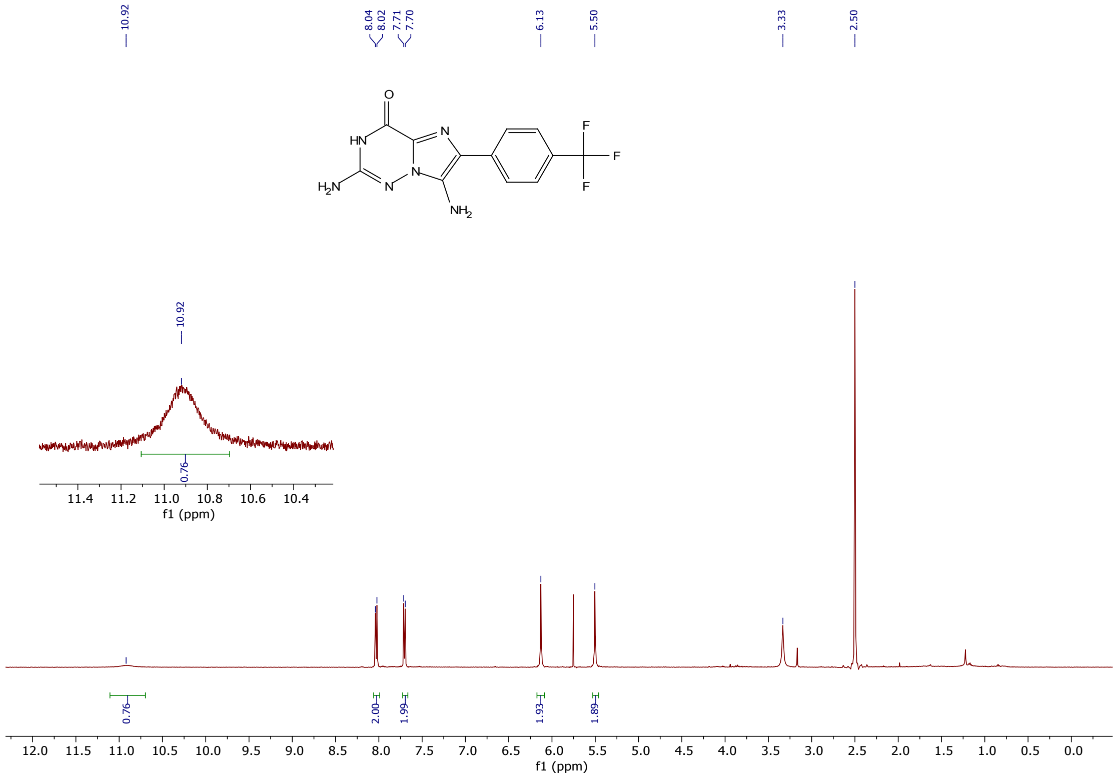

$^{13}C\{^{1}H\}$ NMR spectrum of **6p** (126 MHz, DMSO-d_6)

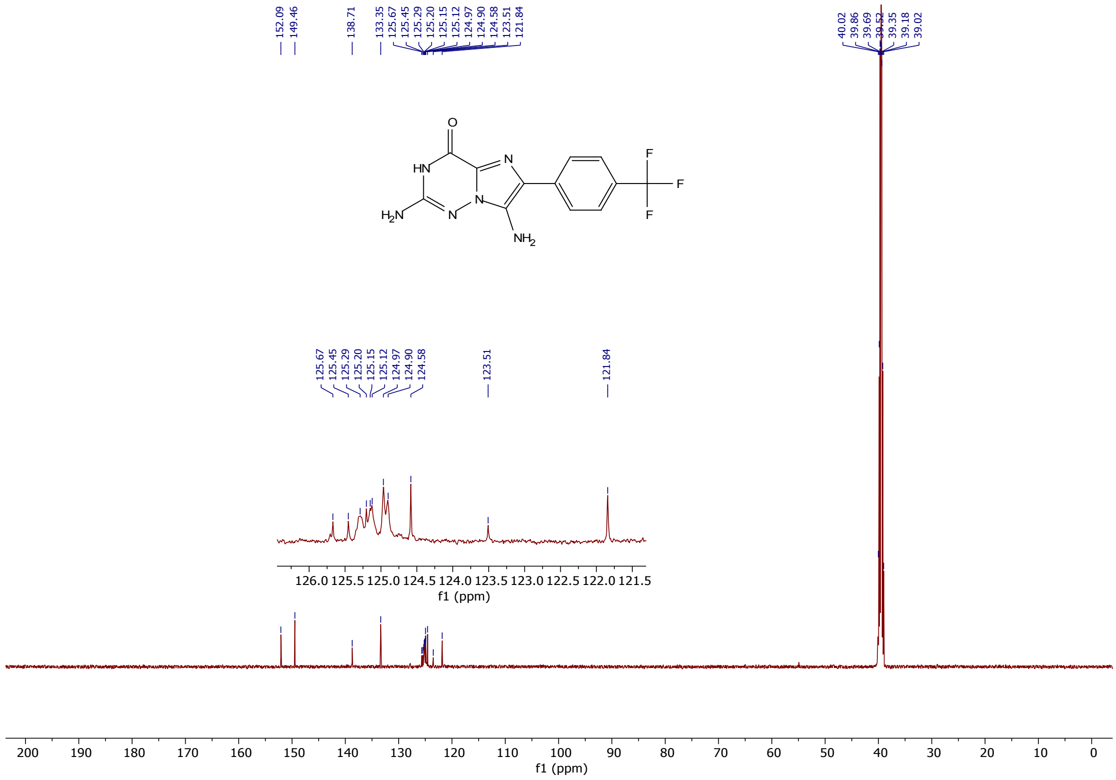

Supplement: Supplementary file 1 — jo3c00467_si_001.pdf [file jo3c00467_si_001.pdf]
